# Mono-/Bis-Alkenoic Acid Derivatives From an Endophytic Fungus *Scopulariopsis candelabrum* and Their Antifungal Activity

**DOI:** 10.3389/fchem.2021.812564

**Published:** 2022-01-11

**Authors:** Jun Tang, Xueshuang Huang, Ming-Hang Cao, Zhiyan Wang, Zhiyin Yu, Yijun Yan, Jian-Ping Huang, Li Wang, Sheng-Xiong Huang

**Affiliations:** ^1^ State Key Laboratory of Phytochemistry and Plant Resources in West China, CAS Center for Excellence in Molecular Plant Sciences, Kunming Institute of Botany, Chinese Academy of Sciences, Kunming, China; ^2^ Hunan Provincial Key Laboratory for Synthetic Biology of Traditional Chinese Medicine, Hunan University of Medicine, Huaihua, China; ^3^ Savaid Medical School, University of Chinese Academy of Sciences, Beijing, China; ^4^ State Key Laboratory of Southwestern Chinese Medicine Resources, Innovative Institute of Chinese Medicine and Pharmacy, Chengdu University of Traditional Chinese Medicine, Chengdu, China

**Keywords:** Alkenoic acid derivatives, polyketides, Microascaceae, *Scopulariopsis candelabrum*, antifungal activity, *Candida albicans*

## Abstract

During a screening for antifungal secondary metabolites, six new mono-/bis-alkenoic acid derivatives (**2**–**7**) and one known alkenoic acid derivative (**1**) were isolated from an endophytic fungi *Scopulariopsis candelabrum*. Their chemical structures were identified by ^1^H-NMR, ^13^C-NMR, 2D NMR, and high-resolution mass spectrometry, as well as comparisons with previously reported literatures. Among them, fusariumesters C‒F (**2**–**5**) are bis-alkenoic acid derivatives dimerized by an ester bond, while acetylfusaridioic acid A (**6**) and fusaridioic acid D (**7**) are alkenoic acid monomers. All the isolates were submitted to an antifungal assay against *Candida albicans* and the corn pathogen *Exserohilum turcicum* using the filter paper agar diffusion method. As a result, only compound **1** decorating with *β*-lactone ring turned out to be active against these two tested fungi. The broth microdilution assay against *Candida albicans* showed the minimum inhibitory concentration (MIC) value of **1** to be 20 *μ*g/ml, while the minimum inhibitory concentration value of the positive control (naystatin) was 10 *μ*g/ml. And the half maximal inhibitory concentration (IC_50_) value (21.23 *μ*g/ml) of **1** against *Exserohilum turcicum* was determined by analyzing its inhibition effect on the mycelial growth, using cycloheximide (IC_50_ = 46.70 *μ*g/ml) as the positive control.

## Introduction

*Candida albicans*, as an opportunistic pathogenic fungus, normally maintain symbiosis with the human body in the skin, oral cavity, and gastrointestinal tract (Mishra and Koh, 2021). When the body’s homeostasis is destroyed, *C. albicans* transforms into pathogenic fungi, causing various fungal diseases from superficial skin infections to life-threatening systemic infections (Pappas et al., 2009; Hall and Noverr, 2017). According to statistics, four hundred thousand people are infected with *C, albicans* every year, and 75% of women suffer from vulvovaginal candidiasis at least once in their lives (Fidel et al., 2004; Yang et al., 2014; Rajendran et al., 2016). Even with drug treatment, the fatality rate of invasive *C. albicans* infection is still close to 40% (Lohse et al., 2018; Whitesell et al., 2019). Among immunocompromised people such as chemotherapy and organ transplantation, the mortality rate of fungal diseases caused by *C. albicans* is 33–50% (Krcmery et al., 2000; Winston, 1999; Levesque et al., 2015). Therefore, infections caused by *C. albicans* are still nonnegligible threats to human health.

**FIGURE 1:**
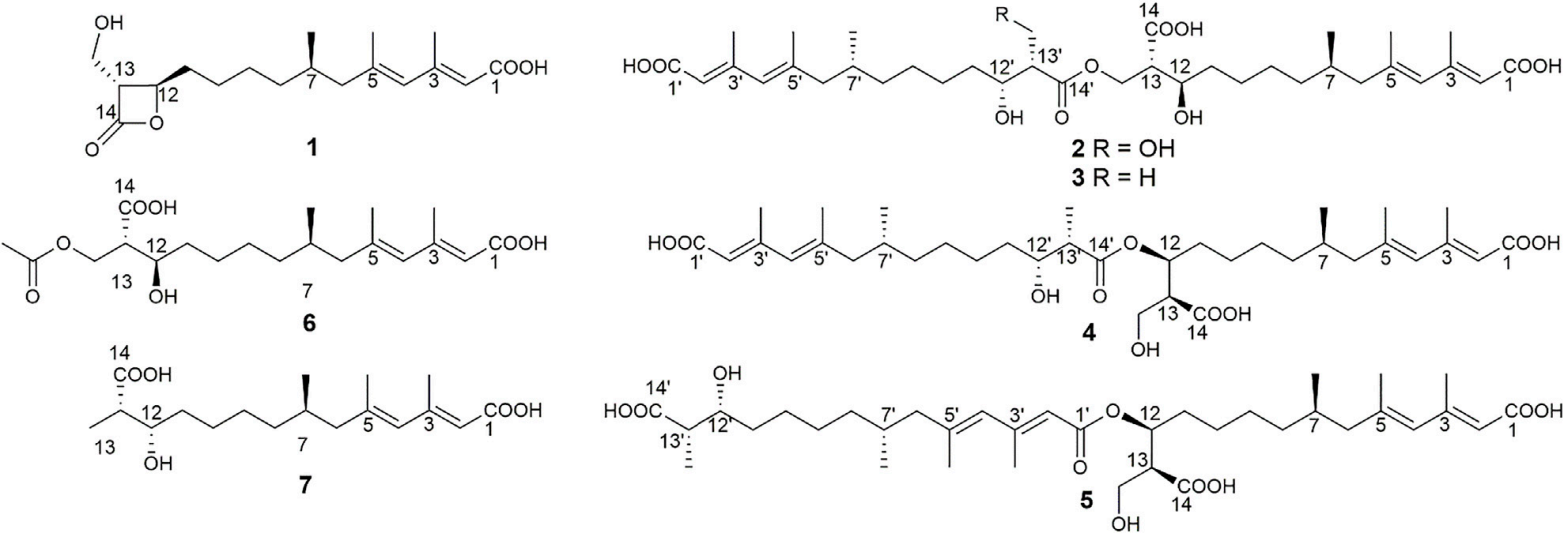
Chemical structures of compounds **1**‒**7**.

As an antifungal agent, fluconazole is widely used in the treatment of fungal diseases caused by *C. albicans* because of its low price, low toxicity, and high efficiency (Lu et al., 2017). However, the drug resistance of *C. albicans* caused by the widespread use of fluconazole is becoming an increasingly serious problem, and the discovery for new antifungal drugs has become more and more urgent (Whaley et al., 2016; Lu et al., 2017; Lu et al., 2021). Fungal secondary metabolites, as an important source of antifungal drugs (Baker et al., 2007; Di Santo, 2010; Cantrell et al., 2012; Schueffler and Anke, 2014), have attracted much more attention from the researchers. In the past 10 years, 25% of antifungal active compounds are derived from fungi (Aldholmi et al., 2019). Among all the fungal microbial resources, plant endophytic fungi were thought as the valuable resources for the discovery of antifungal agents (Uzma et al., 2018; Newman and Cragg, 2020). Recently, a program to discover antifungal constituents from endophytic fungi associated with characteristic food resources of Yunnan Province, China, was conducted in our lab. Accordingly, an antifungal screening of the strain fermentation extracts against *C. albicans* targeted an endophytic fungus from stems of tea trees, *Scopulariopsis candelabrum* KIB-int20. Secondary metabolites reported from the genus *Scopulariopsis* were mainly cyclodepsipeptides (such as scopularides A and B) and some dihydroquinolin-2-one-containing alkaloids (Yu et al., 2008; Shao et al., 2015; Elbanna et al., 2019). In this study, seven mono-/bis-alkenoic acid derivatives (**1–7**, Figure 1) were isolated from *S. candelabrum* KIB-int20 during a screening for antifungal secondary metabolites. We herein report the isolation, structure elucidation and antifungal activity of these polyketides.

## Materials and Methods

### General Experimental Procedures

Optical rotations were measured on an Autopol VI manufactured by Rudolph Research Analytical, Hackettstown, NJ, United States IR spectra were measured on a Nicolet iS10 FT-IR spectrometer (Thermo Fisher Scientific, United States) with KBr disks. NMR spectra were recorded in CDCl_3_ (*δ*_H_ 7.26 ppm, *δ*_C_ 77.16 ppm) or DMSO-*d*_6_ (*δ*_H_ 2.50 ppm, *δ*_C_ 39.52 ppm) using Bruker Avance III 600 or 800 MHz spectrometers (Bruker Corp, Switzerland). HR-ESI-MS analysis were carried out on a Shimadzu UPLC-IT-TOF mass spectrometer (Shimadzu Corp, Japan). Silica gel (100–200 mesh and 200–300 mesh, Qingdao Marine Chemical Inc, China) and Sephadex LH-20 (18–111 *μ*m, Pharmacia Biotech Ltd, Sweden) were used for the chromatography column (CC). Precoated silica gel GF_254_ plates (0.20–0.25 mm in thickness, Qingdao Marine Chemical Inc, China) were used for thin-layer chromatography (TLC) analyses. Semipreparative HPLC was conducted on a Hitachi Chromaster system (Hitachi Ltd, Japan), equipped with a DAD detector and a YMC-Triart C_18_ column (250 × 10.0 mm i. d, 5 *μ*m), using a flow rate of 3.0 ml/min at a column temperature at 28°C, and 0.1% (v/v) acetic acid was added to each HPLC mobile phase.

### Strain Isolation and Cultivation

Strain *S. candelabrum* KIB-int20 was isolated from the stems of tea trees (*Camellia sinensis* (L.) O. Ktze) from Dali, Yunnan Province, China. It was identified as *S. candelabrum* by a combination of ITS sequence and fungal morphological identification. The internal transcribed spaces (ITS) region was amplified and sequenced using the general primers ITS1 (5′-TCC​GTA​GGT​GAA​CCT​GCG​G-3′) and ITS4 (5′-TCC​TCC​GCT​TAT​TGA​TAT​GC-3′). The ITS region of the fungus was a 605 bp DNA sequence (GenBank No. OK445701), which showed 99% identity to the ITS sequence of strain *S. candelabrum* (GenBank No. LM652483.1).

*S. candelabrum* KIB-int20 was first inoculated on a PDA (filtrate of boiled fresh potatoes 200 g/L, dextrose 20 g/L, agar 20 g/L) plate for 5 days, and then transferred to several PDA plates for another 7 days culture. About one-sixth of agar blocks with fungi mycelium was inoculated into a tissue culture vessel (370 ml) containing the fermentation medium. For each tissue culture vessel, 20 g cargo rice, 10 g peptone and 12 ml water were added and sterilized at 121°C for 30 min immediately. The inoculated medium was statically cultivated for 1 month in a dark environment at room temperature. Strain *S. candelabrum* KIB-int20 was finally fermented with 5 kg of cargo rice in total.

### Extraction and Isolation

The fermentation solid of *S. candelabrum* KIB-int20 was extracted with acetone (10 L×2, d×2) at room temperature. The extracts were concentrated to remove organic solvent. The aqueous residue was then partitioned with EtOAc (2.5 L×4) to obtain an oily crude extract (50 g). The extract was then subjected to silica gel CC eluting with petroleum ether−EtOAc (1:0, 10:1, 5:1, 2:1, 1:1, 1:2, 1:5, 1:10 and 0:1, v/v) to give nine fractions (A−I). An antifungal screening of each fraction against *C. albicans* was conducted, and fraction D turned out to be active. The main metabolites in each fraction were further analyzed by DAD-HPLC. Main metabolites in fractions D and E shared the same UV absorptions. In this way, fractions D and E were selected for further study. Selected fraction E (petroleum ether−EtOAc 1:1) was first separated by Sephadex LH-20 CC (CH_2_Cl_2_−CH_3_OH, 1:1), and divide it into six subfractions according to the detection results of thin layer chromatography (10% ethanol sulfate in EtOH was served as chromogenic agent). Further purification of these subfractions by semipreparative DAD-HPLC gave compounds **2** (78% methanol in H_2_O, *t*_R_ = 19.8 min, 3.5 mg), **3** (78% methanol in H_2_O, *t*_R_ = 33.0 min, 3.5 mg), **4** (78% methanol in H_2_O, *t*_R_ = 38.0 min, 6.4 mg), and **5** (78% methanol in H_2_O, *t*_R_ = 50.0 min, 5.4 mg). Another selected fraction D (petroleum ether−EtOAc, 2:1) was sequentially subjected to Sephadex LH-20 CC (CH_2_Cl_2_−CH_3_OH, 1:1) and semipreparative DAD-HPLC to afford **1** (65% methanol in H_2_O, *t*_R_ = 32.5 min, 8.2 mg), **6** (65% methanol in H_2_O, *t*_R_ = 34 min, 6.9 mg), and **7** (65% methanol in H_2_O, *t*_R_ = 42.0 min, 4.4 mg).

Fusariumester C (**2**): colorless oil [α]^19.9^_D_ 4.5 (*c* 0.2, MeOH) UV (MeOH); λ_max_ (log ε) 196 (3.99), 232 (3.59), 269 (3.93) nm; IR (KBr) ν_max_ 3,419, 2,927, 2,856, 1712, 1,620, 1,382, 1,250, 1,176 cm^₋1^. HR-ESI-MS: *m/z* 665.3909 [M ‒ H]^‒^ (calcd for C_36_H_57_O_11_, 665.3906). ^1^H NMR (600 MHz, DMSO-*d*_6_) data see Table 1, and ^13^C NMR (150 MHz, DMSO-*d*_6_) data see Table 2.

**TABLE 1:** ^1^H NMR Data of Compounds **2**–**7** (*δ* in ppm, *J* in Hz).

No	2*^b^*	3*^b^*	4*^c^*	5*^b^*	6*^b^*	7*^d^*
2	5.63 s	5.60 s	5.66 s	5.60 s	5.59 s	5.69 s
4	5.71 s	5.71 s	5.69 s	5.69 s	5.71 s	5.73 s
6a	2.02*^a^*	2.03*^a^*	2.06 dd (13.1, 6.0)	2.01*^a^*	2.02 dd (13.0, 6.5)	2.05 dd (13.2, 6.6)
6b	1.80*^a^*	1.82*^a^*	1.83*^a^*	1.80*^a^*	1.82 dd (13.0, 8.0)	1.88 dd (13.2, 7.7)
7	1.61*^a^*	1.61*^a^*	1.60*^a^*	1.59*^a^*	1.62 m	1.66 m
8a	1.23*^a^*	1.24*^a^*	1.25*^a^*	1.05*^a^*	1.25*^a^*	1.31*^a^*
8b	1.03*^a^*	1.05*^a^*	1.07*^a^*	0.98*^a^*	1.03*^a^*	1.10*^a^*
9a	1.33*^a^*	1.33*^a^*	1.30*^a^*	1.21*^a^*	1.25*^a^*	1.34*^a^*
9b	1.23*^a^*	1.23*^a^*	1.19*^a^*	1.32*^a^*	1.34*^a^*	1.42*^a^*
10a	1.32*^a^*	1.32*^a^*	1.28*^a^*	1.22*^a^*	1.36*^a^*	1.48*^a^*
10b	1.24*^a^*	1.24*^a^*	1.17*^a^*	1.19*^a^*	1.26*^a^*	1.37*^a^*
11a	1.34*^a^*	1.29*^a^*	1.54*^a^*	1.53*^a^*	1.35*^a^*	1.51*^a^*
11b	1.25*^a^*	1.24*^a^*	1.50*^a^*		1.25*^a^*	1.47*^a^*
12	3.57*^a^*	3.57*^a^*	4.99 m	5.03 td (8.3, 3.7)	3.63 m	3.69 m
13	2.45*^a^*	2.47 *^a^*	2.55*^a^*	2.58 m	2.57 m	2.56 m
3-CH_3_	2.14 s	2.14 s	2.13 s	2.12 s	2.14 s	2.24 s
5-CH_3_	1.73 s	1.73 s	1.74 s	1.72 s	1.74 s	1.81 s
7-CH_3_	0.78*^a^*	0.78 d (6.5)	0.79 d (6.5)	0.77 d (7.0)	0.79 d (6.6)	0.84 d (6.6)
13-CH_2_OR or 13-CH_3_	4.13*^a^*	4.14 m	3.53*^a^*	3.54*^a^*	4.11^a^	1.26 d (7.2)
	4.18 dd (10.6, 5.8)	4.29 dd (10.8, 4.7)		3.48*^a^*	4.15 dd (10.7, 5.3)	
2′	5.56 s	5.56 s	5.57 s	5.58 s		
4′	5.69 s	5.69 s	5.73 s	5.75 s		
6′a	2.02*^a^*	2.03*^a^*	2.06 dd (13.1, 6.0)	2.01*^a^*		
6′b	1.80*^a^*	1.82*^a^*	1.83*^a^*	1.80*^a^*		
7′	1.61*^a^*	1.61*^a^*	1.62*^a^*	1.59*^a^*		
8′a	1.23*^a^*	1.24*^a^*	1.25*^a^*	1.22*^a^*		
8′b	1.03*^a^*	1.05*^a^*	1.07*^a^*	1.05*^a^*		
9′a	1.33*^a^*	1.33*^a^*	1.25*^a^*	1.21*^a^*		
9′b	1.23*^a^*	1.23*^a^*	1.19*^a^*	1.32*^a^*		
10′a	1.32*^a^*	1.32*^a^*	1.39*^a^*	1.22*^a^*		
10′b	1.24*^a^*	1.24*^a^*	1.26*^a^*	1.19*^a^*		
11′a	1.34*^a^*	1.38*^a^*	1.31*^a^*	1.31*^a^*		
11′b	1.25*^a^*	1.30*^a^*	1.26*^a^*	1.23*^a^*		
12′	3.57*^a^*	3.56*^a^*	3.59*^a^*	3.55*^a^*		
13′	2.44*^a^*	2.39 m	2.44 m	2.32 m		
3′-CH_3_	2.12 s	2.12 s	2.16 s	2.17 s		
5′-CH_3_	1.75 s	1.75 s	1.76 s	1.76 s		
7′-CH_3_	0.79*^a^*	0.79 d (6.5)	0.80 d (6.5)	0.78 d (7.0)		
13′-CH_2_OH or 13′-CH_3_	3.54*^a^*	0.95 d (7.0)	0.99 d (7.0)	0.95 d (7.0)		
	3.49*^a^*					
Ac-CH_3_					1.96 s	

a Overlapped signals.

b Recorded at 600 MHz, in DMSO-d_6_.

c Recorded at 800 MHz, in DMSO-d_6._

d Recorded at 600 MHz, in CDCl_3._

**TABLE 2:** ^13^C NMR Data of Compounds **2**–**7** (*δ* in ppm).

No	2*^a^*	3*^a^*	4*^b^*	5*^a^*	6*^a^*	7*^c^*
1	167.8	167.9	168.4	167.8	167.7	171.9
2	118.6	118.6	119.4	118.6	118.3	116.4
3	152.6	152.6	153.0	151.9	152.4	157.5
4	129.3	129.3	129.8	129.2	129.2	129.4
5	140.8	140.5	140.6	140.5	140.7	142.5
6	48.6	48.5	48.9	48.3	48.5	49.1
7	30.3	30.2	30.6	30.0	30.1	30.8
8	36.4	36.3	36.8	35.6	35.9	36.4
9	26.4	26.4	26.3	26.4	26.1	26.8
10	25.3	25.2	25.0	24.8	25.4	25.6
11	34.5	32.9	32.0	31.7	34.2	34.6
12	68.8	69.0	71.7	70.6	68.8	73.2
13	50.9	51.7	53.3	52.7	50.9	45.1
14	173.8*^d^*	173.9	174.3	173.7	173.2	180.7
3-CH_3_	19.1	19.1	19.5	19.1	19.1	20.0
5-CH_3_	18.1	18.1	18.5	18.1	18.1	18.6
7-CH_3_	19.2	19.3	19.7	19.3	19.3	19.6
13-CH_2_OR or 13-CH_3_	62.6	62.9	60.4	59.8	62.5	14.2
1′	167.7	167.7	168.2	165.7		
2′	118.2	118.2	118.7	116.9		
3′	152.6	152.6	153.0	154.1		
4′	129.2	129.2	129.6	129.0		
5′	140.8	140.9	141.2	141.9		
6′	48.4	48.4	48.9	48.6		
7′	30.3	30.3	30.8	30.3		
8′	36.4	36.3	36.8	36.3		
9′	26.4	26.2	26.9	25.7		
10′	25.5	25.4	26.0	25.4		
11′	34.7	34.8	33.4	33.1		
12′	68.9	71.3	71.7	71.4		
13′	55.2	45.9	46.8	45.9		
14′	172.4	174.2	174.3	176.3		
3′-CH_3_	19.1	19.1	19.5	19.3		
5′-CH_3_	18.1	18.1	18.6	18.2		
7′-CH_3_	19.2	19.4	19.9	19.4		
13′-CH_2_OR or 13′-CH_3_	59.4	12.2	12.8	12.6		
Ac-CO					170.3	
Ac-CH_3_					20.7	

a Recorded at 150 MHz, in DMSO-d_6_.

b Recorded at 200 MHz, in DMSO-d_6_.

c Recorded at 150 MHz, in CDCl_3_.

d Signals were not detected in ^13^C NMR, but were found in an HMBC spectrum.

Fusariumester D (**3**): colorless oil [α]^19.9^_D_ 3.2 (*c* 0.2, MeOH) UV (MeOH); λ_max_ (log ε) 196 (4.07), 232 (3.69), 270 (4.07) nm; IR (KBr) ν_max_ 3,659, 3,433, 2,927, 2,857, 2,011, 1,711, 1,621, 1,530, 1,378, 1,343, 1,324, 1,251, 1,176 cm^₋1^. HR-ESI-MS: *m/z* 649.3952 [M‒H]^‒^ (calcd for C_36_H_57_O_10_, 649.3957). ^1^H NMR (600 MHz, DMSO-*d*_6_) data see Table 1, and ^13^C NMR (150 MHz, DMSO-*d*_6_) data see Table 2.

Fusariumester E (**4**): colorless oil [α]^19.9^_D_ 1.0 (*c* 0.2, MeOH) UV (MeOH); λ_max_ (log ε) 196 (4.22), 229 (3.86), 269 (4.32) nm; IR (KBr) ν_max_ 2,925, 2,854, 2,644, 2,566, 1,687, 1,604, 1,381, 1,325, 1,253, 1,178 cm^₋1^. HR-ESI-MS: *m/z* 649.3959 [M‒H]^‒^ (calcd for C_36_H_57_O_10_, 649.3957). ^1^H NMR (800 MHz, DMSO-*d*_6_) data see Table 1, and ^13^C NMR (200 MHz, DMSO-*d*_6_) data see Table 2.

Fusariumester F (**5**): colorless oil [α]^19.9^_D_ ‒ 6.0 (*c* 0.2, MeOH) UV (MeOH); λ_max_ (log ε) 196 (4.35), 232 (3.96), 271 (4.38) nm; IR (KBr) ν_max_ 3,420, 2,927, 2,857, 2,644, 1,712, 1,619, 1,381, 1,234, 1,150 cm^₋1^. HR-ESI-MS: *m/z* 649.3956 [M‒H]^‒^ (cald for C_36_H_57_O_10_, 649.3957). ^1^H NMR (600 MHz, DMSO-*d*_6_) data see Table 1, and ^13^C NMR (150 MHz, DMSO-*d*_6_) data see Table 2.

Acetylfusaridioic acid A (**6**): colorless oil [α]^19.9^_D_ 5.7 (*c* 0.15, MeOH) UV (MeOH); λ_max_ (log ε) 196 (3.85), 230 (3.48), 270 (3.95) nm; IR (KBr) ν_max_ 3,412, 2,928, 2,859, 2,645, 1,740, 1,716, 1,618, 1,382, 1,250, 1,184 cm^₋1^. HR-ESI-MS: *m/z* 383.2074 [M‒H]^‒^ (calcd for C_20_H_31_O_7_, 383.2075). ^1^H NMR (600 MHz, DMSO-*d*_6_) data see Table 1, and ^13^C NMR (150 MHz, DMSO-*d*_6_) data see Table 2.

Fusaridioic acid D (**7**): colorless oil [α]^19.9^_D_ 7.2 (*c* 0.2, MeOH) UV (MeOH); λ_max_ (log ε) 196 (3.66), 230 (3.66), 269 (4.09) nm; IR (KBr) ν_max_ 3,400, 2,929, 2,858, 2,640, 2,229, 2,195, 2,179, 2,164, 2,153, 2,113, 2,056, 2,023, 2.011, 1,970, 1,959, 1,692, 1,622, 1,377, 1,324, 1,251, 1,175 cm^₋1^. HR-ESI-MS: *m/z* 325.2023 [M‒H]^‒^ (calcd for C_18_H_29_O_5_, 325.2020). ^1^H NMR (600 MHz, CDCl_3_) data see Table 1, and ^13^C NMR (150 MHz, CDCl_3_) data see Table 2.

### Antifungal Activity Assay

Rough Antifungal Activity Test: The rough antifungal activity of compounds **1**−**7** was measured by the filter paper agar diffusion method (Xu et al., 2015). 1 ml suspension (1 × 10^5^ CFU cell or spore concentration) of *C. albicans*, or *Exserohilum turcicum*, *Curvularia lunata*, or *Fusarium oxysporum* in 20% glycerin was inoculated in a Petri dish containing PDA medium; autoclaved paper disks (6 mm diameter) were placed around the fungal inoculant on the same Petri dish, and each of the paper disks impregnated with 10 *μ*g testing samples, nystatin (positive control) or an equivalent volume of methanol (blank control). Fungal inoculants were cultivated in dark at 30°C for 2 days, and then the size of the inhibition zones was analyzed. Each compound was retested three times.

Measurement of minimum inhibitory concentration (MIC) Values (Yu et al., 2016): A single colony of *C. albicans* on the SDA plate (1% peptone, 4% dextrose and 2% agar) was picked and inoculated into 5 ml YPD (1% yeast extract, 2% peptone and 2% dextrose) liquid medium and cultivated at 37°C, 200 r/min for 16 h to reach the logarithmic growth phase. According to the measured growth curve of *C. albicans*, the fungal inoculum was diluted with YPD liquid medium, ensuring the abundance of the strains was 3 × 10^7^ CFU/ml. Compound **1**, nystatin (positive control) and equivalent methanol (blank control) were dispensed in single wells and mixed with diluted fungal inoculum to make the final concentrations of tested compounds were 10, 20, 40, 80, 160 and 320 *μ*g/ml in a single well, respectively. After 48 h of shaking culture at 37°C and 200 r/min, the results were determined visually. The MIC was defined as the lowest concentration where there was no visible growth of *C. albicans*. All the experiments were carried out in triplicate.

Measurement of half maximal inhibitory concentration (IC_50_) Values (Weidenborner et al., 1990): The IC_50_ of compound **1** against *E. turcicum* was evaluated using 48-well culture plates. The conidia used in these experiments were collected from the 7-day-old culture of fungi grown on PDA. The conidia were collected and the suspension was diluted with sterile water and mix 1:1 with PDB (filtrate of boiled fresh potatoes 200 g/L, dextrose 20 g/L) solution for activity test. 1 ml 1/2 PDB spore suspension was added to single wells, and 1, 2, 4, 8, 16 or 32 *μ*L compound **1** or cycloheximide (10 mg/ml) was added to make the final concentration is 9.99–310.07 *μ*g/ml in a single well. After the 48-well plate was cultured at 200 r/min and 30°C for 7 days, the mycelium at each concentration were collected, dried and weighed. The inhibition rates were treated by nonlinear regression analysis of logistic dose–respond curves (Graph Pad Prism eight statistic software) to get the IC_50_ value.

## Results and Discussion

### Structural Elucidation

A series of chromatographic methods were used for the isolation of monomeric compounds from the strain fermentation extracts, and diverse spectroscopic analyses were used for their structure elucidation. As a result, seven polyketides were isolated and identified, including one known compound, hymeglusin (**1**) (Kumagai et al., 1992; Tomoda et al., 1988), four new bis-alkenoic acid derivatives named fusariumesters C−F (**2**–**5**), and two new alkenoic acid monomers named acetylfusaridioic acid A (**6**) and fusaridioic acid D (**7**). The absolute configuration of hymeglusin (**1**) was previously determined by chemical degradation method and Mosher method (Chiang et al., 1988). The optical rotation of hymeglusin (**1**) was {[α]^19.4^_D_ 24.56 (*c* 0.2, CHCl_3_)} in our project, which reported in the literature was [α]^22^_D_ 10.6 (*c* 0.1, CHCl_3_) (Kanaida et al., 2021).

**FIGURE 2:**
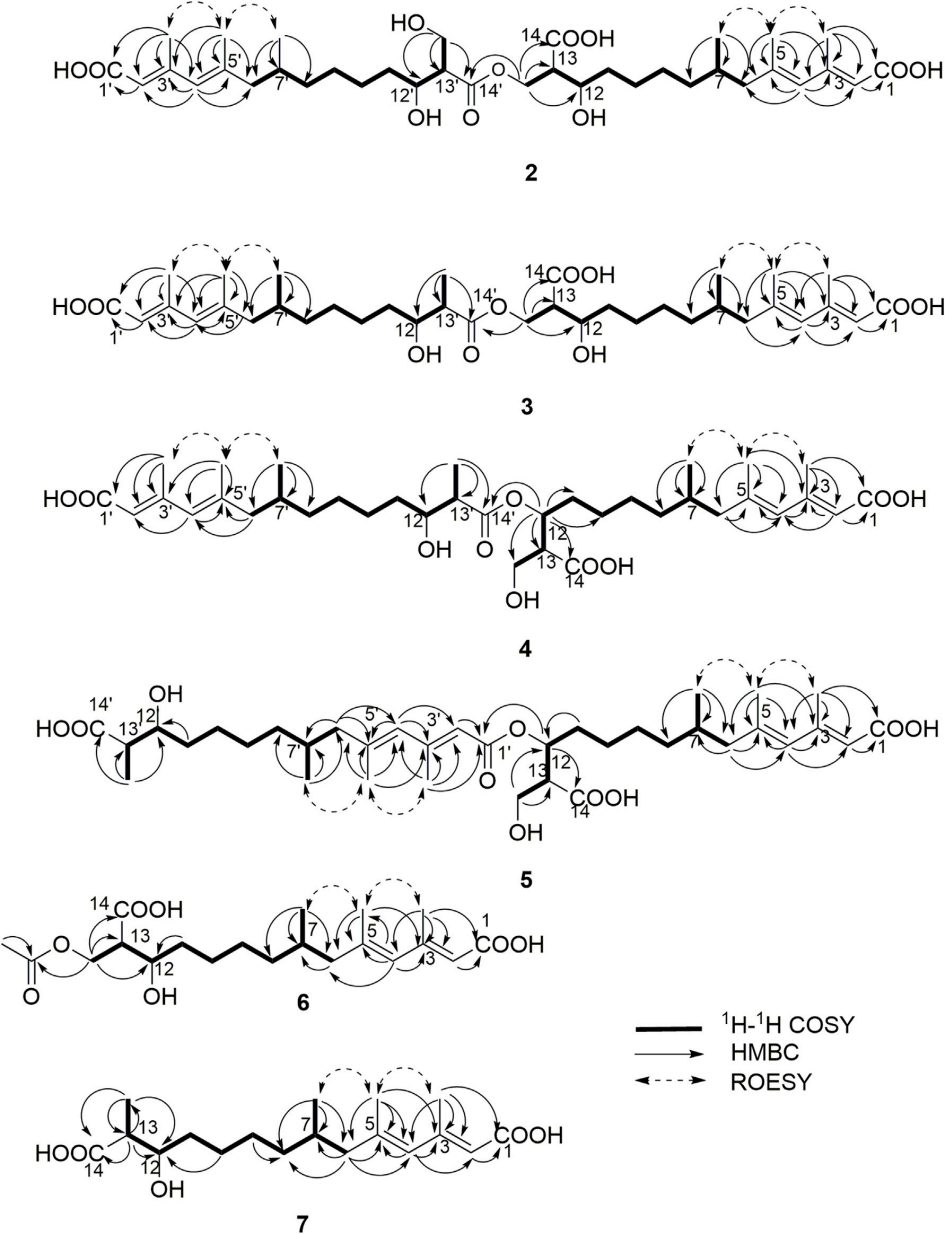
Key HMBC, ^1^H–^1^H COSY and ROESY correlations of compounds **2**‒**7**.

Compound **2** (fusariumester C) was isolated as colorless oil. Its molecular formula was determined to be C_36_H_58_O_11_ by HR-ESI-MS analysis (*m/z* 665.3909 [M ‒ H]^‒^, calcd for C_36_H_57_O_11_, 665.3906, Supplementary Figure S9), suggesting eight degrees of unsaturation. The IR spectrum of **2** displayed characteristic adsorptions for carbonyl groups and carbon-carbon double bonds at 1,712 and 1,620 cm^−1^, respectively. The ^13^C NMR data (Table 2) of **2** showed 36 carbon resonances, and all signals appeared in pairs. Moreover, each pair of the signals closely resembled those of fusaridioic acid A (Liu et al., 2018). Additionally, comparing the molecular weight (666 Da) of **2** with twice that of fusaridioic acid A (342 Da) yielded a difference of 18 Da. Accordingly, **2** was speculated to be an esterified dimer of fusaridioic acid A (Liu et al., 2018). Further detailed analysis of its NMR data (Supplementary Figures S3–8) supported this hypothesis. Based on the literature report (Liu et al., 2018), two ^13^C NMR signals at *δ*_C_ 167.7 and *δ*_C_ 167.8 were obviously assigned as carboxylic acid carbonyls connecting to quaternary olefinic carbons, while signals at *δ*_C_ 173.8 and *δ*_C_ 172.4 were assigned as aliphatic carboxylic acid carbonyl groups or ester carbonyl groups. The methines at *δ*_C_ 68.8 and *δ*_C_ 68.9 were attributed to hydroxy-substituted ones. The two methylenes at *δ*_C_ 62.6 and *δ*_C_ 59.4 were assigned to be oxygen-bearing ones, and the small difference between their chemical shifts may due to the formation of an ester bond for one of these two carbons. The ^1^H NMR data (Table 1 and Supplementary Figure S3) of **2** revealed four olefinic proton signals at *δ*_H_ 5.56, 5.63, 5.71, 5.69. Detailed HMBC correlations associated with these four above-mentioned protons established the presence of two pairs of diene moieties conjugating with terminal carboxyls (the fragments from C-1 to C-6, and from C-1′ to C-6′, Figure 2). The ^1^H NMR data of **2** also showed two overlapped doublets (*δ*_H_ 0.78 and 0.79) of methyls in the high field region (Supplementary Figure S3). Starting with these two above-mentioned methyl signals, two similar aliphatic carbon chains [7-Me(C-6)/C-7/C-8/C-9/C-10/C-11/C-12/C-13/13-CH_2_O and 7′-Me(C-6′)/C-7′/C-8′/C-9′/C-10′/C-11′/C-12′/C-13′/13′-CH_2_O, Figure 2] were deduced based on a combined analyses of its HSQC and ^1^H–^1^H COSY spectra. Lastly, the key HMBC correlations from the proton at *δ*_H_ 4.13 (one proton of 13-CH_2_OR) to the carbons of C-12, C-13, C-14 and C-14′ demonstrated that two molecules of fusaridioic acid A were dimerized via the ester bond built by 13-CH_2_OH and the carboxylic acid group at C-14′ (Figure 2). Thus, the planar structure of **2** was elucidated.

The configurations of the four double bonds in **2** were revealed by the analysis of its ROESY spectrum (Figure 2 and Supplementary Figure S8). However, the ^1^H NMR data of **2** were helpless for the determination of its stereochemistry because of signal overlapping. For the stereochemistry of reported mono-/bis-alkenoic acid derivatives, the configurations of C-7 (7′) and C-13 (13′) were conserved to be 7*R* (7′*R*) and 13*S* (13′*S*) (Liu et al., 2018; Niu et al., 2019; Tang et al., 2019), while the configuration of C-12 (12′) turned out to be 12 (12′) *R* or 12 (12′) *S* (Liu et al., 2018). Recently, it was reported that the configurations of fungal polyketides were conserved in general with few exceptions (Takino et al., 2021). Therefore, in view of the shared biosynthetic pathway of mono-/bis-alkenoic acid derivatives, as well as previous literature reports (Liu et al., 2018; Niu et al., 2019; Tang et al., 2019), the configurations of C-7 (7′) and C-13 (13′) in **2** were supposed to be 7*R* (7′*R*) and 13*S* (13′*S*), respectively. Compared with the ^13^C NMR and ^1^H NMR chemical shifts of C-12 (12′) in the dimers reported in the literature (Liu et al., 2018, Supplementary Table S1), the absolute configurations of C-12 (12′) in compound **2** were determined to be 12*R* (12′*R*). In this way, the chemical structure of **2** was identified as shown in Figure 1. Since three similar alkenoic acid dimers were given the trivial names of fusariumesters A_1_, A_2_, and B (Liu et al., 2018) previously, compound **2** was named as fusariumester C.

Compound **3** (fusariumester D**)** was isolated as colorless oil. Its molecular formula was confirmed to be C_36_H_58_O_10_ by HR-ESI-MS data (*m/z* 649.3952 [M ‒ H]^‒^, calcd for C_36_H_57_O_10_, 649.3957, Supplementary Figure S16), indicating eight degrees of unsaturation. The molecular weight of compound **3** (650 Da) was lower than that of compound **2** (666 Da) with a difference of 16 Da. The ^1^H and ^13^C NMR spectra of **3** closely resembled those of **2** (Table 1 and Table 2), and the comparison of their ^1^H and ^13^C NMR data (Supplementary Figures S10,11) indicated a methyl (13′-Me) in **3** instead of a hydroxymethyl (13′-CH_2_OH) in **2**. This hypothesis was confirmed by HMBC (Supplementary Figure S13) correlations from protons of 13′-CH_2_OH to C-12′, C-13′, and C-14’ (Figure 2). Analogously, a group of HMBC correlations from the proton at *δ*_H_ 4.14 (one proton of 13-CH_2_OR) to C-12, C-13, C-14, and C-14′ proved that **3** shared the same esterified dimerization way as **2**.

Compound **4** (fusariumester E) is a colorless viscous oil. Its molecular formula was determined to be same with that of **3** based on the HR-ESI-MS analysis (*m/z* 649.3959 [M ‒ H]^‒^, calcd for C_36_H_57_O_10_, 649.3957, Supplementary Figure S23). The ^1^H and ^13^C NMR data of **4** and **3** were highly similar (Table 1 and Table 2). The most striking differences between their ^13^C NMR data were that chemical shift of 13-CH_2_OR was upfield shifted from 62.9 ppm in **3** to 60.4 ppm in **4**, and the chemical shift of C-12 was downfield shifted from 69.0 ppm in **3** to 71.7 ppm in **4**. In view of the same molecular formula shared by **3** and **4**, this phenomenon implied that **4** might possess a different dimerization site. Key HMBC correlations from H-12 to C-10, C-11, C-13, C-14 and C-14′ certified that an ester bond was built in **4** with the carboxylic acid group in one monomer at C-14′ and the hydroxyl at C-12 in another monomer (Figure 2).

Compound **5** (fusariumester F) was also isolated as colorless oil. Its molecular formula was speculated to be C_36_H_58_O_10_ by HR-ESI-MS analysis (*m/z* 649.3956 [M ‒ H]^‒^, cald for C_36_H_57_O_10_, 649.3957, Supplementary Figure S30). Comparing the ^1^H and ^13^C NMR data of **5** with those of **2–4** (Table 1 and Table 2) predicted **5** also to be a bis-alkenoic acid derivative but decorating with another new esterified dimerization way. A vital HMBC (Supplementary Figure S27) cross peak of H-12/C-1′ defined that the hydroxyl at C-12 in one monomer and the terminal carboxylic acid at C-1′ contributed to the dimerized ester bond in **5**.

Compound **6** (acetylfusaridioic acid A) was isolated as colorless oil. Its elemental composition was determined to be C_20_H_32_O_7_ by HR-ESI-MS analysis (*m/z* 383.2074 [M ‒ H]^‒^, calcd for 383.2075 C_20_H_31_O_7_, Supplementary Figure S37), indicating five degrees of unsaturation. Its ^1^H NMR and HSQC data distinctly showed two olefinic protons (*δ*_H_ 5.59 and 5.71), two oxygenated *gem*-protons (*δ*_H_ 4.11 and 4.15), and four methyl groups (three singlets at *δ*_H_ 2.14, 1.96, 1.74, and one doublet at *δ*_H_ 0.79). Three down-field shifted carbonyls (*δ*_C_ 173.2, 170.3, 167.7), four olefinic carbons (*δ*_C_ 152.4, 140.7, 129.2, 118.3), one oxygen-bearing methylene (*δ*_C_ 62.5), and four methyl groups (*δ*_C_ 20.7, 19.3, 18.1, 19.1) were observed in the ^13^C NMR spectrum of **6**. Examination of its detailed ^13^C NMR data with those of fusaridioic acid A (Liu et al., 2018) suggested that **6** was the product of acetylation at 13-CH_2_OH in fusaridioic acid A. Key HMBC (Supplementary Figure S34) correlations from protons (*δ*_H_ 4.11 and 4.15) of 13-CH_2_O moiety to the carbonyl (*δ*_C_ 170.3) of the acetyl group supported this deduction (Figure 2). Like compound **2**, the configurations of C-7 and C-13 in **6** were supposed to be 7*R* and 13*S* based on a thought of conserved biosynthetic logic. And the C-12 (12′) absolute configurations in compounds **3**, **4**, **5**, and **6** were determined to be 12*R* (12′*R*), 12*S* (12′*R*), 12*S* (12′*R*), and 12*R*, respectively, by comparisons of their proton NMR data of H-12 (12′) with literature reports (Supplementary Table S1).

Fusaridioic acid D **(7)** was found to possess the molecular formula C_18_H_30_O_5_ from the HR-ESI-MS data (*m/z* 325.2023 [M‒H]^‒^, calcd for 325.2020 C_18_H_29_O_5_, Supplementary Figure S44), corresponding to an unsaturation index of four. Detailed analyses of its ^1^H and ^13^C NMR data (Table 1 and Table 2) revealed that **7** is one of the monomers involved in compound **3**. That’s to say the chemical structure of **7** is a dehydroxylation product of fusaridioic acid A (Liu et al., 2018). This hypothesis was further proved by key HMBC (Supplementary Figure S41) correlations from a methyl (*δ*_H_ 1.26) to C-14, C-13, and C-12 in **7** (Figure 2). By referring to the ^13^C NMR and ^1^H NMR chemical shifts of known compounds with similar structural units, the absolute configuration of C-12 in compound **7** was determined to be 12*S* (Supplementary Tables S1) (Ying and Hong, 2007; Bisek et al., 2008).

### Evaluation of Antifungal Activity

The rough antifungal activity of compounds **1–7** were measured by the filter paper agar diffusion method (Xu et al., 2015). Compounds **2–7** showed no significant inhibitory activity against *C. albicans*, and only compound **1** could inhibit *C. albicans* (Supplementary Figure S49). The MIC value (20 *μ*g/ml) of **1** against *C. albicans* was then determined by broth microdilution techniques (Yu et al., 2016), using nystatin (MIC = 10 *μ*g/ml) as the positive control (Supplementary Figure S46). As the previous references reported the antifungal activity of alkenoic acids against plant pathogens (Liu et al., 2018; Niu et al., 2019; Tang et al., 2019), all the isolates were also submitted to an antifungal assay against three agricultural pathogenic fungi, *Exserohilum turcicum*, *Curvularia lunata*, and *Fusarium oxysporum*. As a result, only compound **1** showed a good inhibitory activity against *E. turcicum*, with an IC_50_ value (21.23 *μ*g/ml) significantly lower than the positive control, cycloheximide (IC_50_ = 46.70 *μ*g/ml) (Supplementary Figures S47,48). Except for hymeglusin (**1**), none of the isolated alkenoic acid derivatives could suppress the growth of tested pathogenic fungi. Therefore, it could be concluded that the *β*-lactone ring was a key moiety for the antifungal activity. Just as the previous reports, the antifungal activity of alkenoic acid derivatives is always accompanied by the appearance of the *β*-lactone ring (Liu et al., 2018; Niu et al., 2019; Tang et al., 2019). It was reported that the *β*-lactone ring played a key role in inhibiting fungal HMG-CoA (3-hydroxy-3-methylglutaryl-CoA) synthase activity (Greenspan et al., 1993; Tomoda et al., 2004; Skaff et al., 2012).

## Conclusions

In recent years, 23 alkenoic acid derivatives were reported in total (Supplementary Figure S51) (Liu et al., 2018; Niu et al., 2019; Tang et al., 2019). Among them, most of the alkenoic acid derivatives are acyclic, and seven of them are decorating with terminal lactone rings, including *β*-lactone (such as fusarilactone A, fusarilactone B, hymeglusin, fusarisolin A, and fusariumester B), *γ*-lactone (such as fusarisolin B), and *δ*-lactone (such as fusarilactone C). For these reported alkenoic acids, oxidative modifications (carboxylic acids or hydroxyls) often occur at C-1, C-12, Me-13, and C-14. The stereochemistry of acyclic alkenoic acids is usually conserved with 7*R* and 13*S*, while the stereochemistry of C-12 with a hydroxy modification is hybrid with *R* (eg, fusarisolin D, fusaridioic acid A, and fusariumester A_2_) or *S* (eg, fusariumester A_1_ and L-660282) configuration. Preliminary biosynthetic study of 1233A (equal to F-244, L-659, 699, or hymeglusin) revealed that alkenoic acids are built via a type I polyketide synthase (PKS) logic (Kumagai et al., 1992; Kato et al., 2020). A plausible biosynthetic pathway of compounds **1**–**7** was also proposed in our project (Supplementary Figure S50).

To date, only three examples of dimerized alkenoic acid compounds (fusariumesters A_1_, A_2_, and B) have been reported (Liu et al., 2018), of which fusariumesters A_1_ and A_2_ are dimerized by an ester bond formed by the hydroxyl group at C-12 and the carboxyl group at C-14′, and fusariumester B is dimerized by an ester bond involving the hydroxyl group at C-12 and the carboxyl group at C-1′. In this paper, we reported six new mono-/bis-alkenoic acid derivatives (**2**–**7**) and one known alkenoic acid derivative (**1**) from an endophytic fungi *S. candelabrum*. Consistent with previous reports (Greenspan et al., 1987; Omura et al., 1987; Tomoda et al., 1988), the antifungal screening found that hymeglusin (**1**) with a *β*-lactone ring exhibited obvious activity against *C. albicans* (Tomoda et al., 1988) and *E. turcicum*. In addition, our discovery of these four new dimerized alkenoic acids (fusariumesters C−F, **2**–**5**) expanded the structure diversity of this family of natural products. These alkenoic acid dimers may be formed via the esterification of the same or different monomers with the aid of one or more esterases (Xu et al., 2020). It is also possible that the dimerization is initiated by thioesterase catalysis (Du and Lou, 2010).

## Data Availability

All datasets for this study are included in the article/Supplementary Material.
