# Supplementary material for: Mono-/Bis-Alkenoic Acid Derivatives From an Endophytic Fungus Scopulariopsis candelabrum and Their Antifungal Activity
Source: Front Chem. 2022 Jan 11;9:812564. doi: 10.3389/fchem.2021.812564 (PMC8787343; doi:10.3389/fchem.2021.812564)
Supplement: Supplementary file 1 [file DataSheet1.pdf]

frontiers

Supplementary Material

Mono-/Bis-Alkenoic Acid Derivatives from an Endophytic Fungus *Scopulariopsis candelabrum* and Their Antifungal Activity

Jun Tang [†, 1, 4], Xueshuang Huang [†, 2], Ming-Hang Cao [1], Zhiyan Wang [1, 4], Zhiyin Yu [3], Yijun Yan [1], Jian-Ping Huang [3], Li Wang [1, 3, *] and Sheng-Xiong Huang [1, 2,*]

[1] State Key Laboratory of Phytochemistry and Plant Resources in West China, CAS Center for Excellence in Molecular Plant Sciences, Kunming Institute of Botany, Chinese Academy of Sciences, Kunming 650201, China

[2] Hunan Provincial Key Laboratory for Synthetic Biology of Traditional Chinese Medicine, Hunan University of Medicine, Huaihua 418000, China

[3] State Key Laboratory of Southwestern Chinese Medicine Resources, Innovative Institute of Chinese Medicine and Pharmacy, Chengdu University of Traditional Chinese Medicine, Chengdu 611137, China

[4]Savaid Medical School, University of Chinese Academy of Sciences, Beijing 100049, China

[†] These authors contributed equally to this work.

*** Correspondence**:
Prof. Dr. Sheng-Xiong Huang, E-mail: sxhuang@mail.kib.ac.cn;
Dr. Li Wang, E-mail: liwangcyy@cdutcm.edu.cn

Table S1. Comparisons of NMR data of carbons with secondary alcohols between references and our project. 1
Figure S1. ^{13}C-NMR ($CDCl_3$, 150 MHz) of compound **1**. 3
Figure S2. ^{1}H-NMR ($CDCl_3$, 600 MHz) of compound **1**. 4
Figure S3. ^{1}H-NMR (DMSO-d_6, 600 MHz) of compound **2**. 5
Figure S4. ^{13}C-NMR (DMSO-d_6, 150 MHz) of compound **2**. 6
Figure S5. HSQC (DMSO-d_6, 600 MHz) of Compound **2**. 7
Figure S6. HMBC (DMSO-d_6, 600 MHz) of compound **2**. 8
Figure S7. COSY (DMSO-d_6, 600 MHz) of compound **2**. 9
Figure S8. ROESY (DMSO-d_6, 600 MHz) of compound **2**. 10
Figure S9. HR-ESI-MS spectrum of **2**. 11
Figure S10. ^{1}H-NMR (DMSO-d_6, 600 MHz) of compound **3**. 12
Figure S11. ^{13}C-NMR (DMSO-d_6, 150 MHz) of compound **3**. 13
Figure S12. HSQC (DMSO-d_6, 600 MHz) of Compound **3**. 14
Figure S13. HMBC (DMSO-d_6, 600 MHz) of compound **3**. 15
Figure S14. COSY (DMSO-d_6, 600 MHz) of compound **3**. 16
Figure S15. ROESY (DMSO-d_6, 600 MHz) of compound **3**. 17
Figure S16. HR-ESI-MS spectrum of **3**. 18
Figure S17. ^{1}H-NMR (DMSO-d_6, 800 MHz) of compound **4**. 19
Figure S18. ^{13}C-NMR (DMSO-d_6, 200 MHz) of compound **4**. 20
Figure S19. HSQC (DMSO-d_6, 800 MHz) of compound **4**. 21
Figure S20. HMBC (DMSO-d_6, 800 MHz) of compound **4**. 22
Figure S21. COSY (DMSO-d_6, 800 MHz) of compound **4**. 23
Figure S22. ROESY (DMSO-d_6, 800 MHz) of compound **4**. 24
Figure S23. HR-ESI-MS spectrum of **4**. 25
Figure S24. ^{1}H-NMR (DMSO-d_6, 600 MHz) of compound **5**. 26
Figure S25. ^{13}C-NMR (DMSO-d_6, 150 MHz) of compound **5**. 27
Figure S26. HSQC (DMSO-d_6, 600 MHz) of Compound **5**. 28
Figure S27. HMBC (DMSO-d_6, 600 MHz) of compound **5**. 29
Figure S28. COSY (DMSO-d_6, 600 MHz) of compound **5**. 30
Figure S29. ROESY (DMSO-d_6, 600 MHz) of compound **5**. 31
Figure S30. HR-ESI-MS spectrum of **5**. 32
Figure S31. ^{1}H-NMR (DMSO-d_6, 600 MHz) of compound **6**. 33
Figure S32. ^{13}C-NMR (DMSO-d_6, 150 MHz) of compound **6**. 34
Figure S33. HSQC (DMSO-d_6, 600 MHz) of Compound **6**. 35
Figure S34. HMBC (DMSO-d_6, 600 MHz) of compound **6**. 36
Figure S35. COSY ($(CD_3)_2SO$, 600 MHz) of compound **6**. 37
Figure S36. ROESY (DMSO-d_6, 600 MHz) of compound **6**. 38
Figure S37. HR-ESI-MS (DMSO-d_6, 600 MHz) spectrum of **6**. 39
Figure S38. ^{1}H-NMR ($CDCl_3$, 600 MHz) of compound **7**. 40
Figure S39. ^{13}C-NMR ($CDCl_3$, 150 MHz) of compound **7**. 41
Figure S40. HSQC ($CDCl_3$, 600 MHz) of Compound **7**. 42
Figure S41. HMBC ($CDCl_3$, 600 MHz) of compound **7**. 43
Figure S42. COSY ($CDCl_3$, 600 MHz) of compound **7**. 44
Figure S43. ROESY ($CDCl_3$, 600 MHz) of compound **7**. 45
Figure S44. HR-ESI-MS spectrum of **7**. 46
Figure S45. The morphology of strain *Scopulariopsis candelabrum* KIB-int20. 47
Figure S46. Broth dilution antifungal susceptibility testing of *Candida albicans*. 48

Figure S47. Inhibiting the growth of mycelial method testing of *Exserohilum turcicum*. 49
Figure S48. Compound **1** (left) and cycloheximide (right) inhibiting the growth of mycelial line. 50
Figure S49. Filter paper agarose diffusion method testing of *Candida albicans* (left) and *Exserohilum turcicum* (right) 51
ITS sequence 52
Figure S50. Proposal biosynthesis pathway of compounds **1**–**7** based on the references. 53
Figure S51. Alkenoic acids reported in the literatures 54
References 54

Table S1. Comparisons of NMR data of carbons with secondary alcohols between references and our project.

**Reported NMR data (ppm) of carbons with secondary alcohols of polyketides in mono-/bis-alkenoic acid derivatives family**	**NMR data (ppm) of C-12(12′) in mono-/bis-alkenoic acid derivatives isolated in our project**
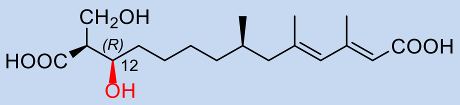<br>**12*R*** in fusaridioic acid A<br>C-12: δ_H 3.55, δ_C 69.3 (in DMSO-d_6)<br>Mar. Drugs 2018, 16(12), 483	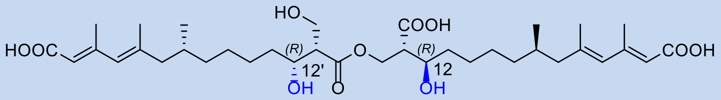<br>**2**<br>**12*R*, 12′*R***<br>C-12: δ_H 3.57, δ_C 68.8; C-12′: δ_H 3.57 , δ_C 68.9 (in DMSO-d_6)
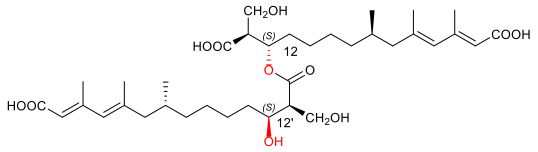<br>**12*S*, 12′*S*** in fusariumester A_1<br>C-12: δ_H 5.00, δ_C 71.6;<br>C-12′: δ_H 3.50, δ_C 69.3 (in DMSO-d_6)<br>Mar. Drugs 2018, 16(12), 483	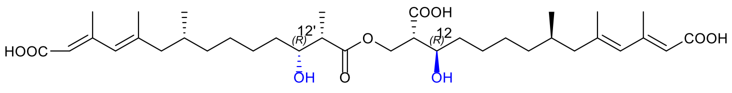<br>**3**<br>**12*R*, 12′*R***<br>C-12: δ_H 3.57, δ_C 69.0; C-12′: δ_H 3.56 , δ_C 71.3 (in DMSO-d_6)
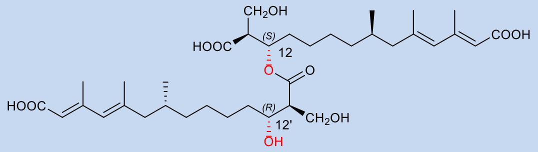<br>**12*S*, 12′*R*** in fusariumester A_2<br>C-12: δ_H 5.00, δ_C 71.3; C-12′: δ_H 3.62, δ_C 69.3 (in DMSO-d_6)<br>Mar. Drugs 2018, 16(12), 483	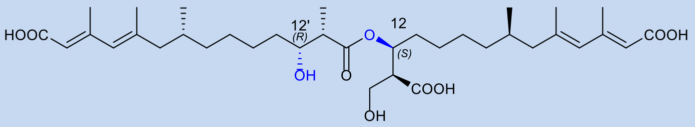<br>**4**<br>**12*S*, 12′*R***<br>C-12: δ_H 4.99, δ_C 71.7; C-12′: δ_H 3.59, δ_C 71.7 (in DMSO-d_6)
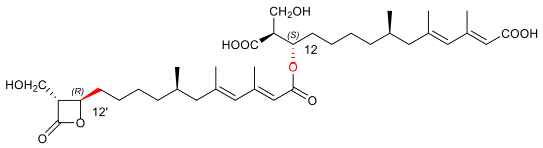<br>**12*S*, 12′*R*** in fusariumester B<br>C-12: δ_H 5.05, δ_C 70.9; C-12′: δ_H 4.53, δ_C 74.7 (in DMSO-d_6)<br>Mar. Drugs 2018, 16(12), 483	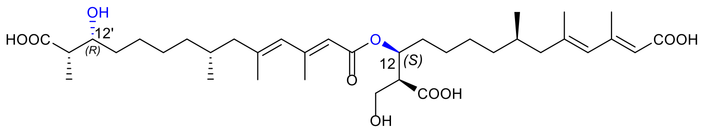<br>**5**<br>**12*S*, 12′*R***<br>C-12: δ_H 5.03, δ_C 70.6; C-12′: δ_H 3.55, δ_C 71.4 (in DMSO-d_6)

Continued Table S1

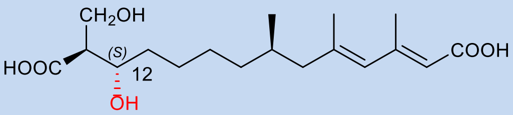<br>**12*S*** in L-660282<br>C-12: δ_H 3.44, δ_C 69.5 (in DMSO-d_6)<br>Mar. Drugs 2018, 16(12), 483	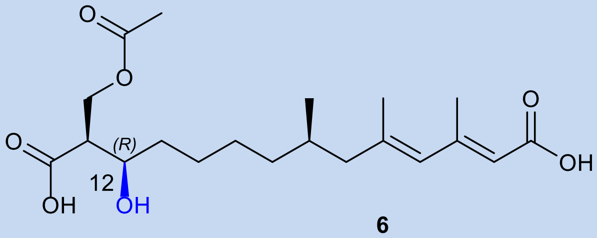<br>**6**<br>**12*R***<br>C-12: δ_H 3.63, δ_C 68.8 (in DMSO-d_6)
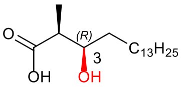<br>**3*R*** in (2*S*,3*R*)-3-Hydroxy-2-methylhexadecanoic acid<br>C-3: δ_H 3.95, δ_C 71.8 (in $CDCl_3$)<br>Chem. Eur. J. 2008, 14, 8847	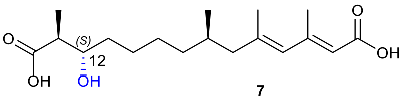<br>**7**<br>**12*S***<br>C-12: δ_H 3.69, δ_C 73.2 (in $CDCl_3$)
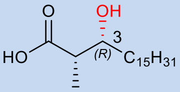<br>**3*R*** in (2*S*,3*R*)-3-hydroxy-2-methyloctadecanoic acid<br>C-3: δ_H 3.98 (in $CDCl_3$)<br>Tetrahedron Lett. 2007, 48, 8104	
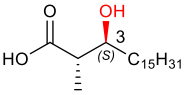<br>**3*S*** in<br>(2*S*,3*S*)-3-hydroxy-2-methyloctadecanoic acid<br>C-3: δ_H 3.72 (in $CDCl_3$)<br>Tetrahedron Lett. 2007, 48, 8104	

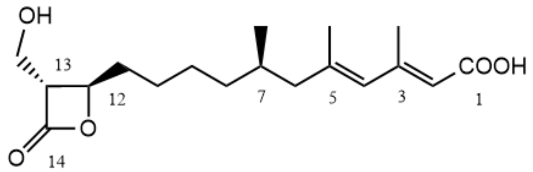

1

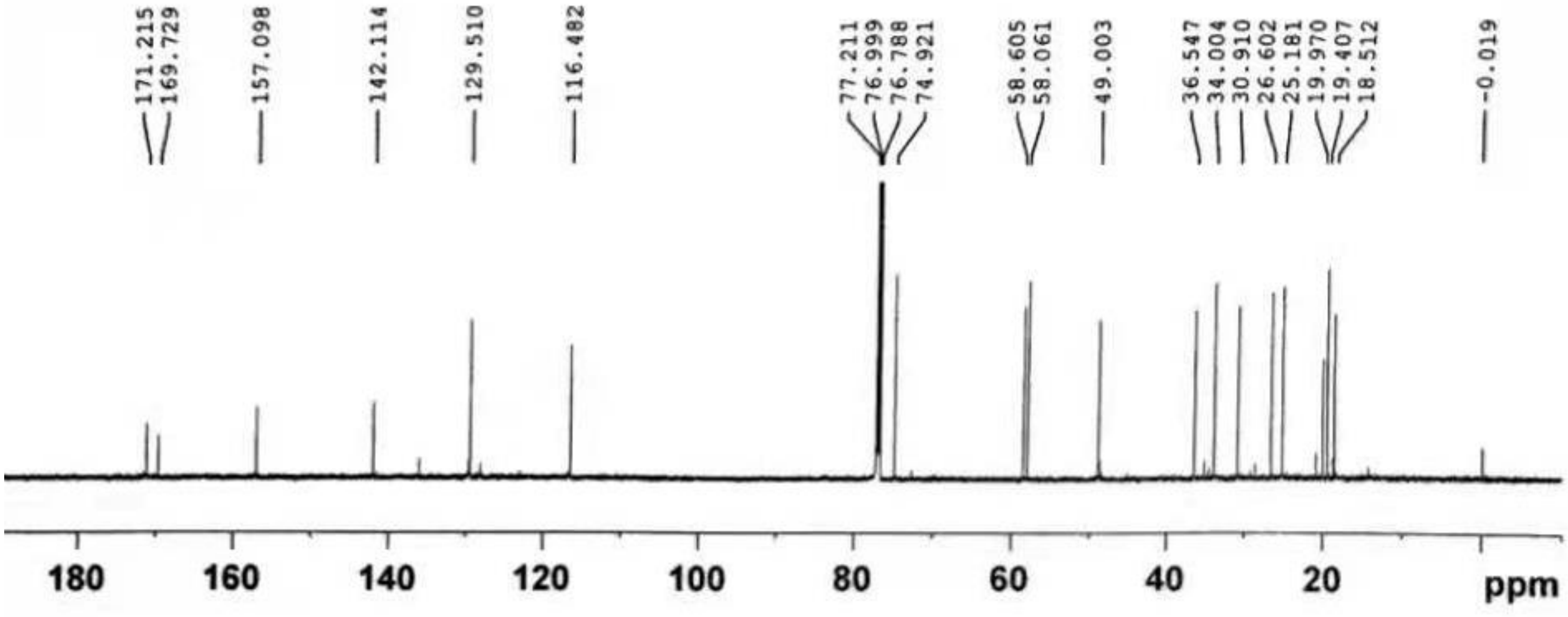

Figure S1. ^{13}C-NMR ($CDCl_3$, 150 MHz) of compound **1**.

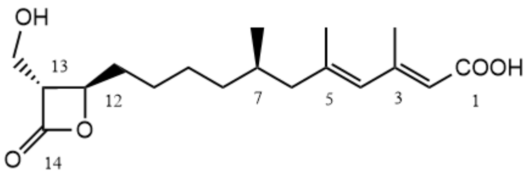

1

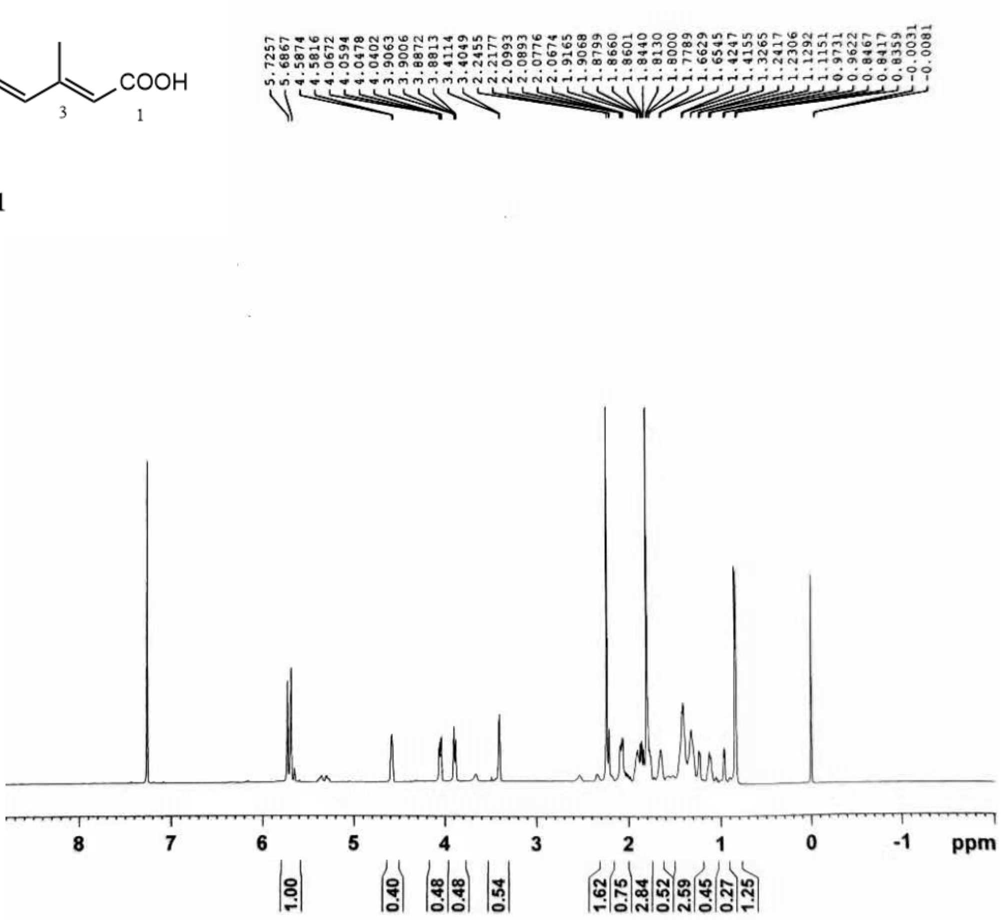

Figure S2. ^{1}H-NMR ($CDCl_3$, 600 MHz) of compound **1**.

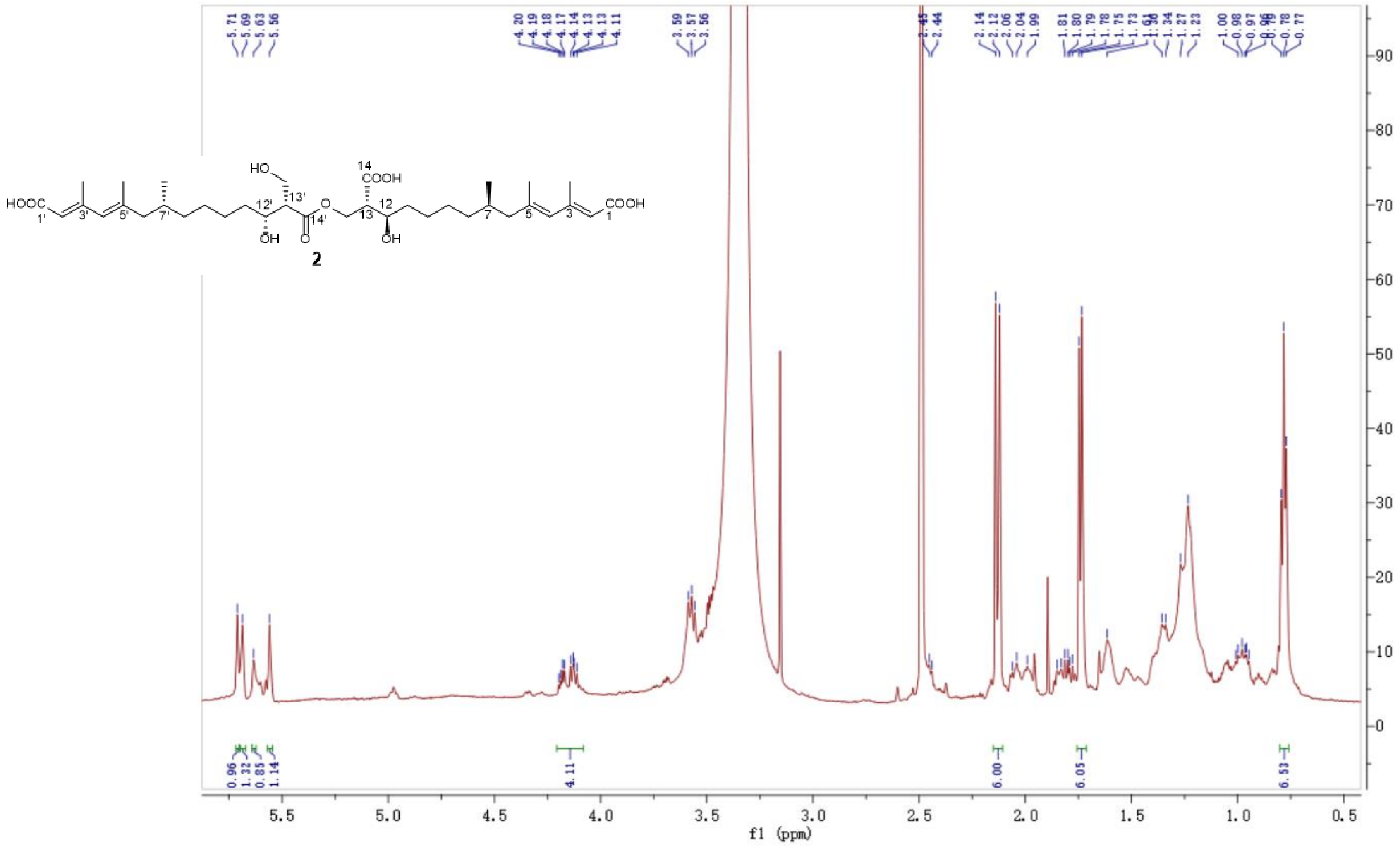

Figure S3. ^{1}H-NMR (DMSO-d_6, 600 MHz) of compound **2**.

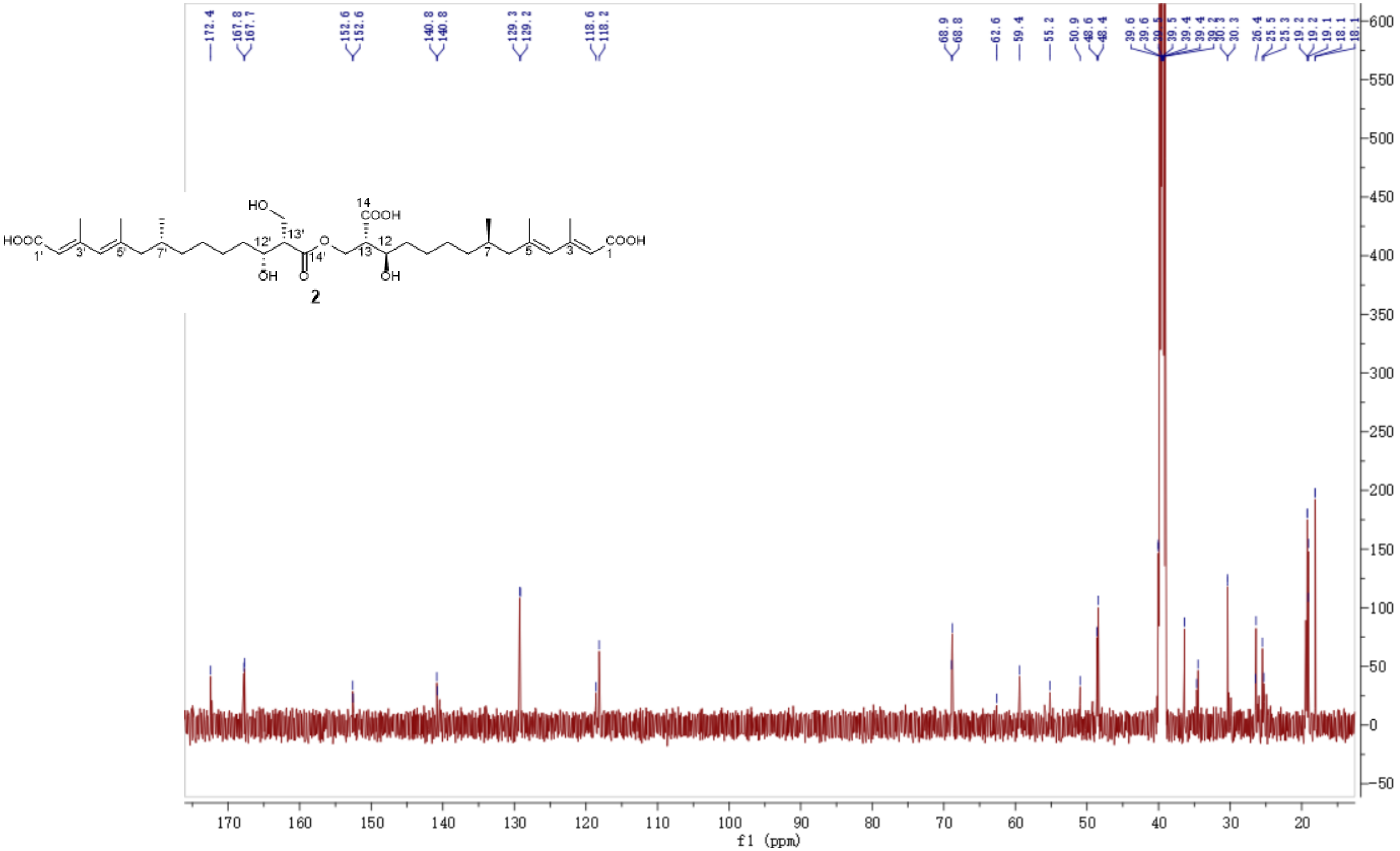

Figure S4. ^{13}C-NMR (DMSO-d_6, 150 MHz) of compound **2**.

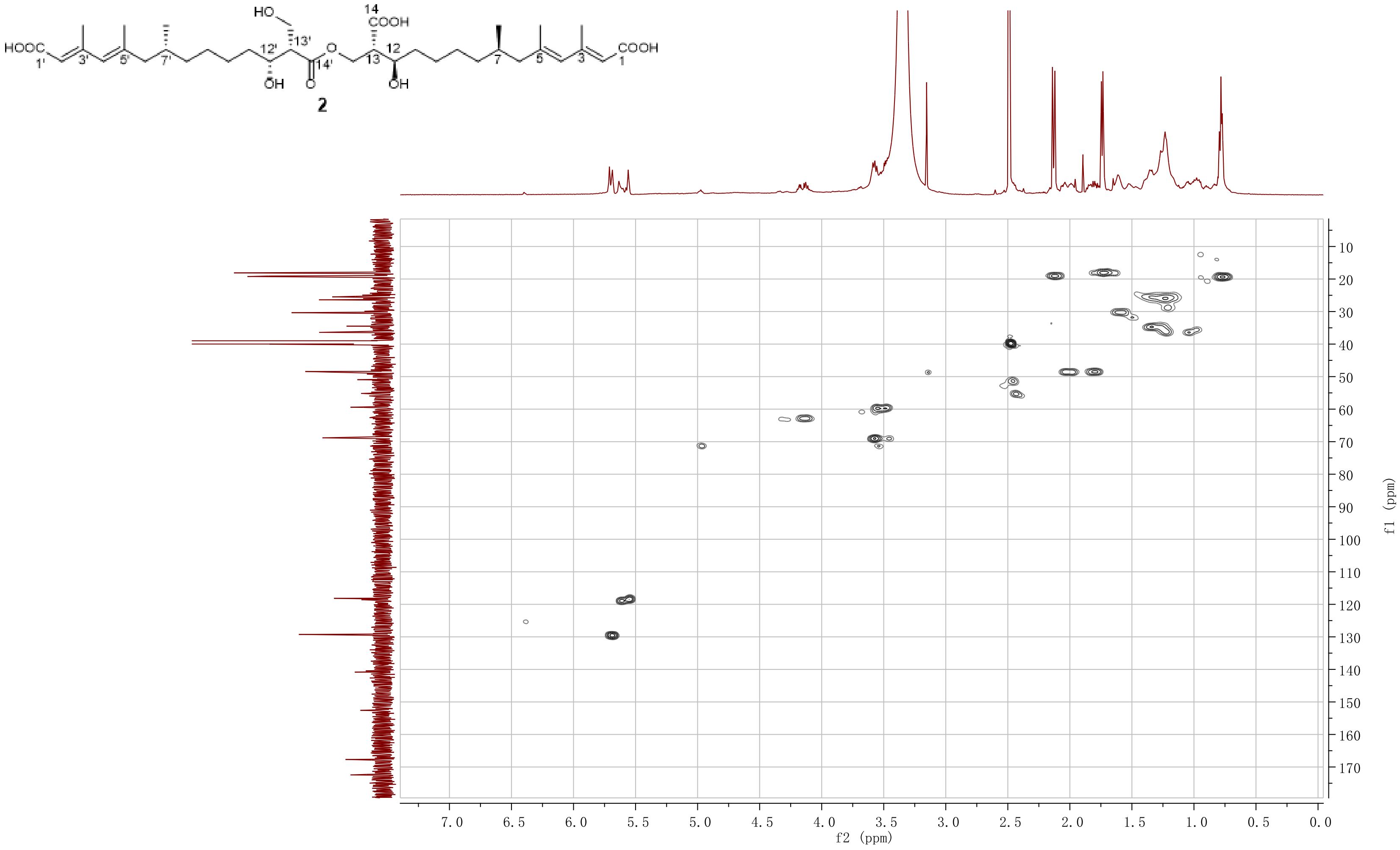

2

Figure S5. HSQC (DMSO-d_6, 600 MHz) of Compound **2**.

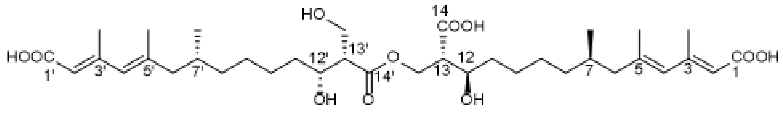

2

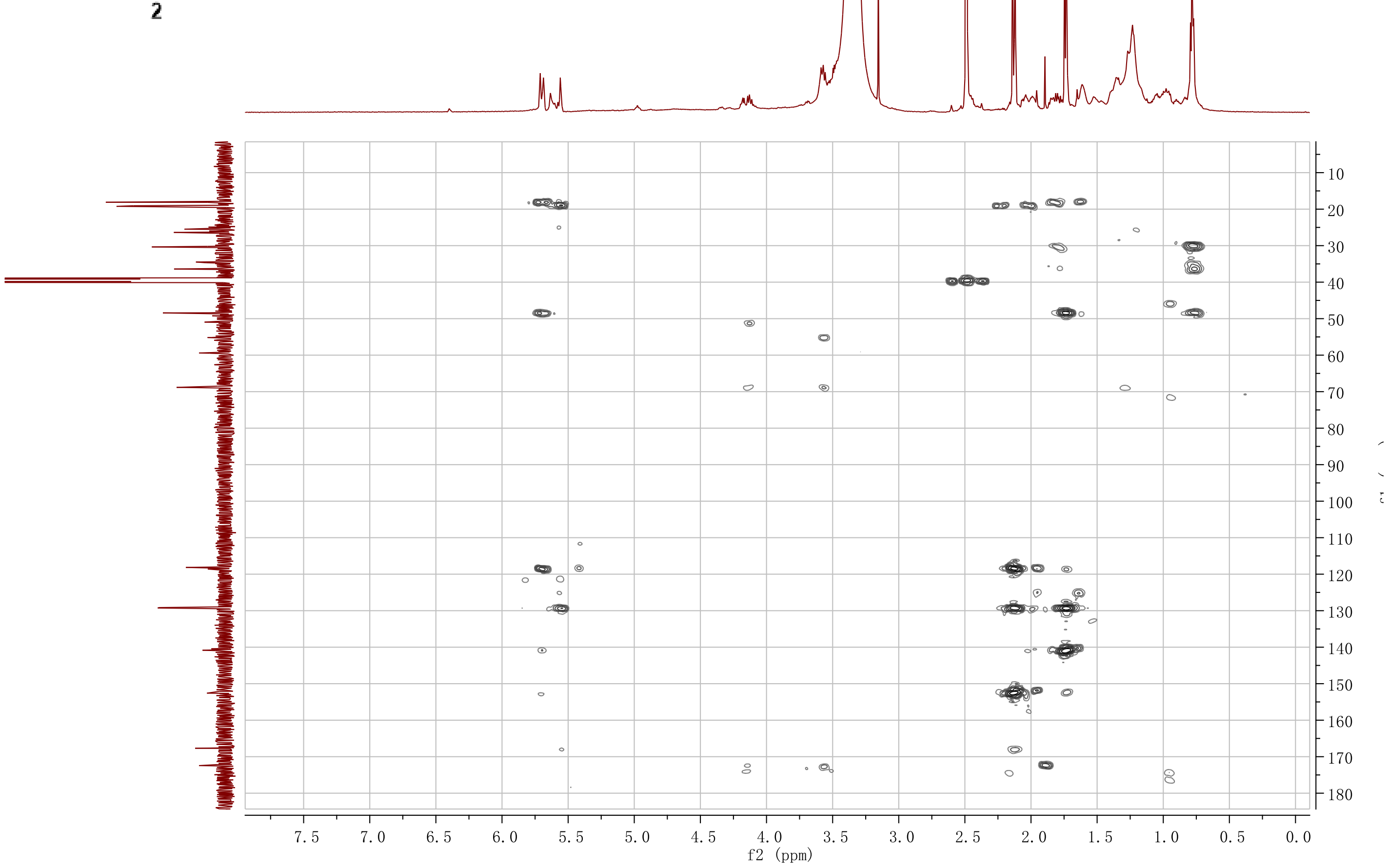

Figure S6. HMBC (DMSO-d_6, 600 MHz) of compound **2**.

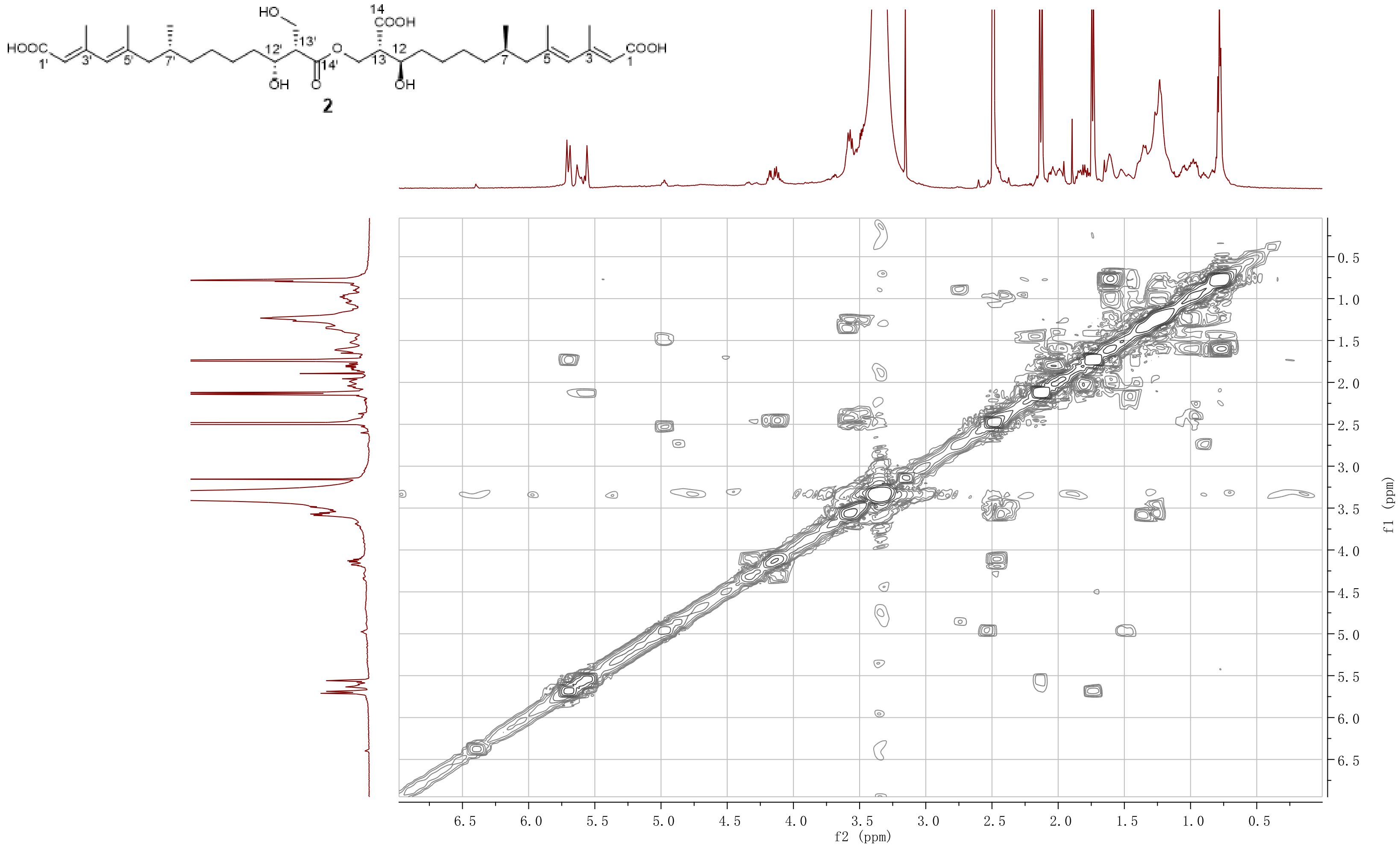

Figure S7. COSY (DMSO-d_6, 600 MHz) of compound **2**.

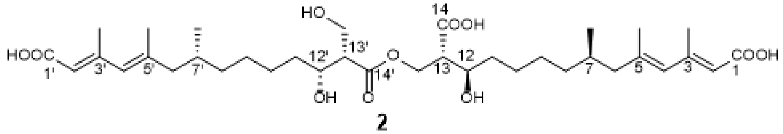

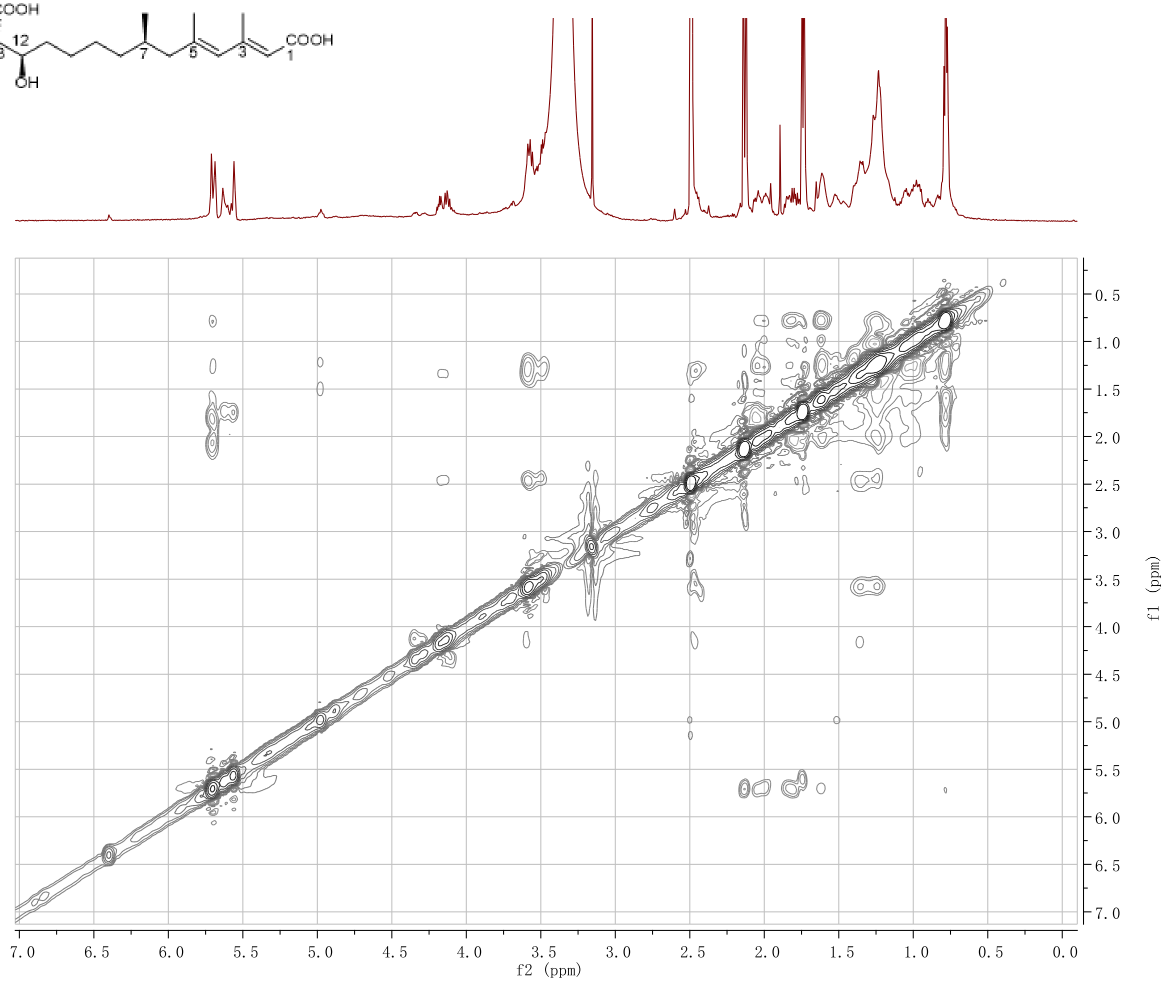

Figure S8. ROESY (DMSO-d_6, 600 MHz) of compound **2**.

Data File: E:\DATA\2020\0929\int20-7.lcd

Elmt	Val.	Min	Max	Elmt	Val.	Min	Max	Elmt	Val.	Min	Max	Elmt	Val.	Min	Max	Use Adduct
H	1	10	110	F	1	0	0	S	2	0	0	Br	1	0	0	H
2H	1	0	0	Na	1	0	0	Cl	1	0	0	Pd	2	0	0	
C	4	5	50	Mg	2	0	0	Co	2	0	0	Ag	1	0	0	
N	3	0	10	Si	4	0	0	Cu	2	0	0	I	3	0	0	
O	2	0	40	P	3	0	0	Se	2	0	0					

Error Margin (ppm):	5	DBE Range:	-2.0 - 100.0	Electron Ions:	both
HC Ratio:	unlimited	Apply N Rule:	yes	Use MSn Info:	yes
Max Isotopes:	all	Isotope RI (%):	1.00	Isotope Res:	10000
MSn Iso RI (%):	75.00	MSn Logic Mode:	OR	Max Results:	10

Event#: 2 MS(E-) Ret. Time : 0.453 Scan# : 70

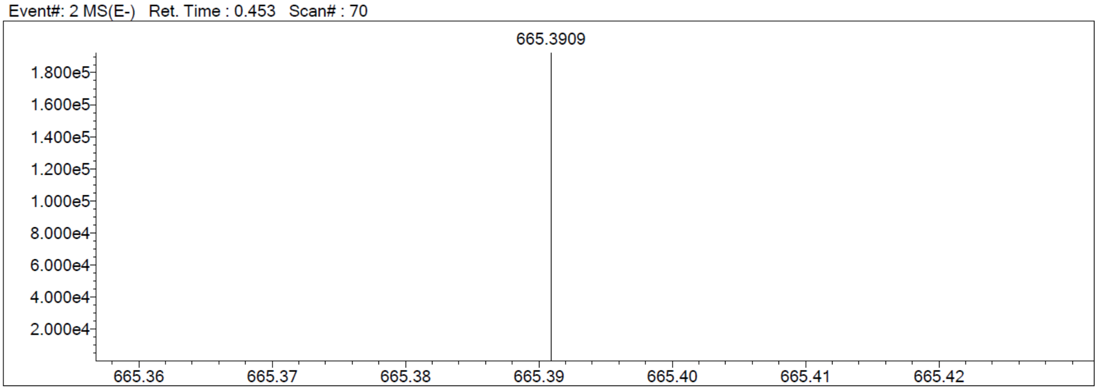

Measured region for 665.3909 m/z

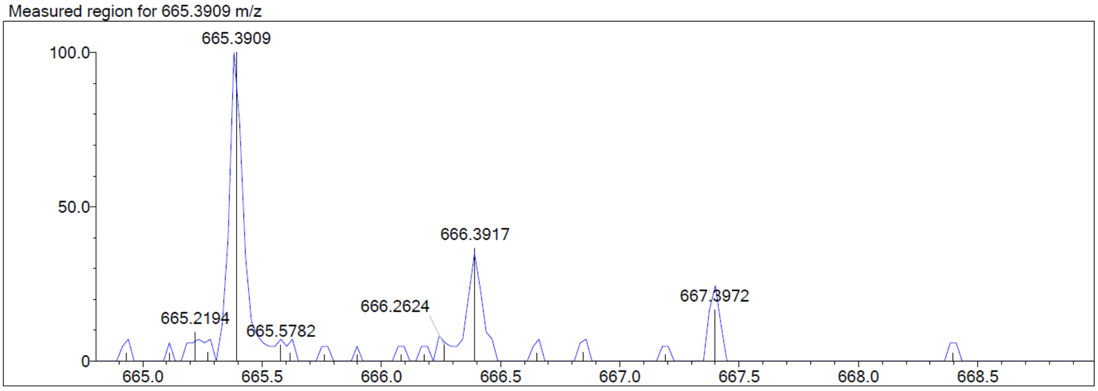

C36 H58 O11 [M-H]- : Predicted region for 665.3906 m/z

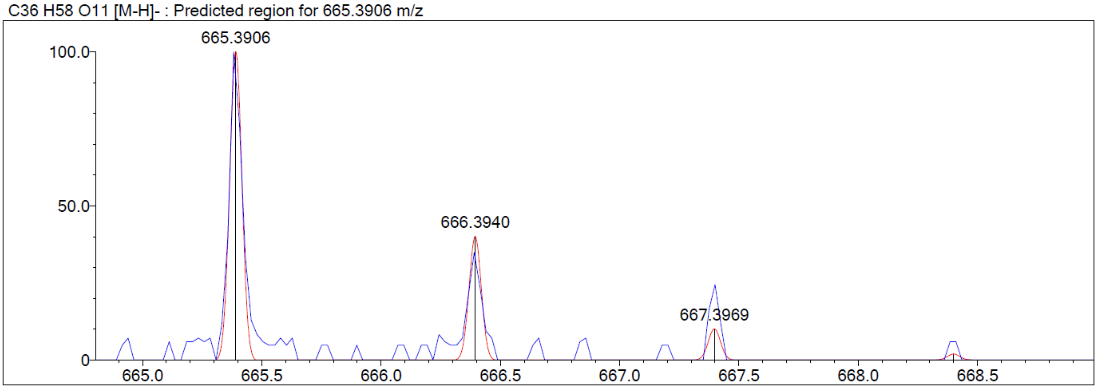

Formula (M)	Ion	Meas. m/z	Pred. m/z	Df. (mDa)	Df. (ppm)	DBE
C36 H58 O11	[M-H]-	665.3909	665.3906	0.3	0.45	8.0

Figure S9. HR-ESI-MS spectrum of **2**.

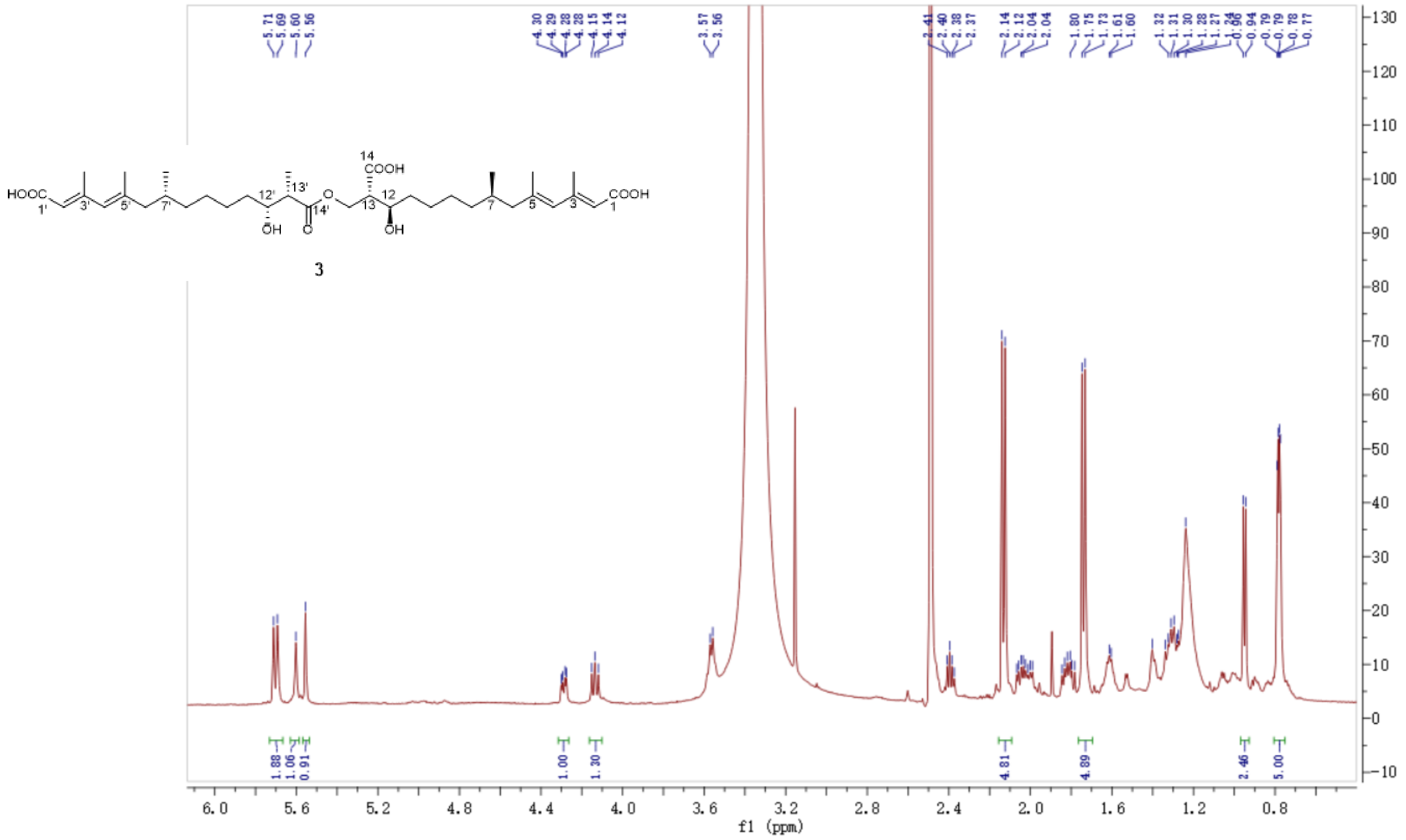

Figure S10. ^{1}H-NMR (DMSO-d_6, 600 MHz) of compound **3**.

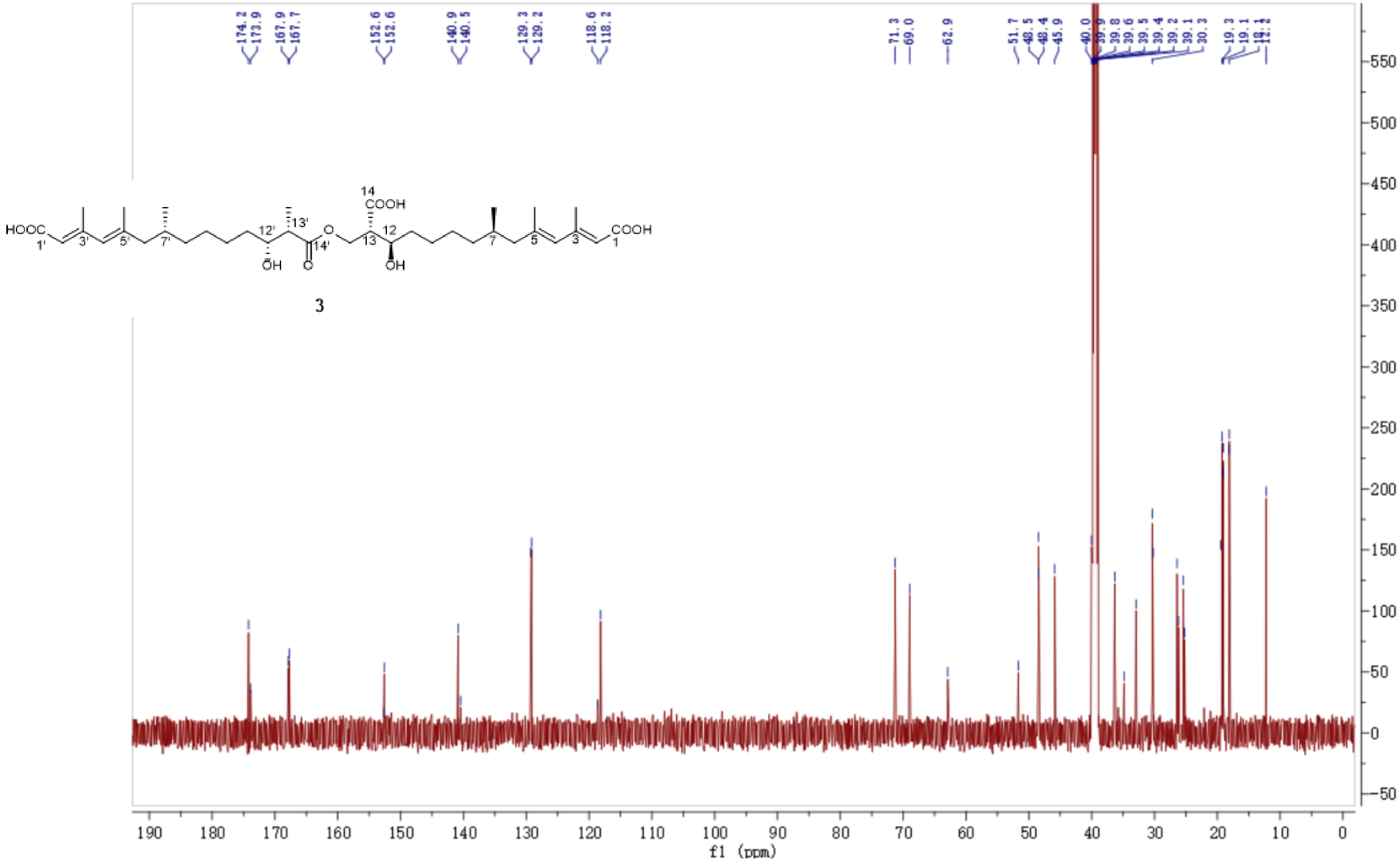

Figure S11. ^{13}C-NMR (DMSO-d_6, 150 MHz) of compound **3**.

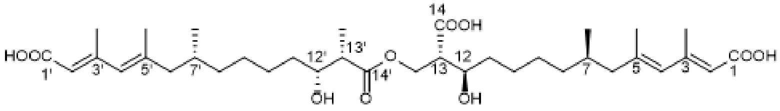

3

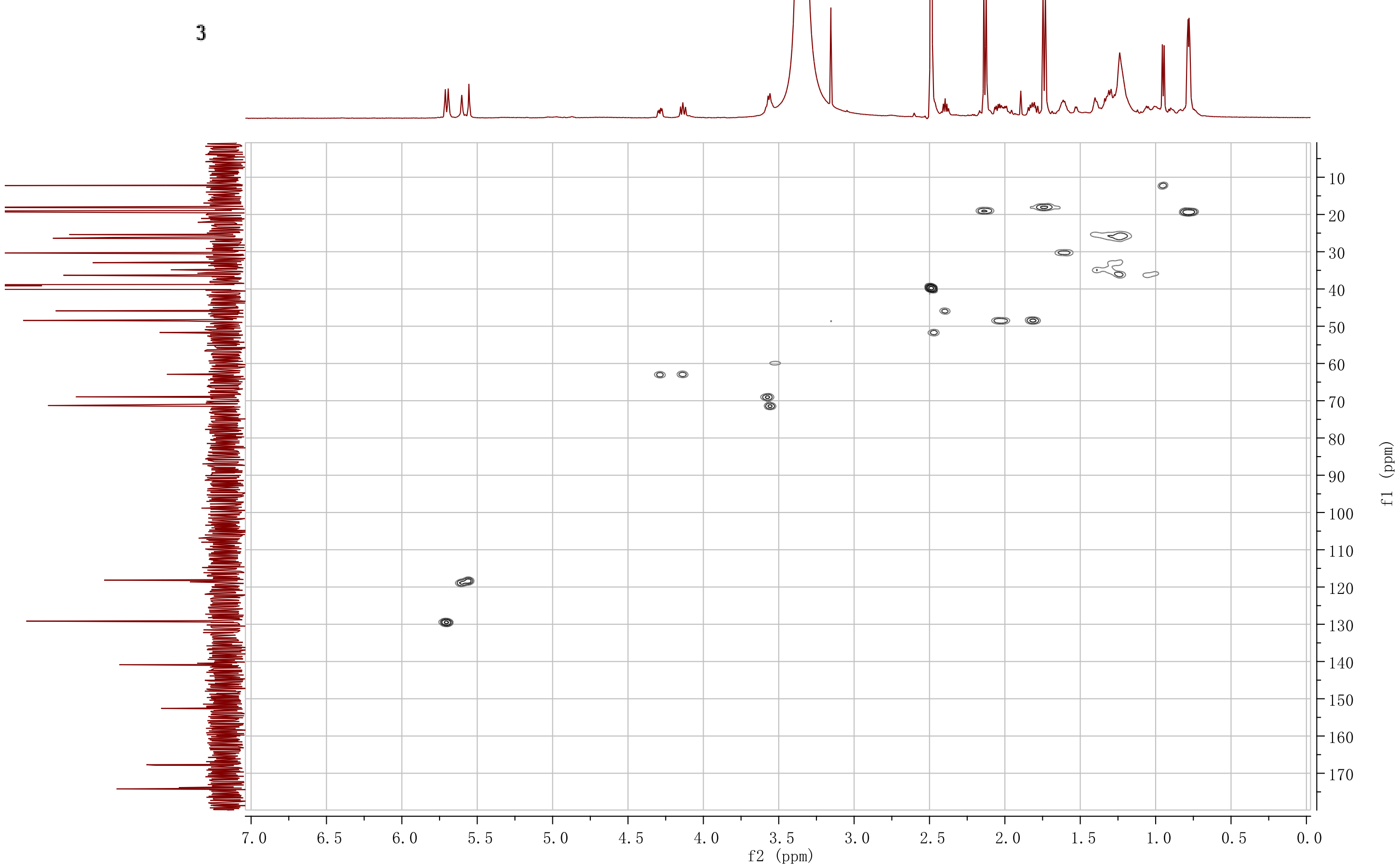

Figure S12. HSQC (DMSO-d_6, 600 MHz) of Compound **3**.

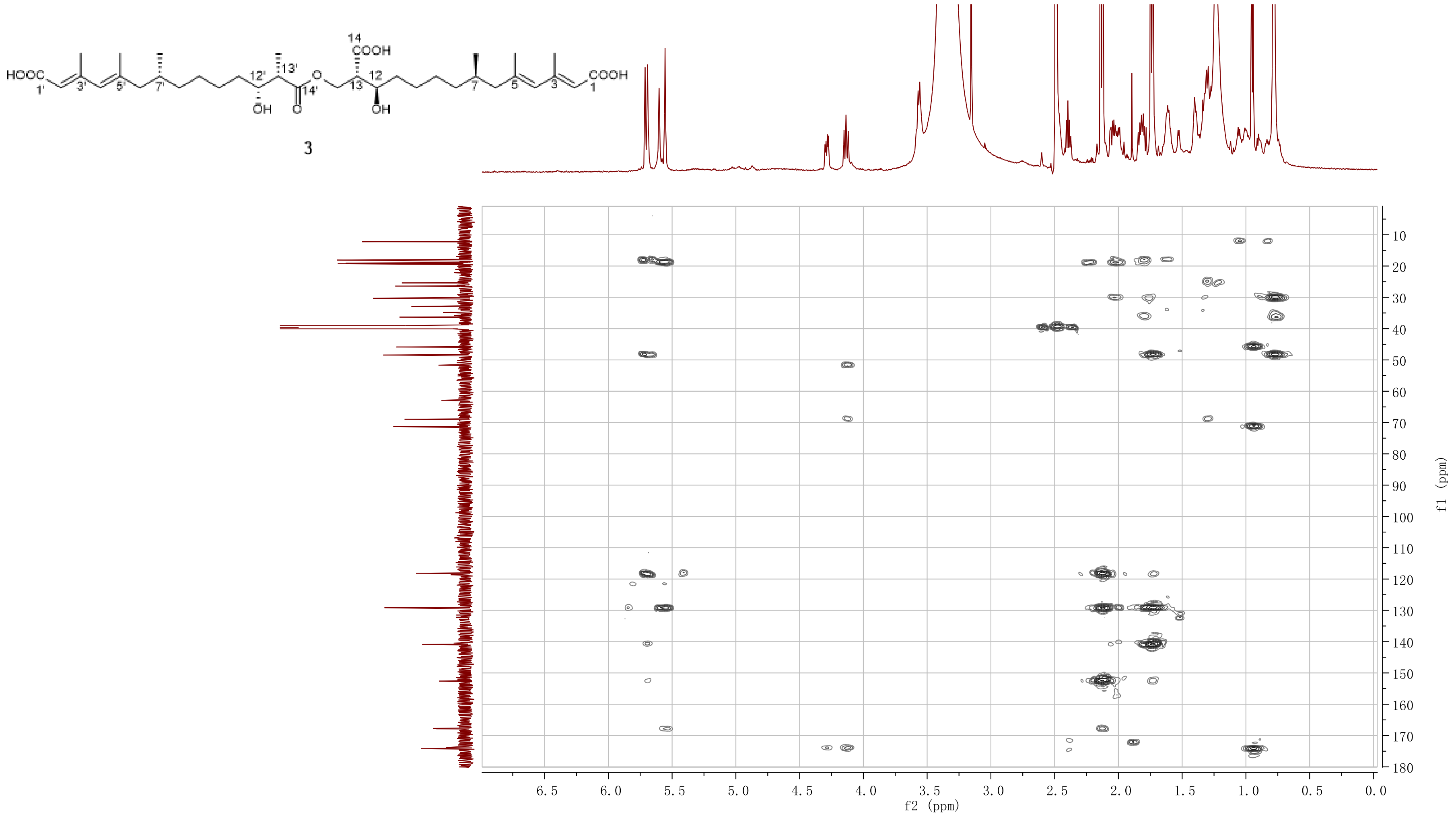

Figure S13. HMBC (DMSO-d_6, 600 MHz) of compound **3**.

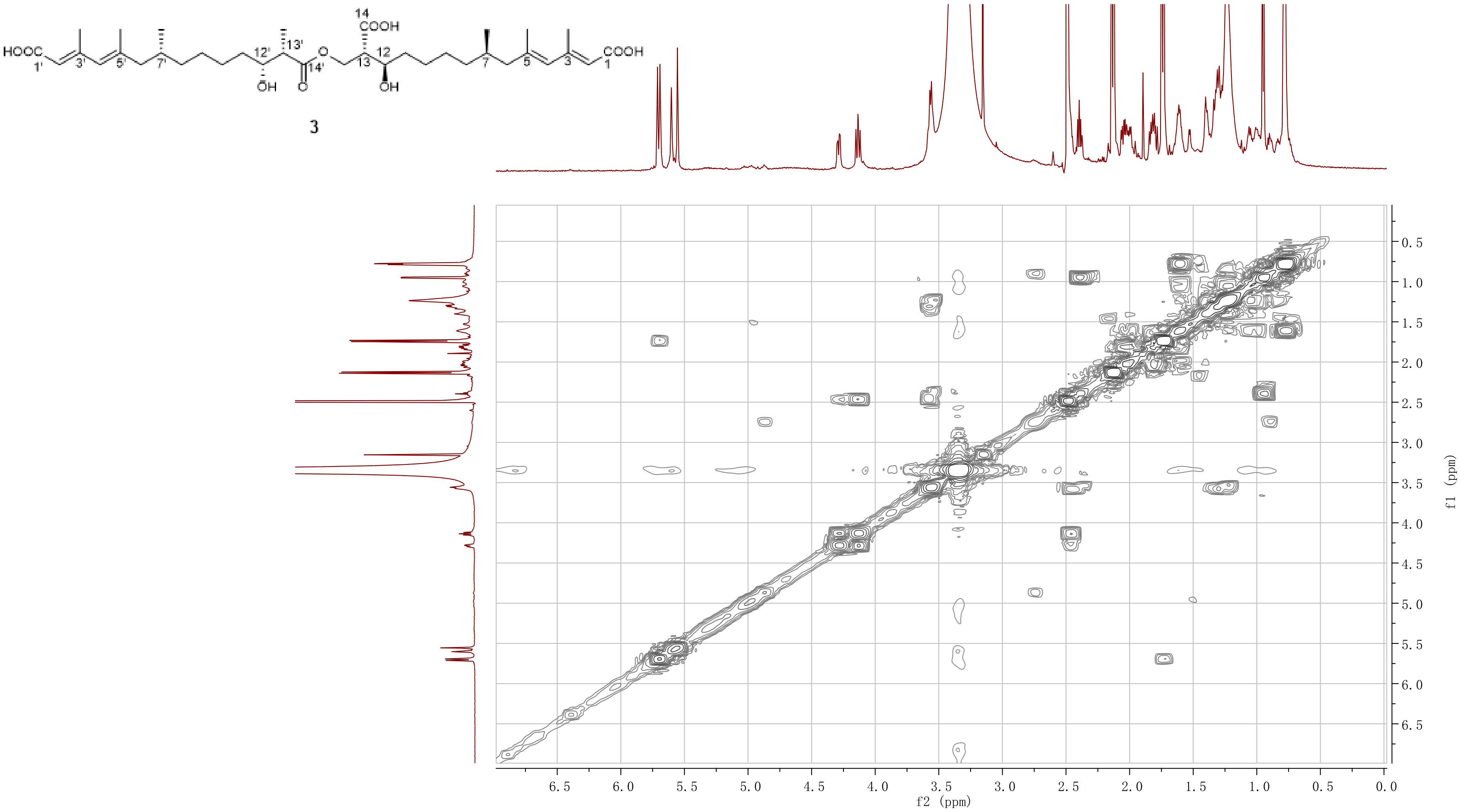

Figure S14. COSY (DMSO-d_6, 600 MHz) of compound **3**.

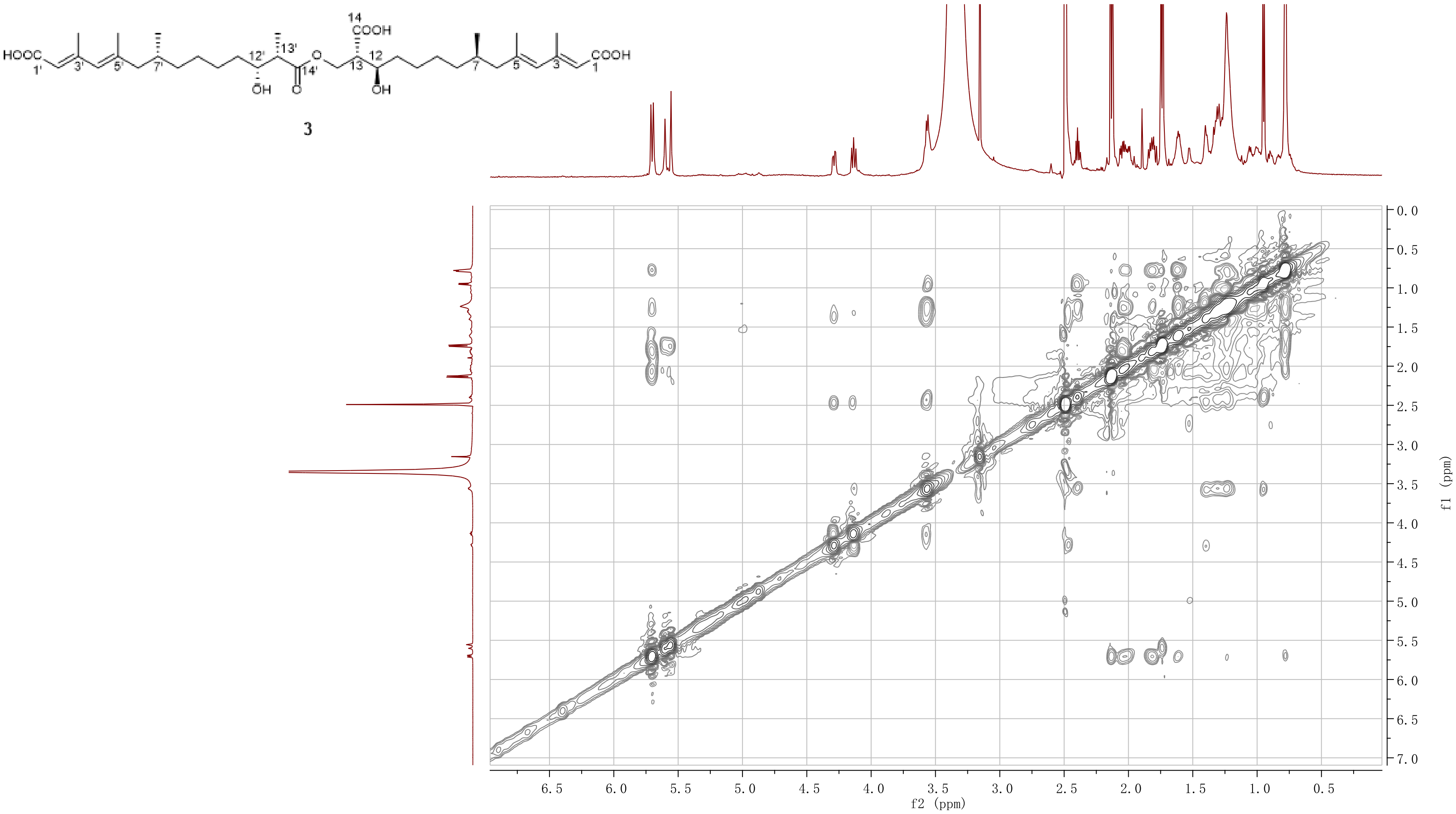

Figure S15. ROESY (DMSO-d_6, 600 MHz) of compound **3**.

Data File: E:\DATA\2020\0929\int20-8.lcd

Elmt	Val.	Min	Max	Elmt	Val.	Min	Max	Elmt	Val.	Min	Max	Elmt	Val.	Min	Max	Use Adduct
H	1	10	110	F	1	0	0	S	2	0	0	Br	1	0	0	H
2H	1	0	0	Na	1	0	0	Cl	1	0	0	Pd	2	0	0	
C	4	5	50	Mg	2	0	0	Co	2	0	0	Ag	1	0	0	
N	3	0	10	Si	4	0	0	Cu	2	0	0	I	3	0	0	
O	2	0	40	P	3	0	0	Se	2	0	0					

Error Margin (ppm):	5	DBE Range:	-2.0 - 100.0	Electron Ions:	both
HC Ratio:	unlimited	Apply N Rule:	yes	Use MSn Info:	yes
Max Isotopes:	all	Isotope RI (%):	1.00	Isotope Res:	10000
MSn Iso RI (%):	75.00	MSn Logic Mode:	OR	Max Results:	10

Event#: 2 MS(E-) Ret. Time : 0.480 Scan# : 74

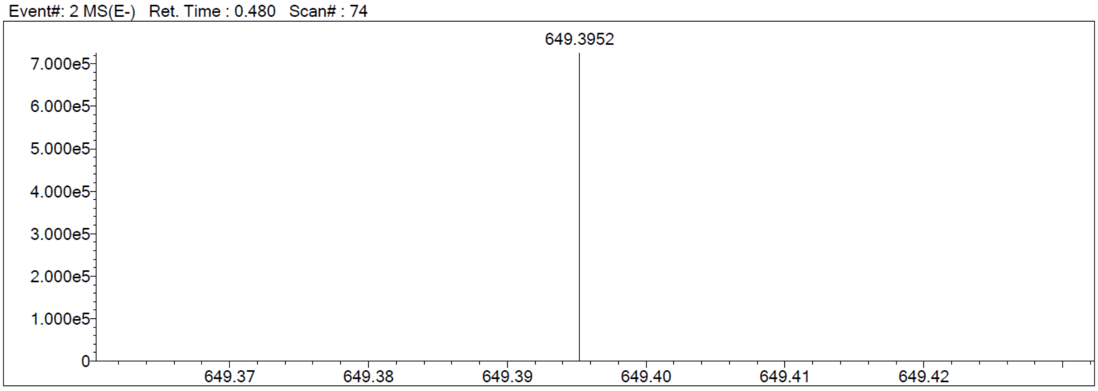

Measured region for 649.3952 m/z

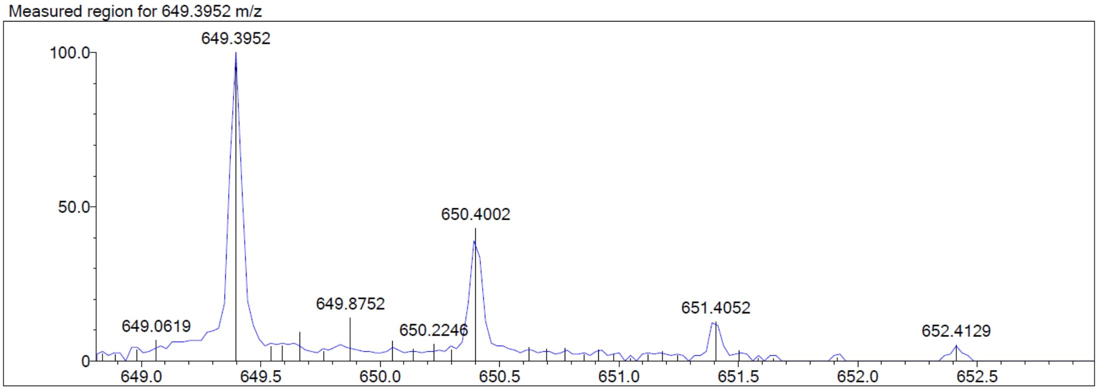

C36 H58 O10 [M-H]- : Predicted region for 649.3957 m/z

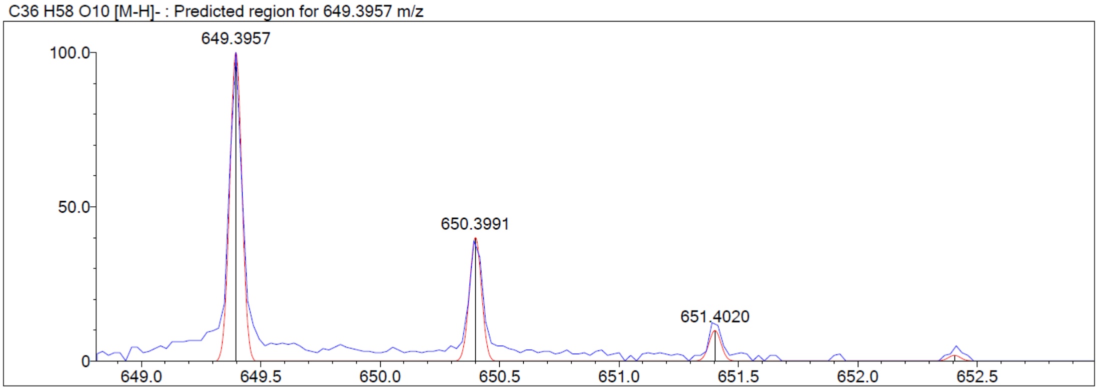

Formula (M)	Ion	Meas. m/z	Pred. m/z	Df. (mDa)	Df. (ppm)	DBE
C36 H58 O10	[M-H]-	649.3952	649.3957	-0.5	-0.77	8.0

Figure S16. HR-ESI-MS spectrum of **3**.

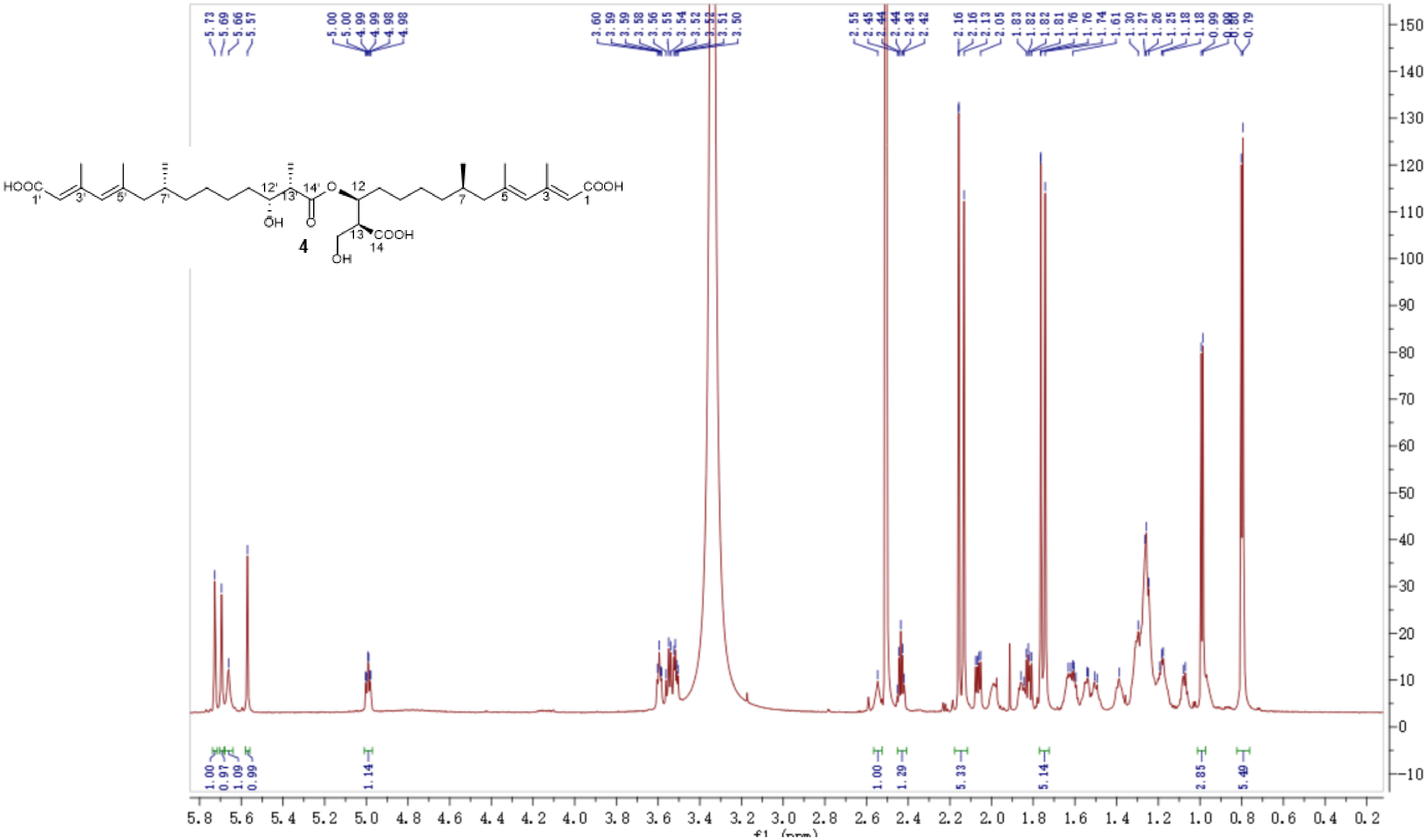

Figure S17. ^{1}H-NMR (DMSO-d_6, 800 MHz) of compound **4**.

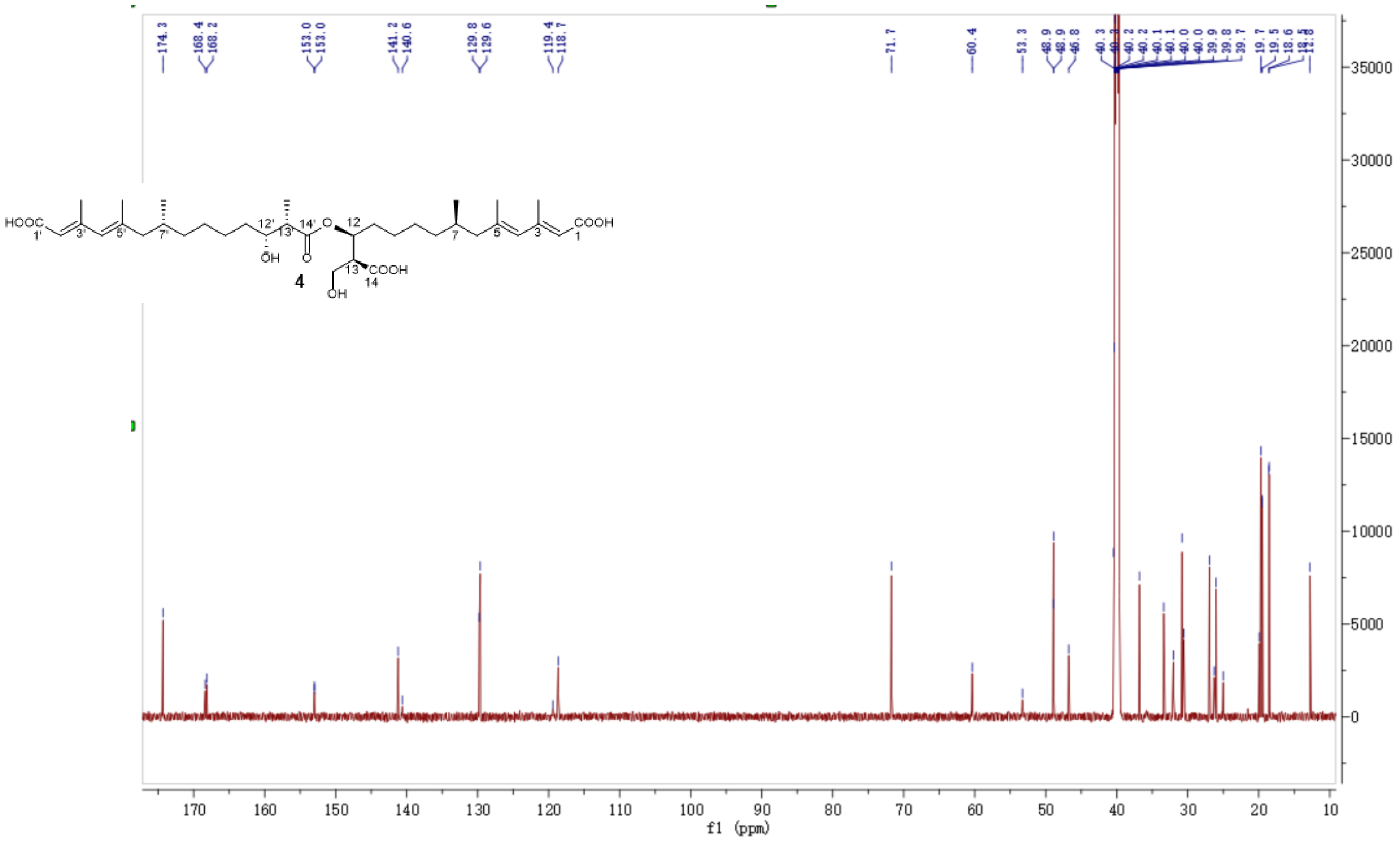

Figure S18. ^{13}C-NMR (DMSO-d_6, 200 MHz) of compound **4**.

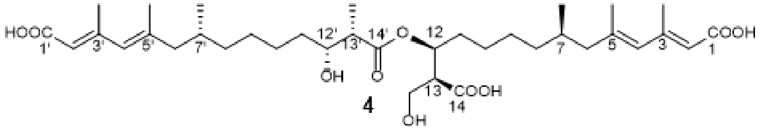

4

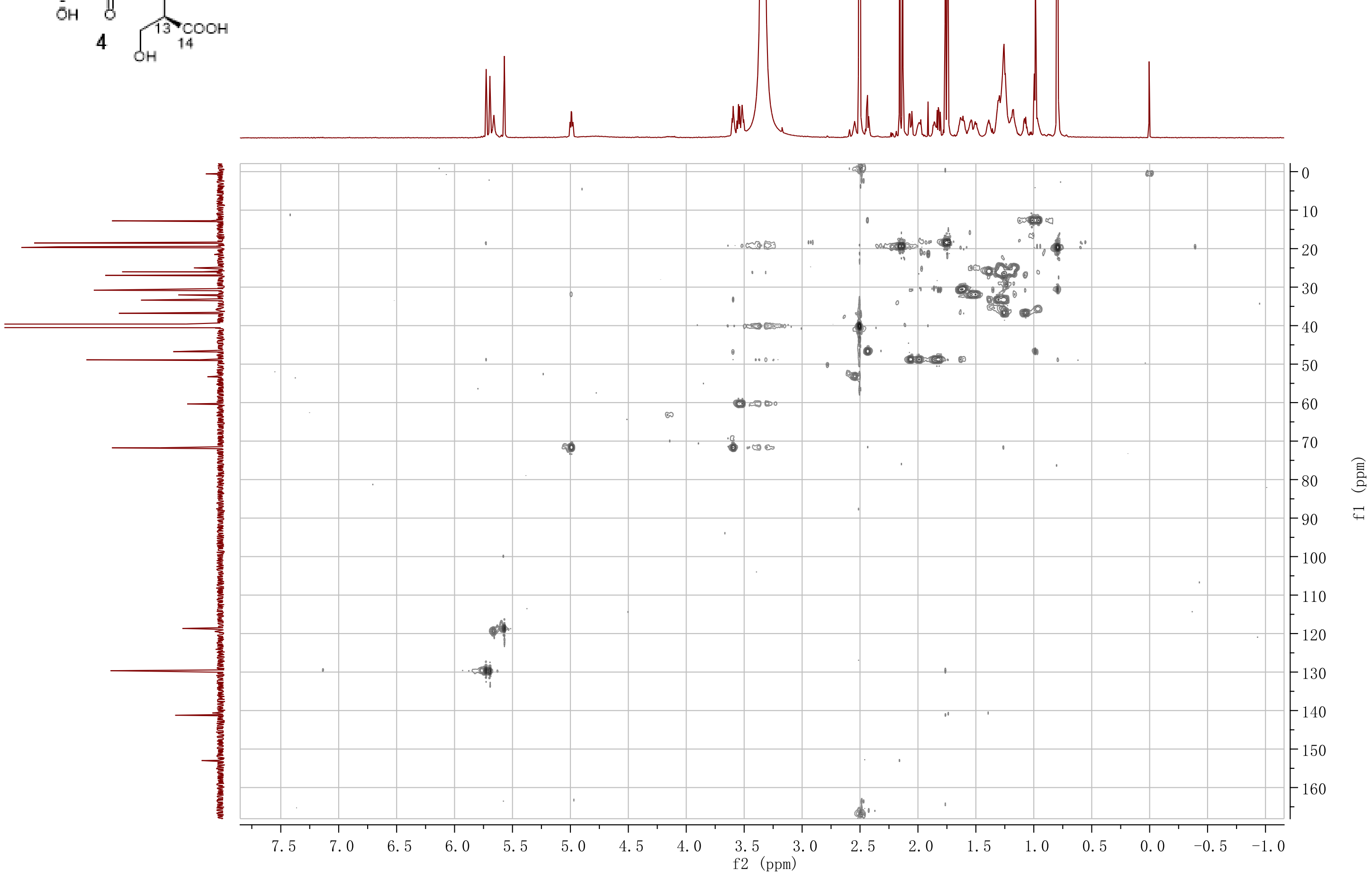

Figure S19. HSQC (DMSO-d_6, 800 MHz) of Compound **4**.

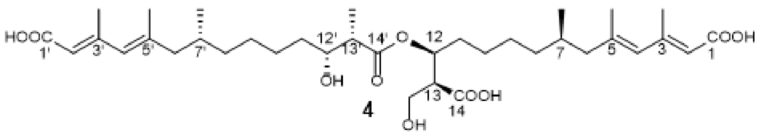

4

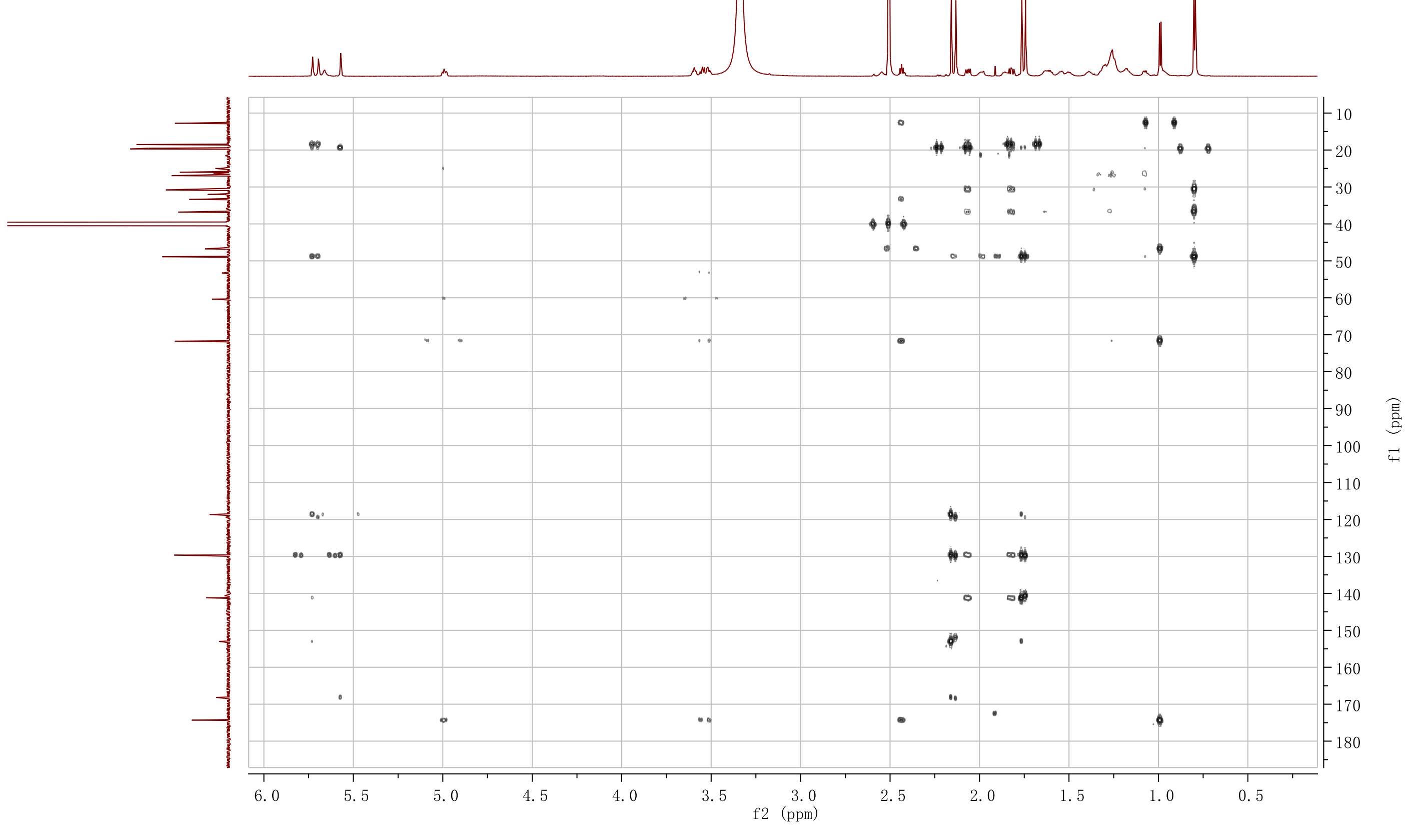

Figure S20. HMBC (DMSO-d_6, 800 MHz) of compound **4**.

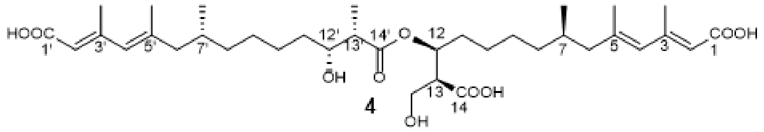

4

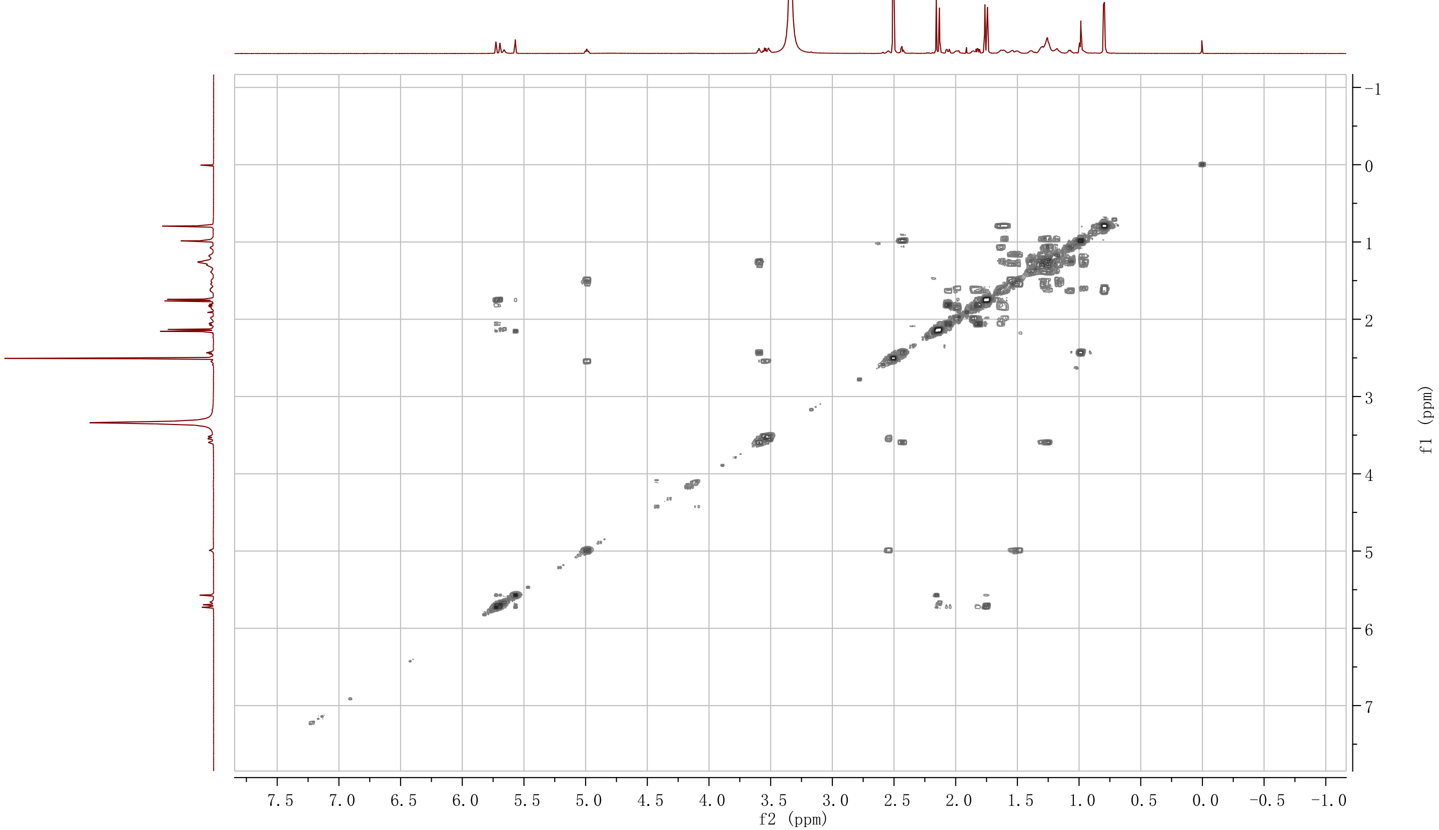

Figure S21. COSY (DMSO-d_6, 800 MHz) of compound **4**.

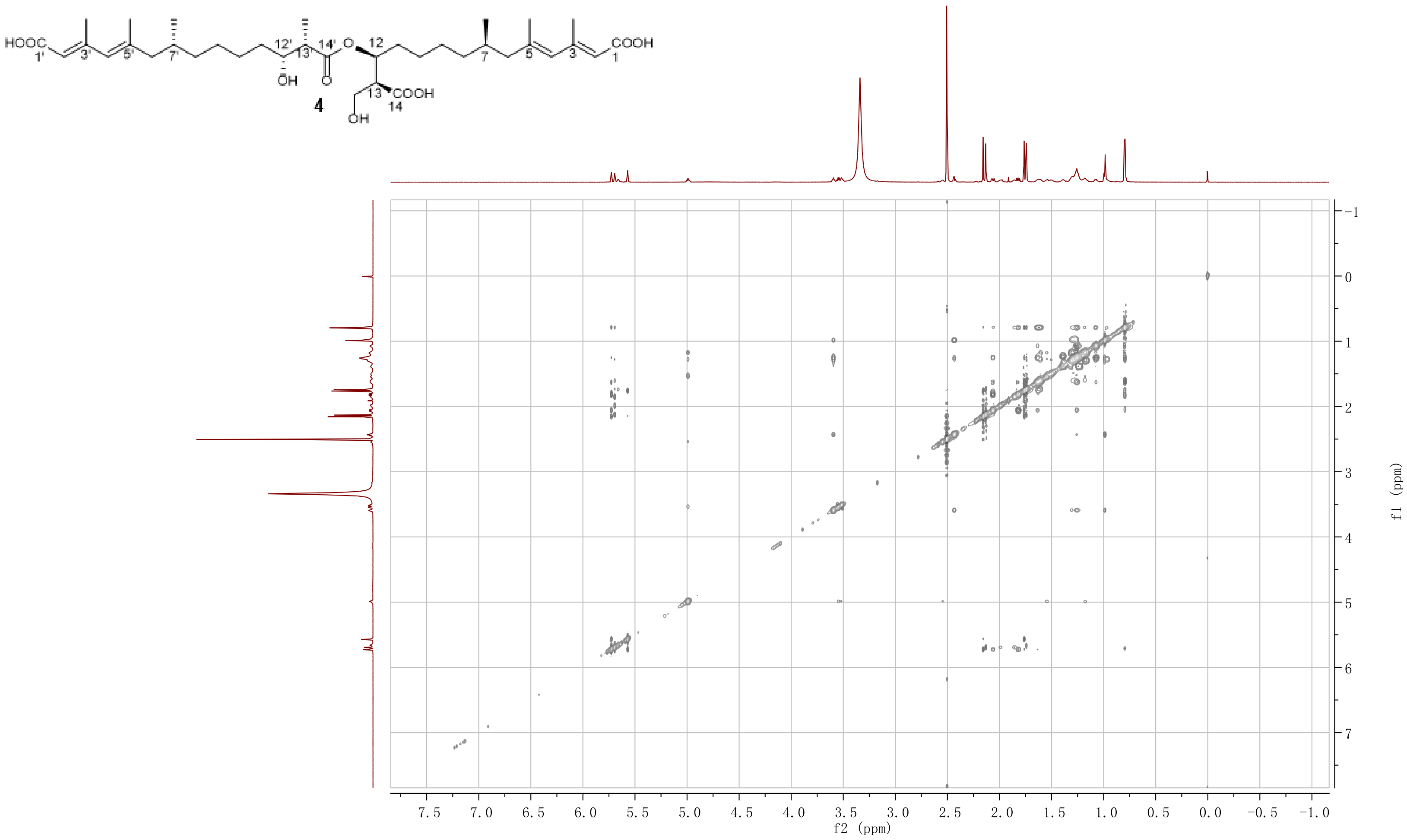

Figure S22. ROESY (DMSO-d_6, 800 MHz) of compound **4**.

Data File: E:\DATA\2020\0929\int20-9.lcd

Elmt	Val.	Min	Max	Elmt	Val.	Min	Max	Elmt	Val.	Min	Max	Elmt	Val.	Min	Max	Use Adduct
H	1	10	110	F	1	0	0	S	2	0	0	Br	1	0	0	H
2H	1	0	0	Na	1	0	0	Cl	1	0	0	Pd	2	0	0	
C	4	5	50	Mg	2	0	0	Co	2	0	0	Ag	1	0	0	
N	3	0	10	Si	4	0	0	Cu	2	0	0	I	3	0	0	
O	2	0	40	P	3	0	0	Se	2	0	0					

Error Margin (ppm):	5	DBE Range:	-2.0 - 100.0	Electron Ions:	both
HC Ratio:	unlimited	Apply N Rule:	yes	Use MSn Info:	yes
Max Isotopes:	all	Isotope RI (%):	1.00	Isotope Res:	10000
MSn Iso RI (%):	75.00	MSn Logic Mode:	OR	Max Results:	10

Event#: 2 MS(E-) Ret. Time : 0.387 Scan# : 60

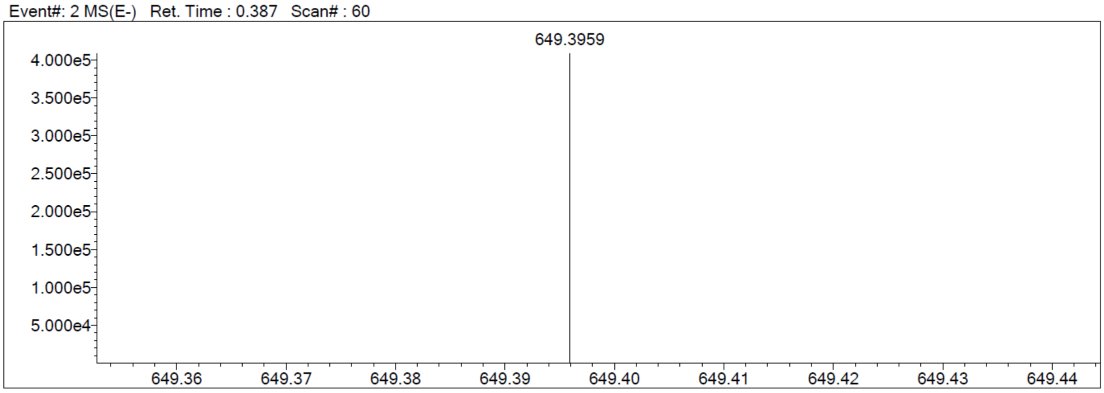

Measured region for 649.3959 m/z

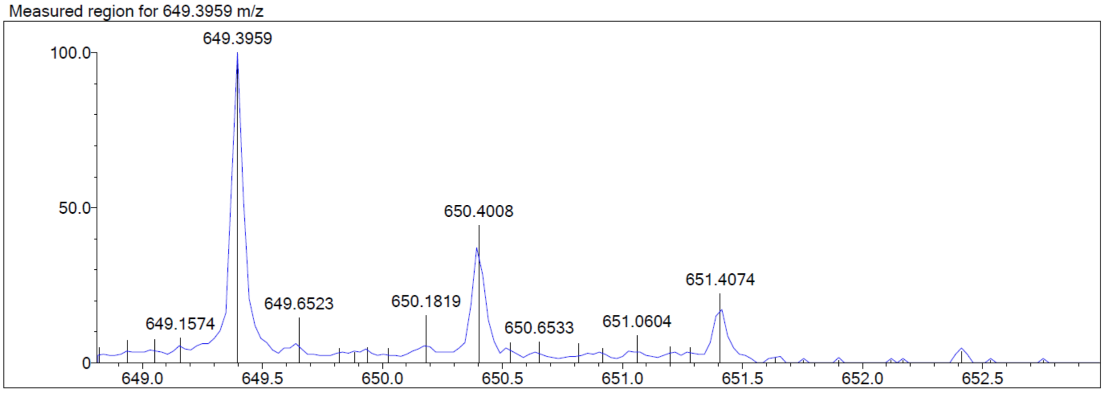

C36 H58 O10 [M-H]- : Predicted region for 649.3957 m/z

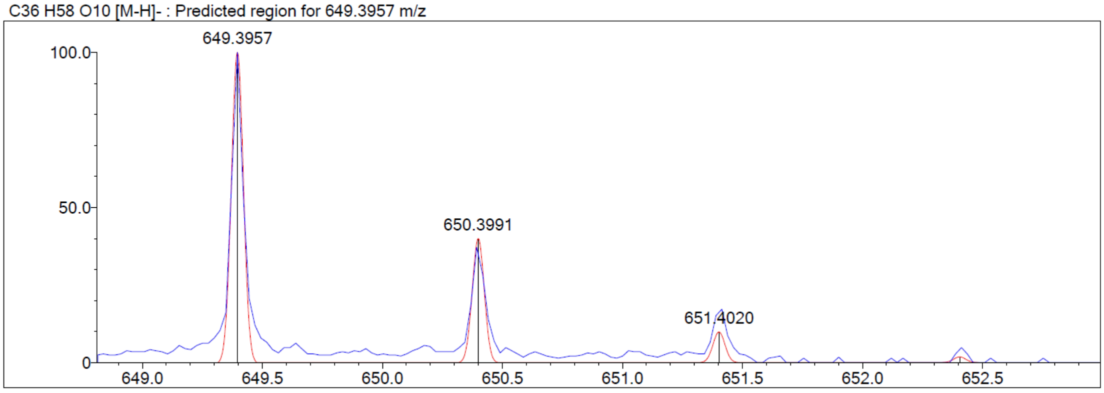

Formula (M)	Ion	Meas. m/z	Pred. m/z	Df. (mDa)	Df. (ppm)	DBE
C36 H58 O10	[M-H]-	649.3959	649.3957	0.2	0.31	8.0

Figure S23. HR-ESI-MS spectrum of **4**.

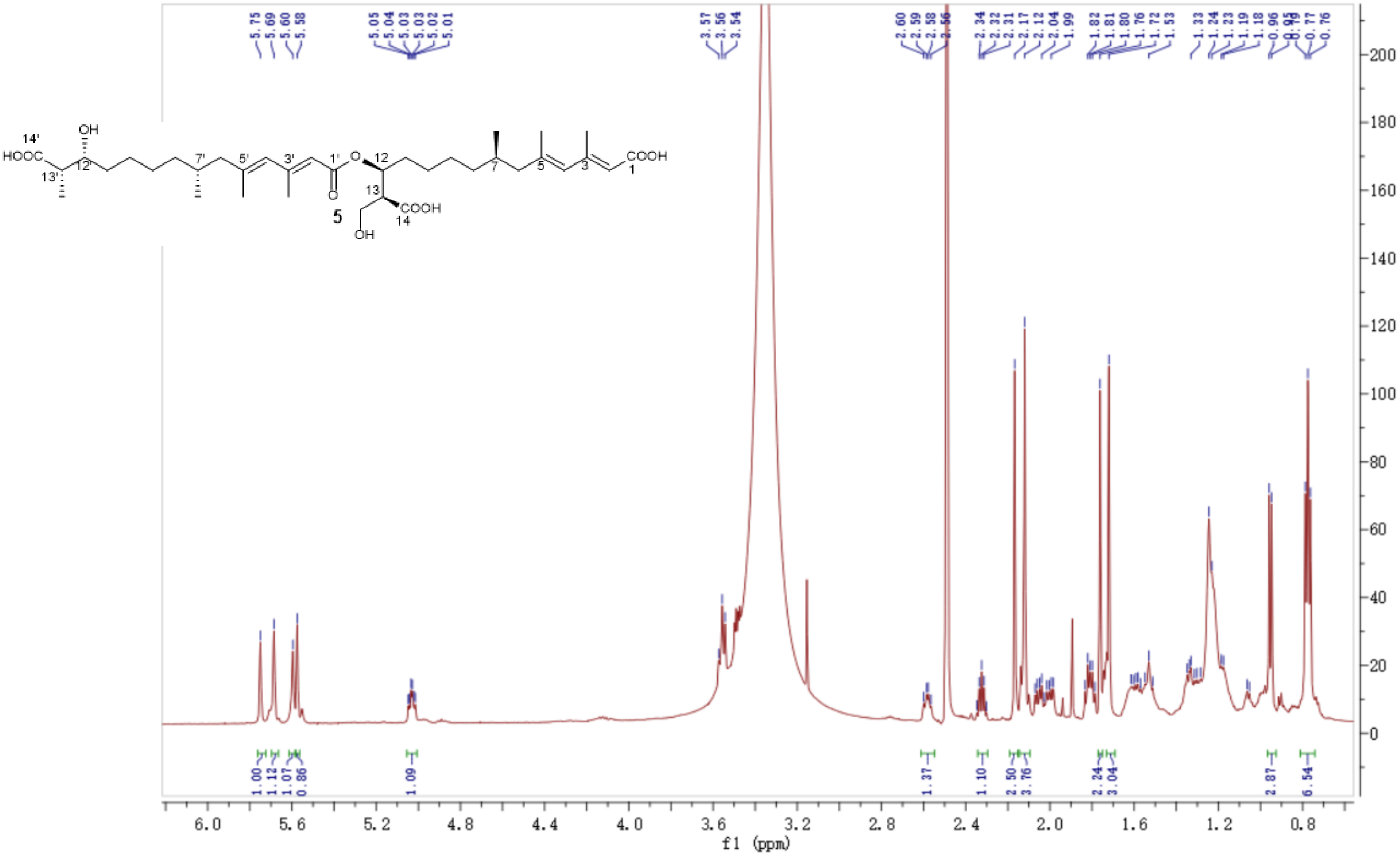

Figure S24. ^{1}H-NMR (DMSO-d_6, 600 MHz) of compound **5**.

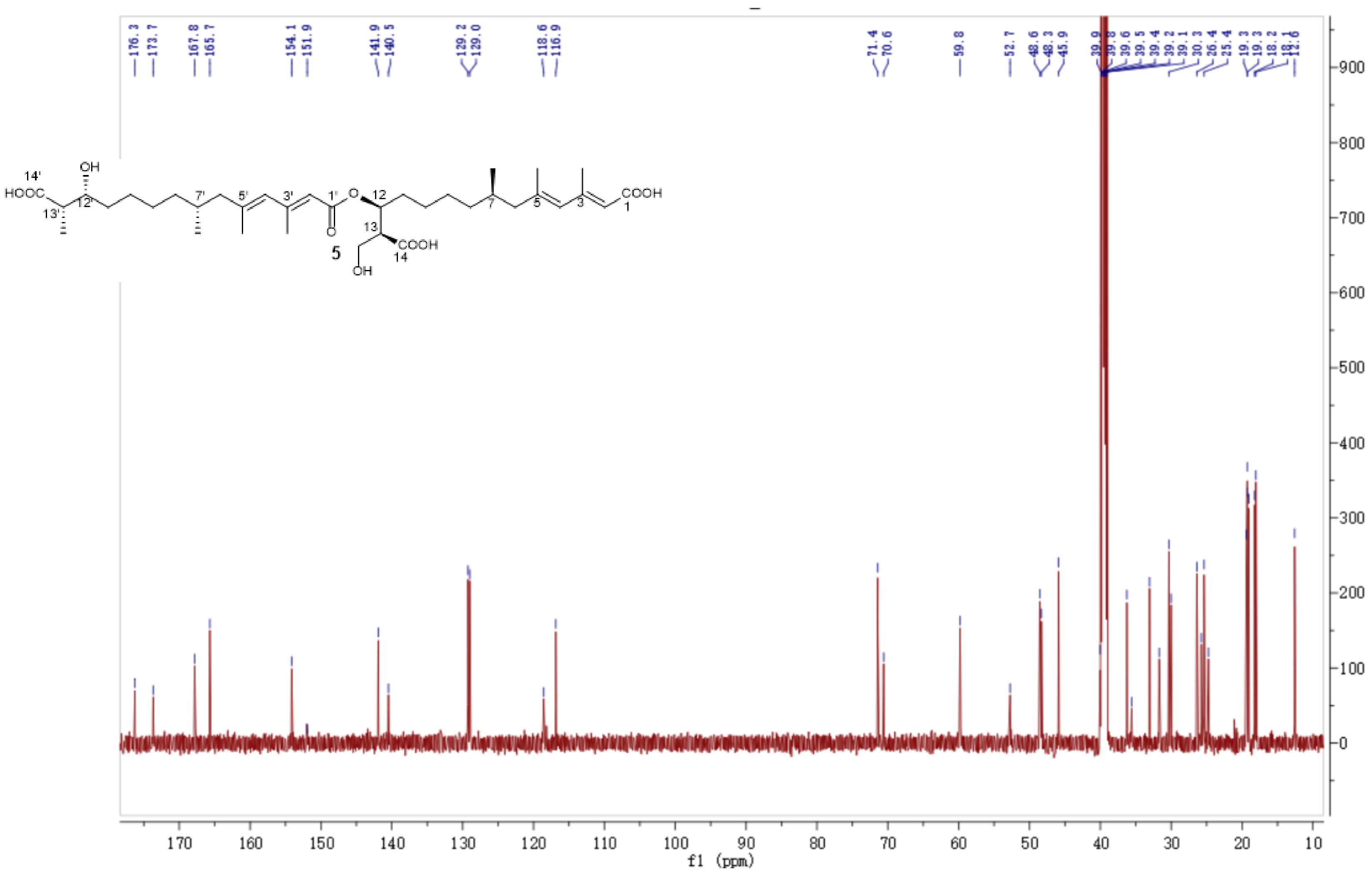

Figure S25. ^{13}C-NMR (DMSO-d_6, 150 MHz) of compound **5**.

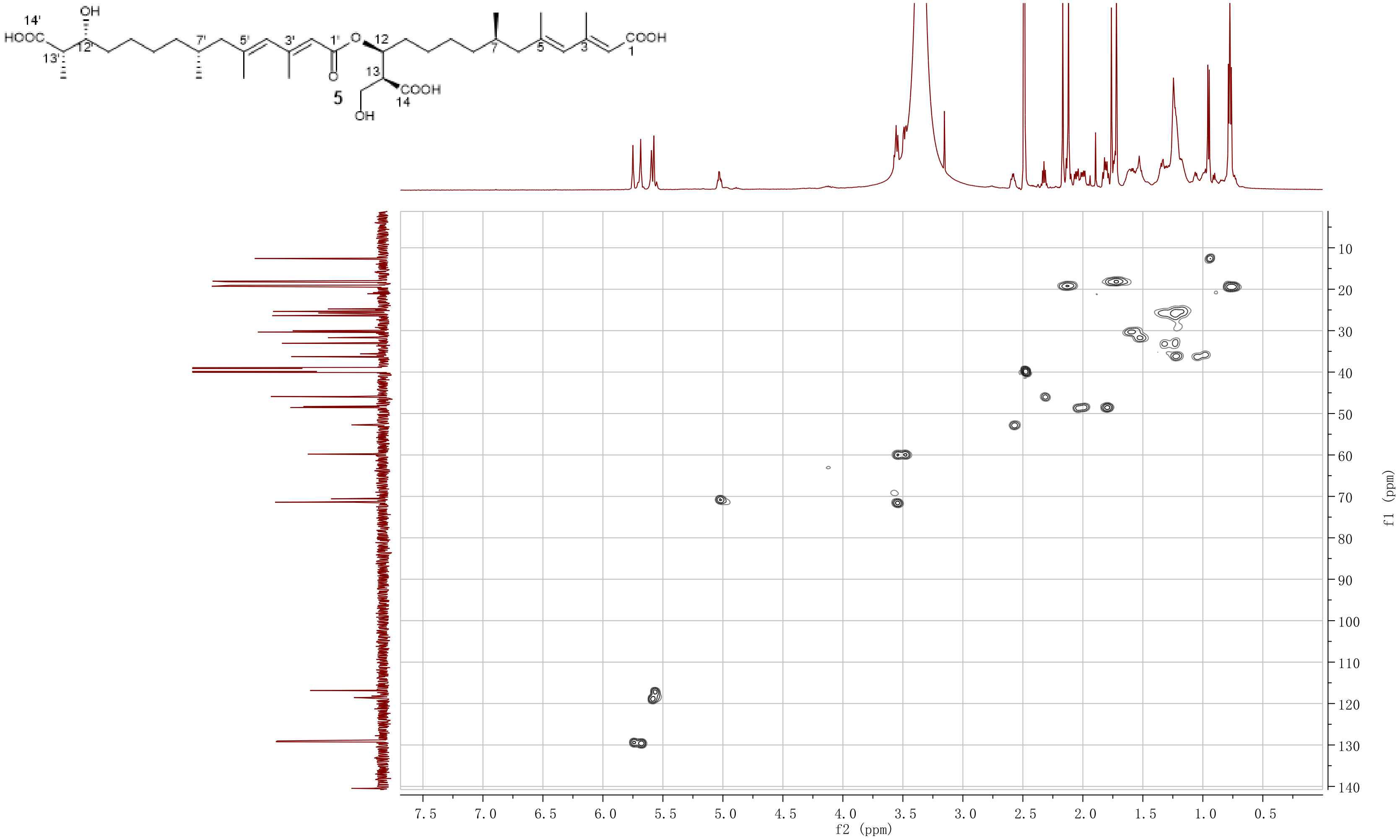

Figure S26. HSQC (DMSO-d_6, 600 MHz) of Compound **5**.

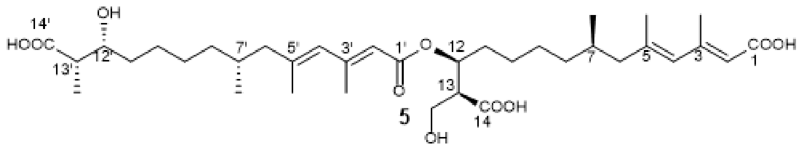

5

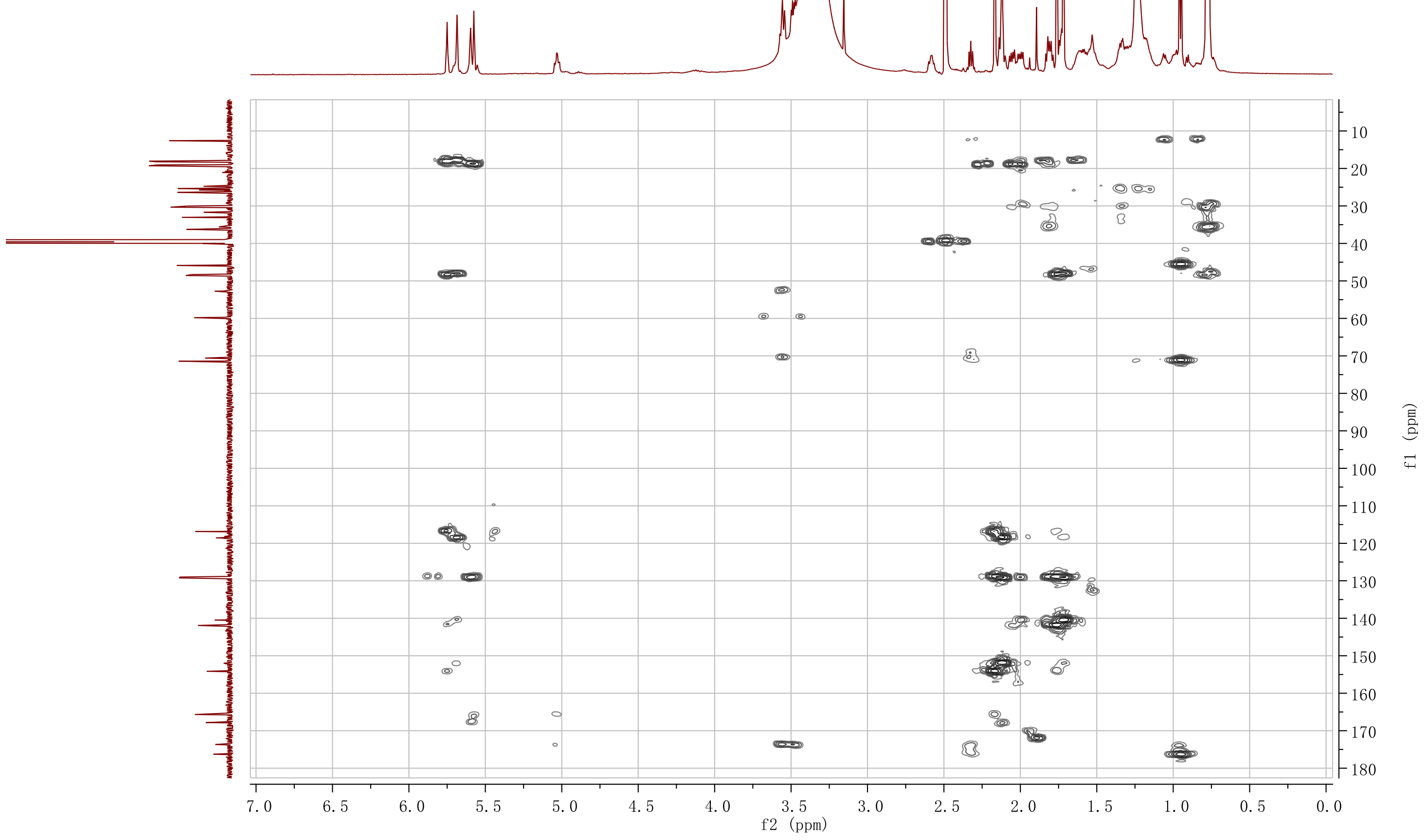

Figure S27. HMBC (DMSO-d_6, 600 MHz) of compound **5**.

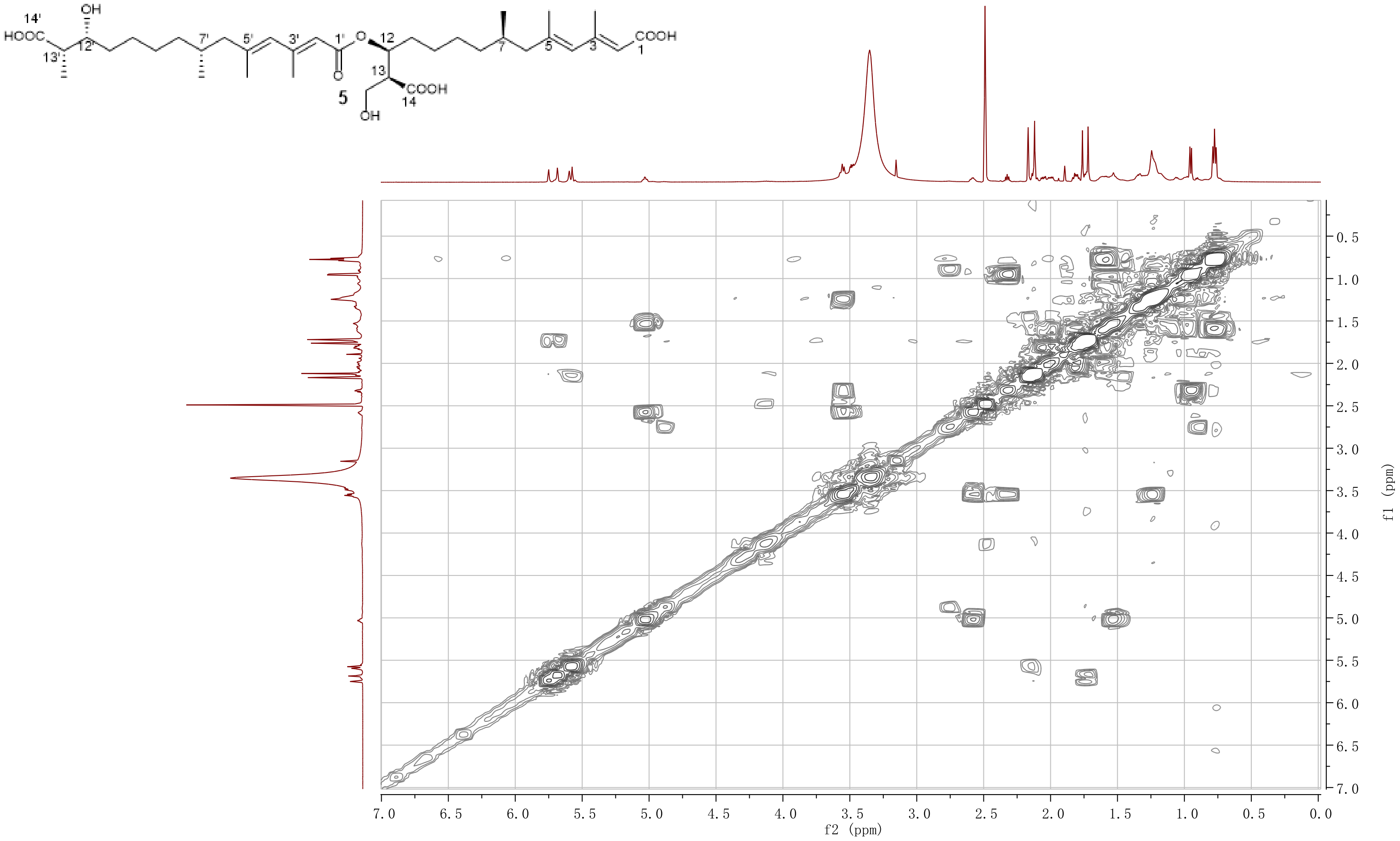

Figure S28. COSY (DMSO-d_6, 600 MHz) of compound **5**.

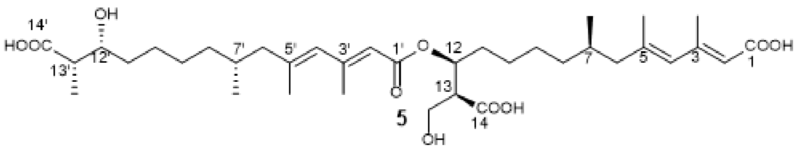

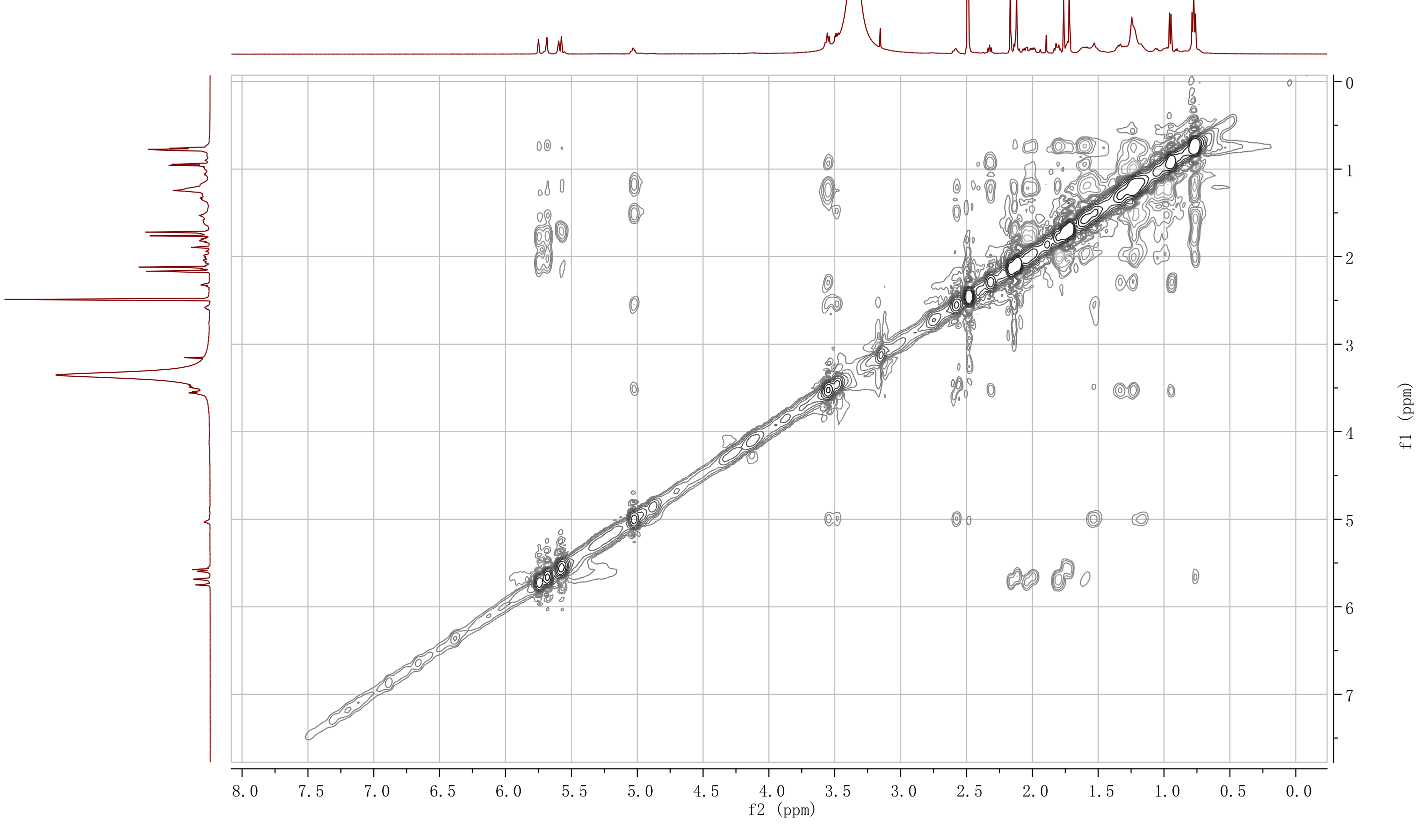

Figure S29. ROESY (DMSO-d_6, 600 MHz) of compound **5**.

Data File: E:\DATA\2020\0929\int20-10.lcd

Elmt	Val.	Min	Max	Elmt	Val.	Min	Max	Elmt	Val.	Min	Max	Elmt	Val.	Min	Max	Use Adduct
H	1	10	110	F	1	0	0	S	2	0	0	Br	1	0	0	H
2H	1	0	0	Na	1	0	0	Cl	1	0	0	Pd	2	0	0	
C	4	5	50	Mg	2	0	0	Co	2	0	0	Ag	1	0	0	
N	3	0	10	Si	4	0	0	Cu	2	0	0	I	3	0	0	
O	2	0	40	P	3	0	0	Se	2	0	0					

Error Margin (ppm): 5
HC Ratio: unlimited
Max Isotopes: all
MSn Iso RI (%): 75.00

DBE Range: -2.0 - 100.0
Apply N Rule: yes
Isotope RI (%): 1.00
MSn Logic Mode: OR

Electron Ions: both
Use MSn Info: yes
Isotope Res: 10000
Max Results: 10

Event#: 2 MS(E-) Ret. Time : 0.373 -> 0.387 Scan# : 58 -> 60

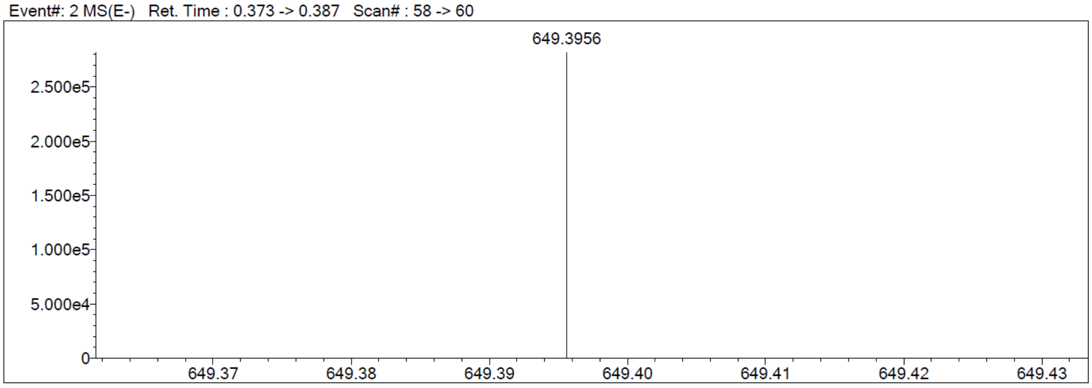

Measured region for 649.3956 m/z

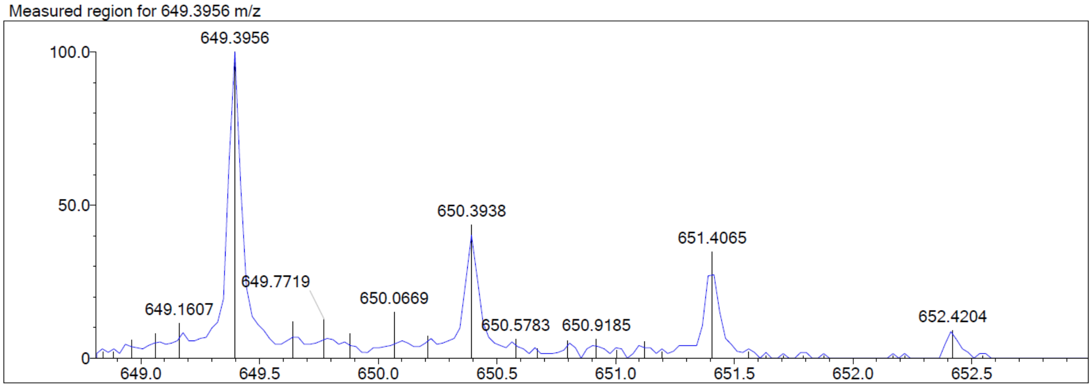

C36 H58 O10 [M-H]- : Predicted region for 649.3957 m/z

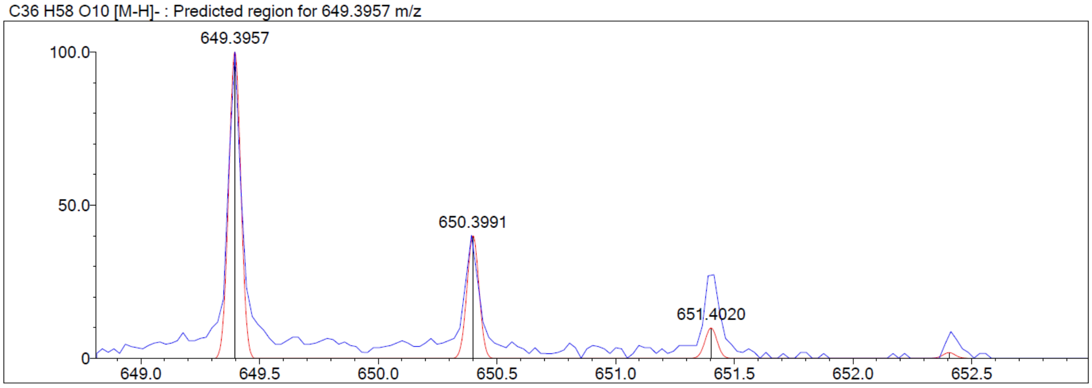

Formula (M)	Ion	Meas. m/z	Pred. m/z	Df. (mDa)	Df. (ppm)	DBE
C36 H58 O10	[M-H]-	649.3956	649.3957	-0.1	-0.15	8.0

Figure S30. HR-ESI-MS spectrum of **5**.

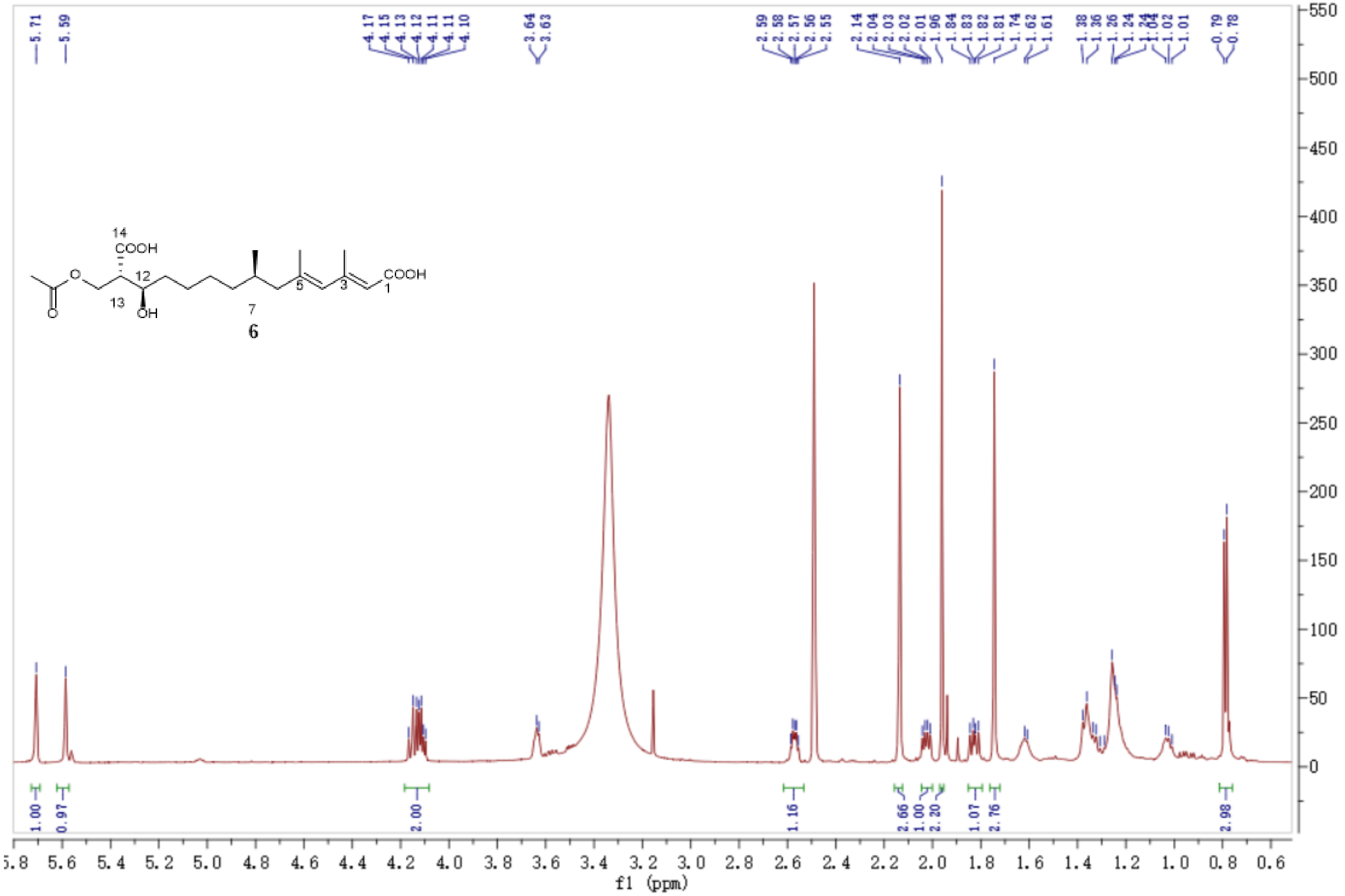

Figure S31. ^{1}H-NMR (DMSO-d_6, 600 MHz) of compound **6**.

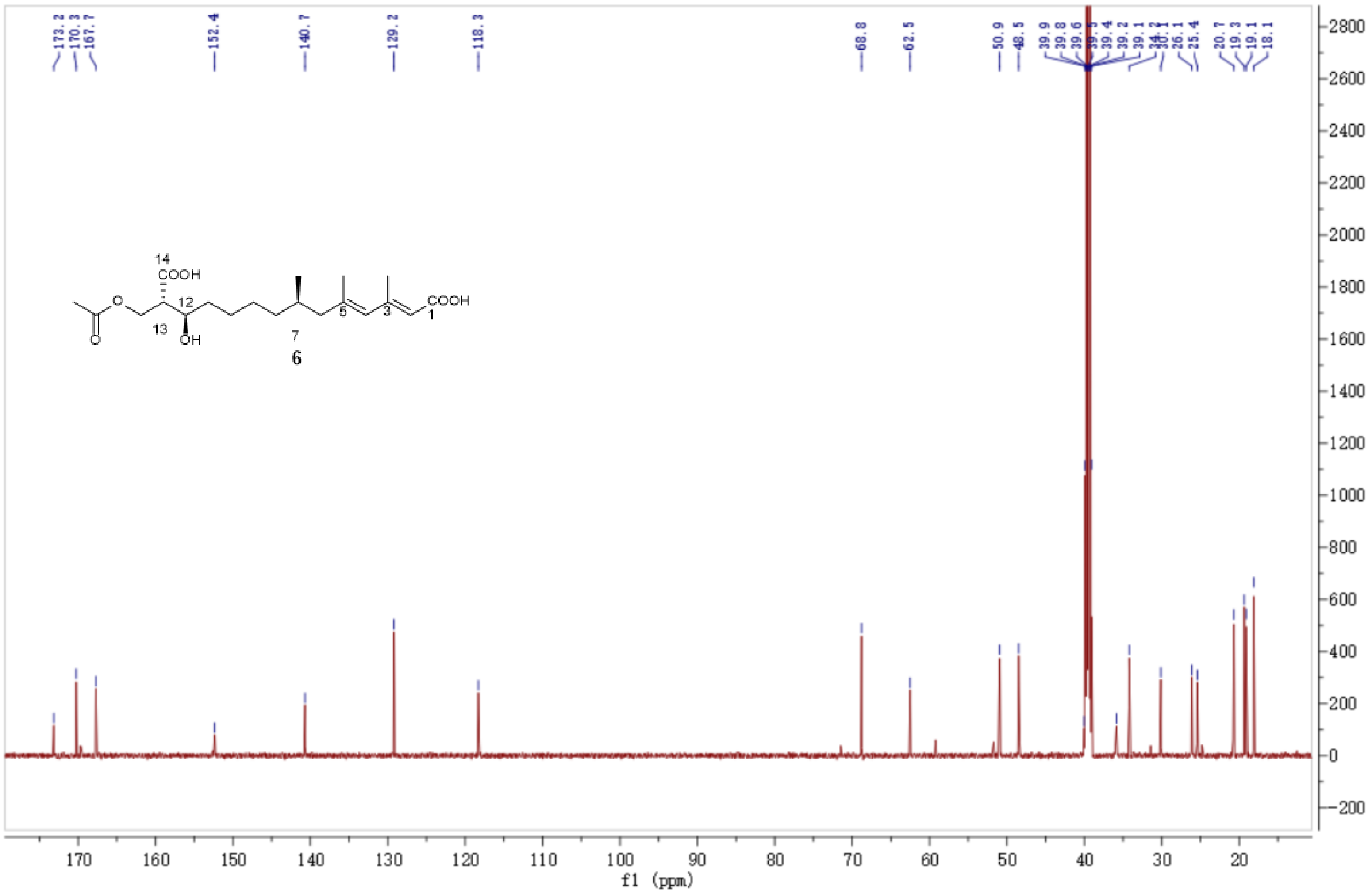

Figure S32. ^{13}C-NMR (DMSO-d_6, 150 MHz) of compound **6**.

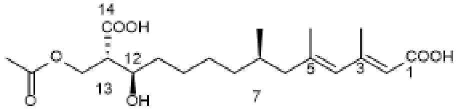

6

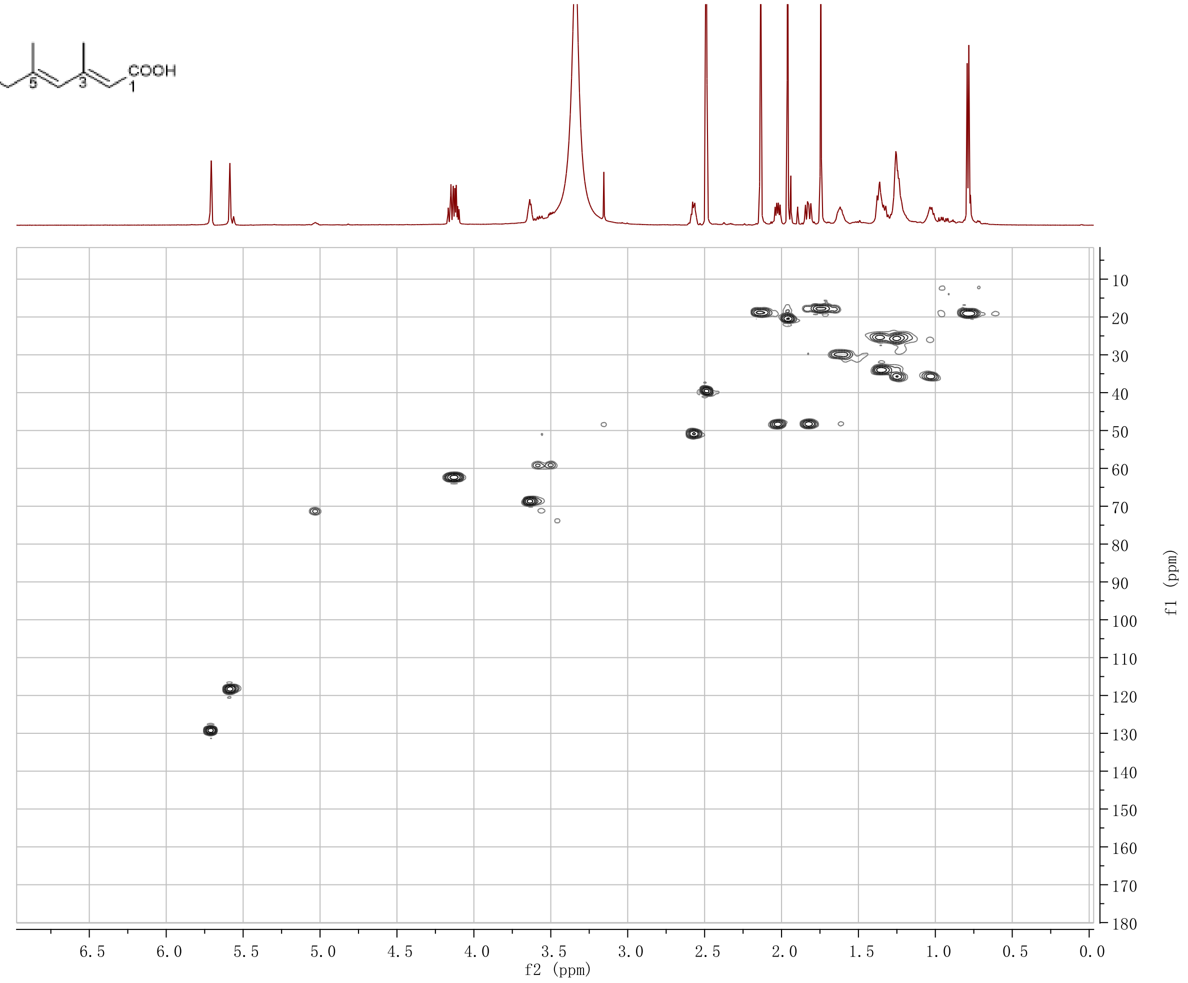

Figure S33. HSQC (DMSO-d_6, 600 MHz) of Compound **6**.

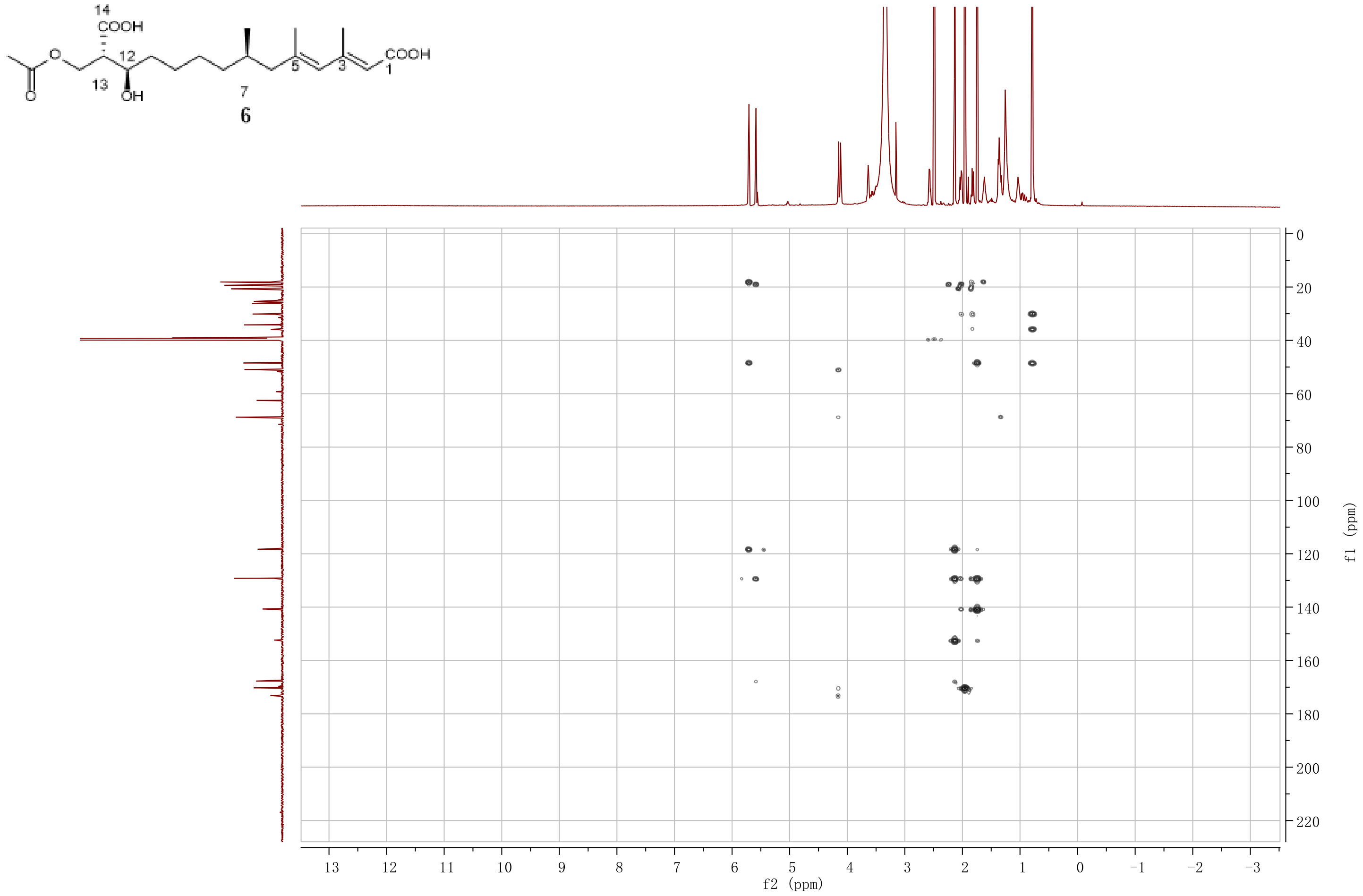

Figure S34. HMBC (DMSO-d_6, 600 MHz) of compound **6**.

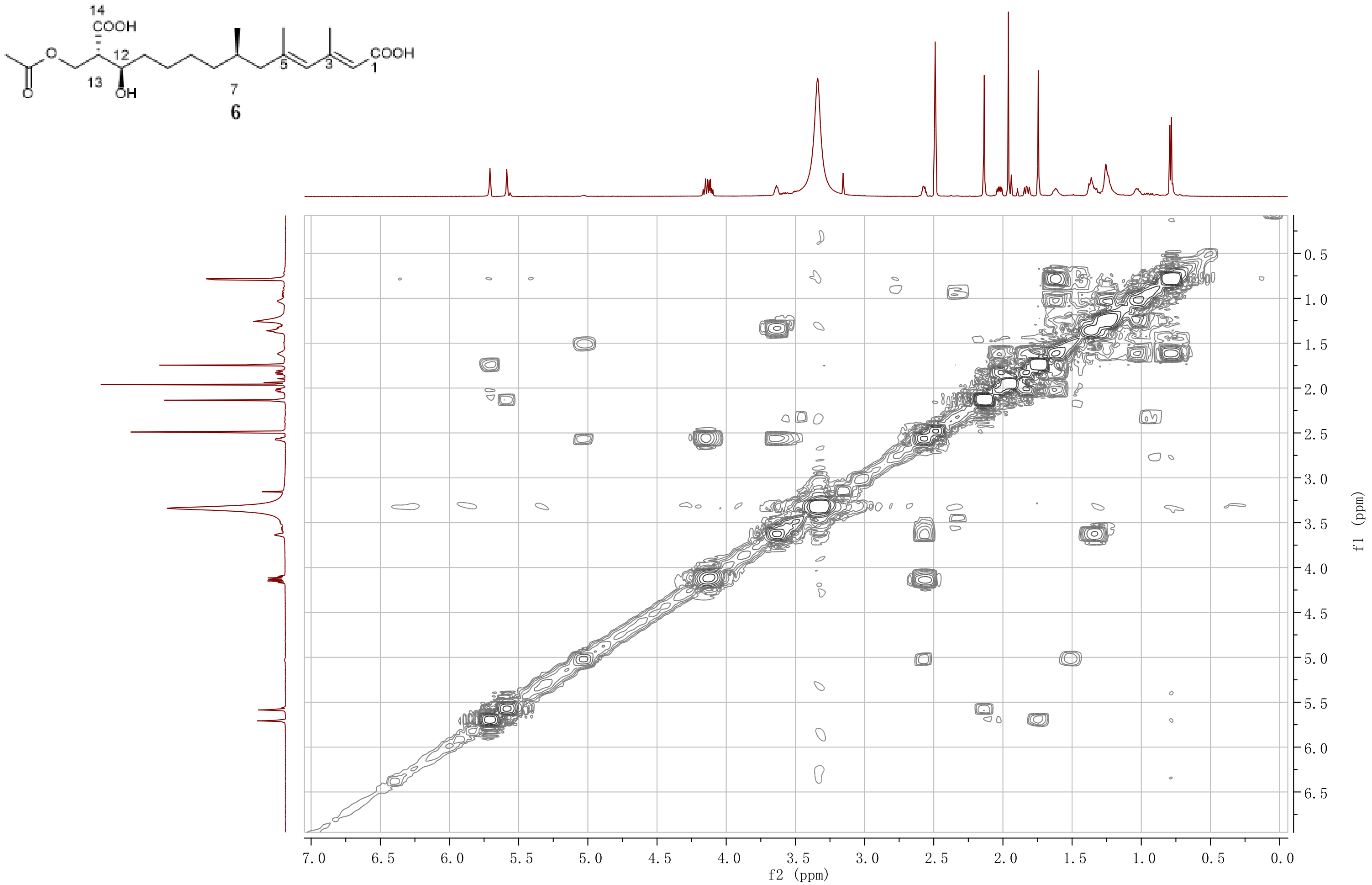

Figure S35. COSY (DMSO-d_6, 600 MHz) of compound **6**.

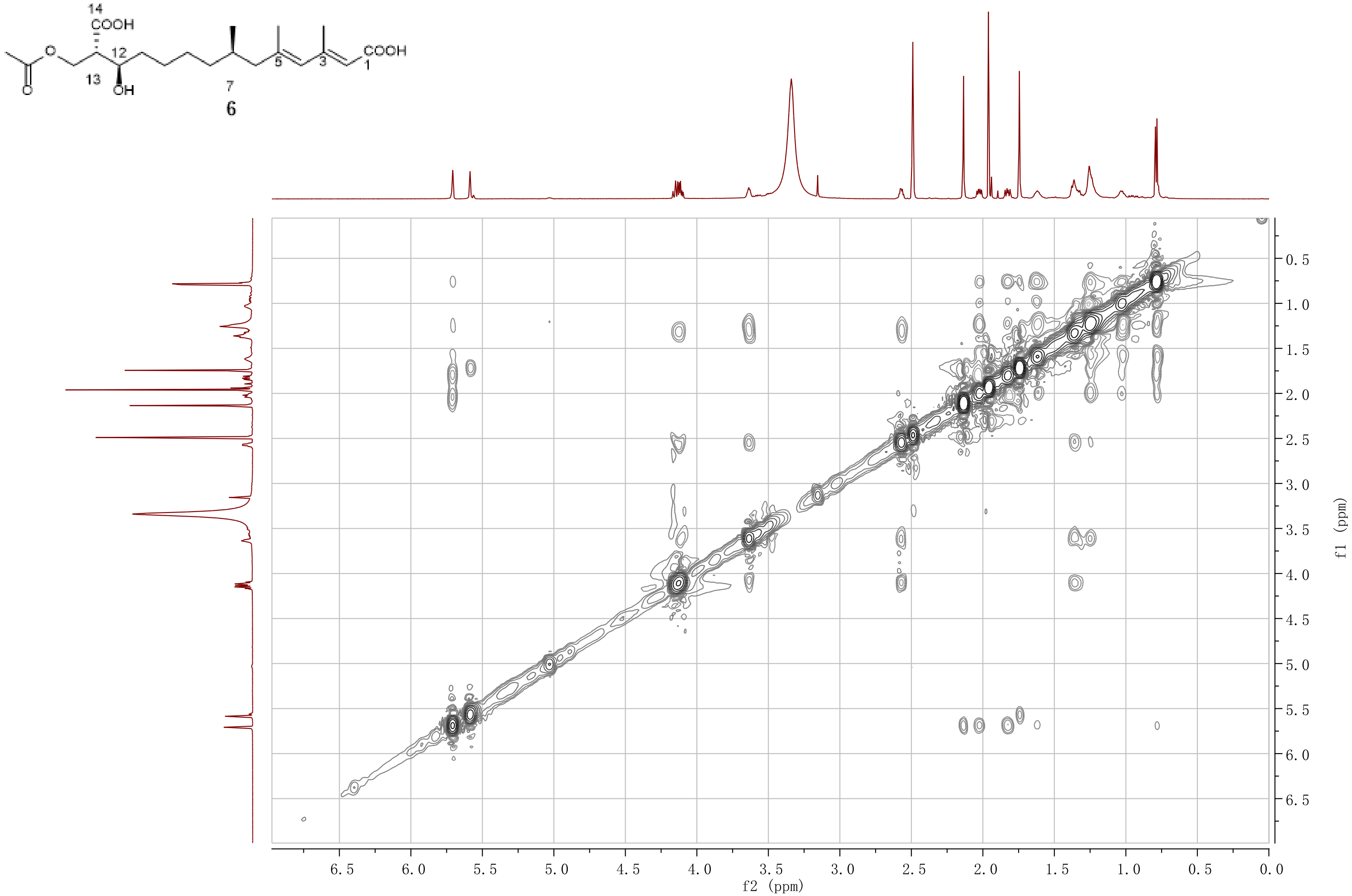

Figure S36. ROESY (DMSO-d_6, 600 MHz) of compound **6**.

Data File: E:\DATA\2020\0925\HSX-int20-5.lcd

Elmt	Val.	Min	Max	Elmt	Val.	Min	Max	Elmt	Val.	Min	Max	Elmt	Val.	Min	Max	Use Adduct
H	1	10	110	F	1	0	0	S	2	0	0	Br	1	0	0	H
2H	1	0	0	Na	1	0	0	Cl	1	0	0	Pd	2	0	0	
C	4	5	50	Mg	2	0	0	Co	2	0	0	Ag	1	0	0	
N	3	0	10	Si	4	0	0	Cu	2	0	0	I	3	0	0	
O	2	0	40	P	3	0	0	Se	2	0	0					

Error Margin (ppm):	5	DBE Range:	-2.0 - 100.0	Electron Ions:	both
HC Ratio:	unlimited	Apply N Rule:	yes	Use MSn Info:	yes
Max Isotopes:	all	Isotope RI (%):	1.00	Isotope Res:	10000
MSn Iso RI (%):	75.00	MSn Logic Mode:	OR	Max Results:	10

Event#: 2 MS(E-) Ret. Time : 0.427 Scan# : 66

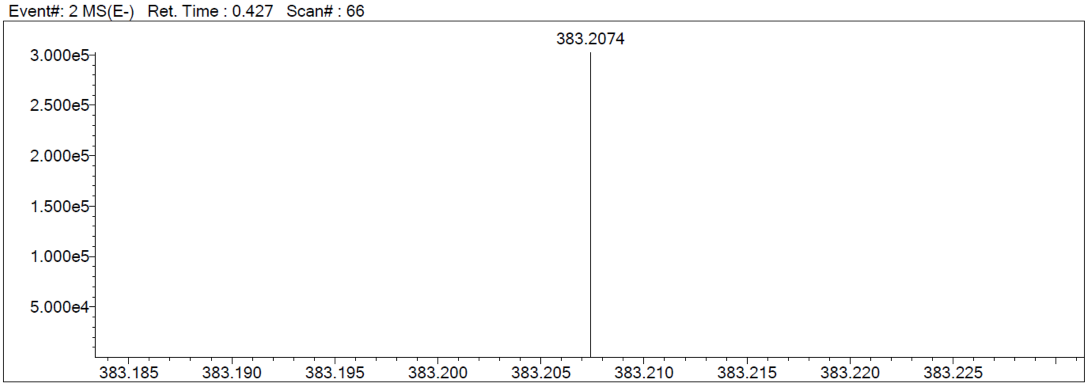

Measured region for 383.2074 m/z

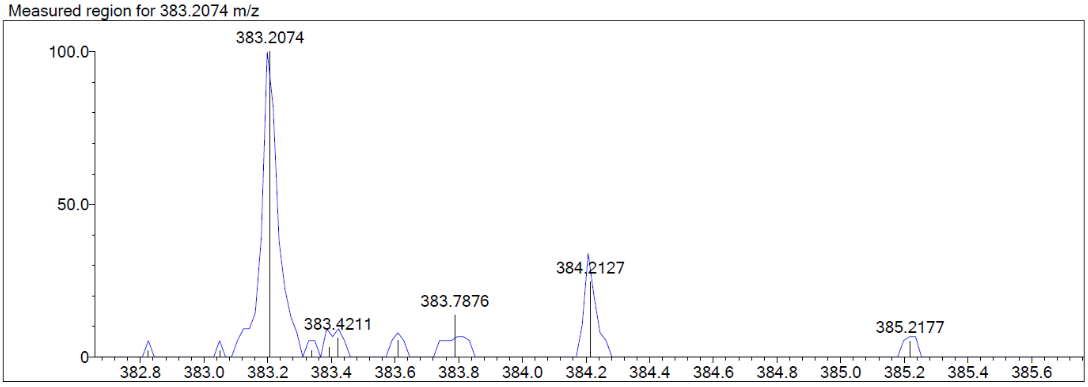

C20 H32 O7 [M-H]- : Predicted region for 383.2075 m/z

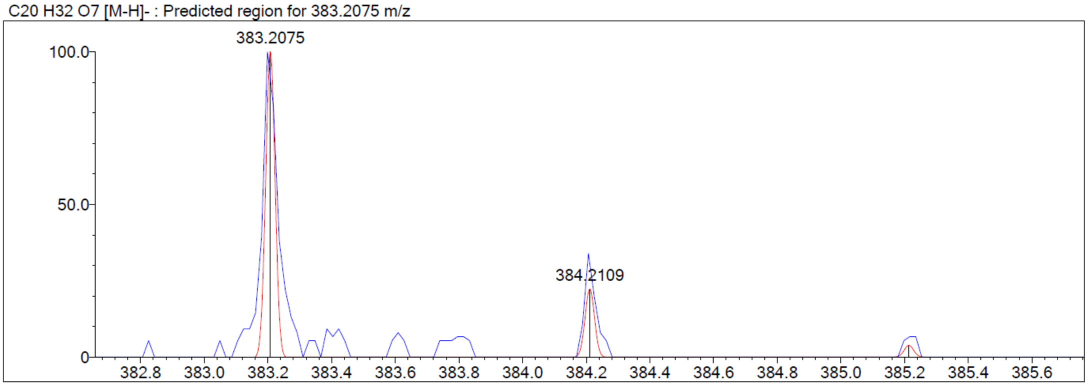

Formula (M)	Ion	Meas. m/z	Pred. m/z	Df. (mDa)	Df. (ppm)	DBE
C20 H32 O7	[M-H]-	383.2074	383.2075	-0.1	-0.26	5.0

Figure S37. HR-ESI-MS spectrum of **6**.

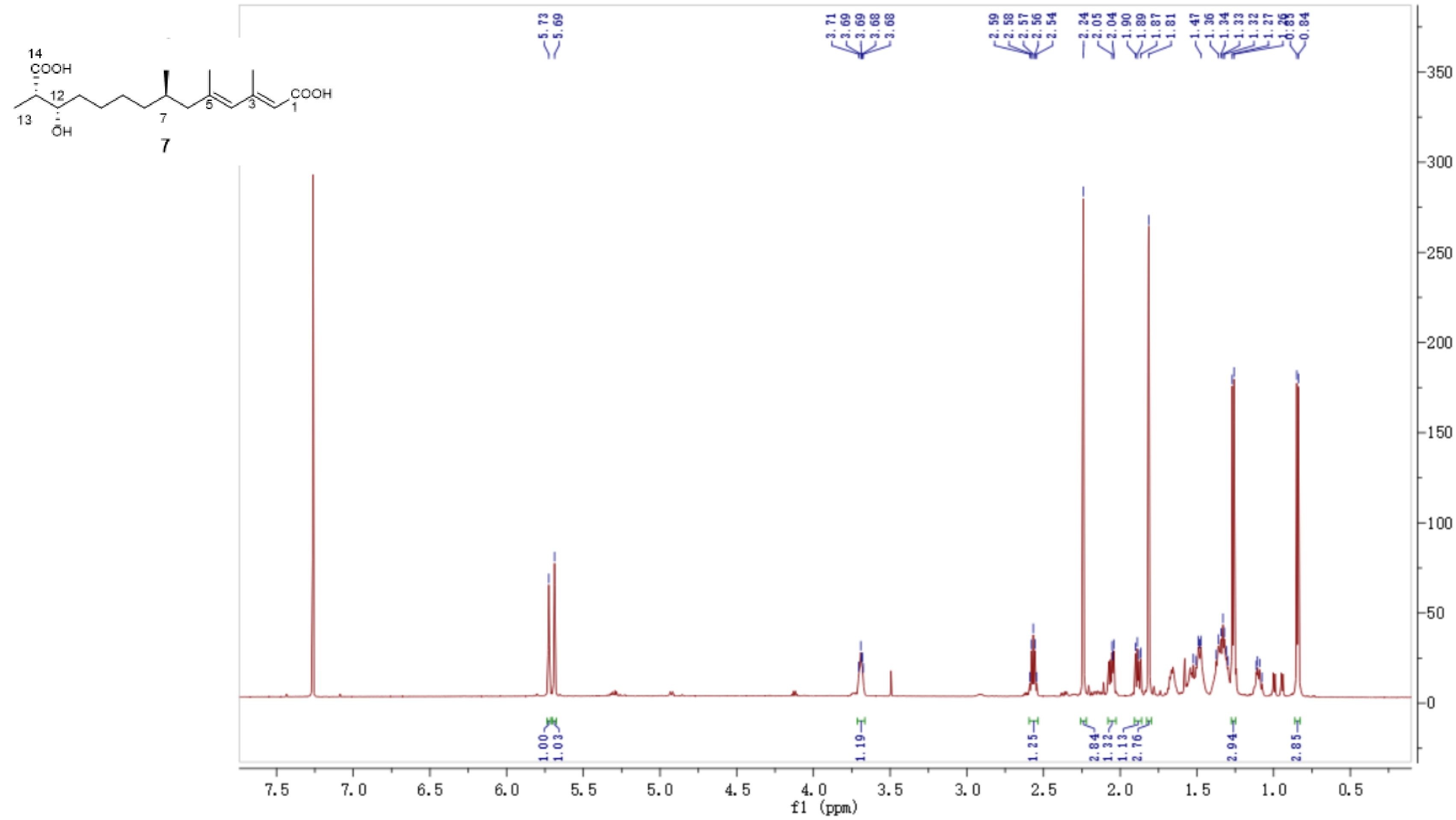

Figure S38. ^{1}H-NMR ($CDCl_3$, 600 MHz) of compound **7**.

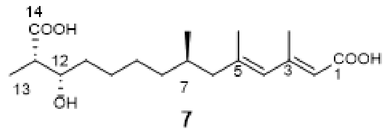

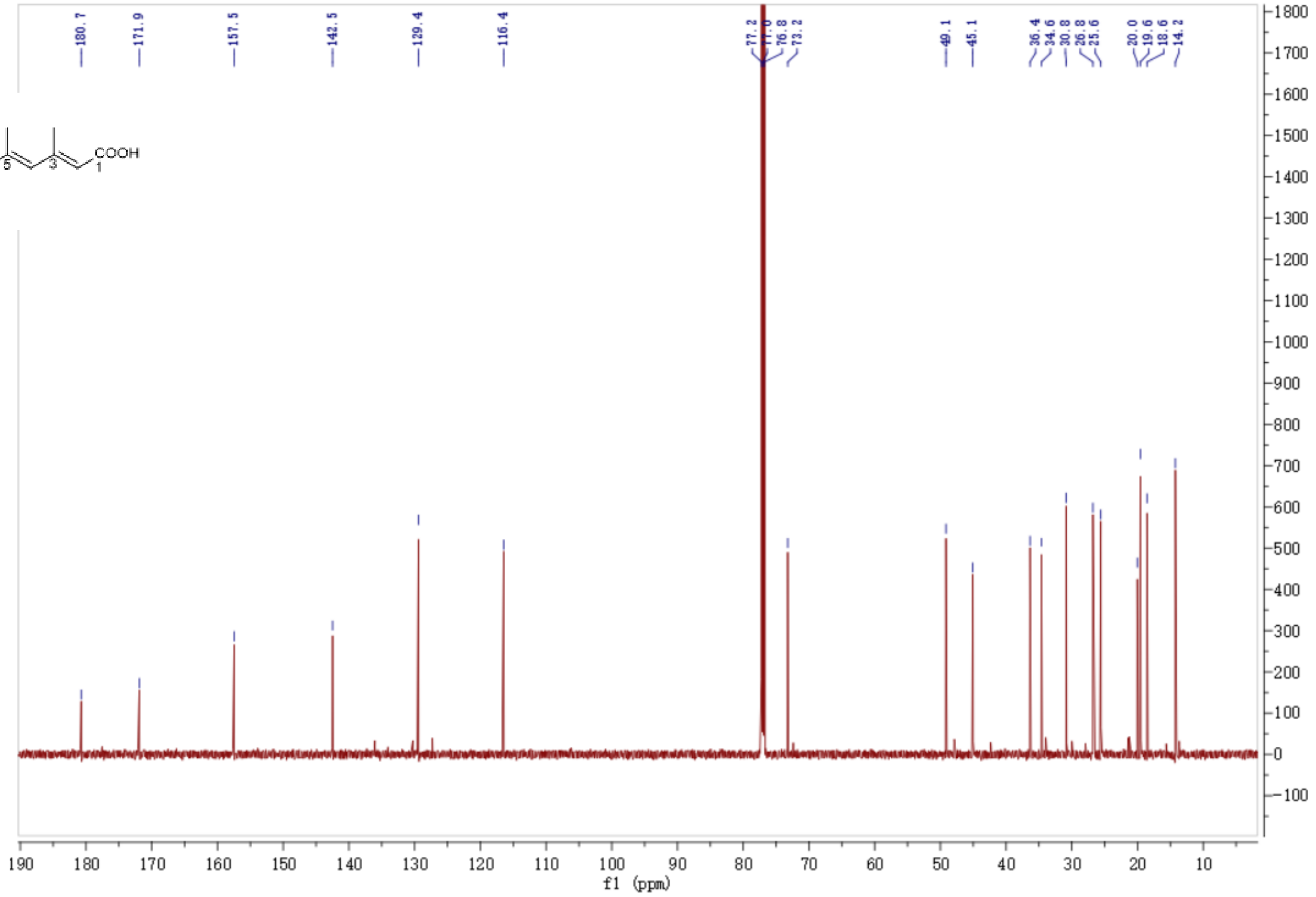

Figure S39. ^{13}C-NMR ($CDCl_3$, 150 MHz) of compound **7**.

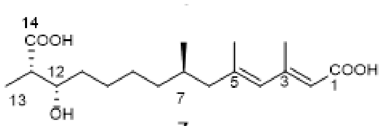

7

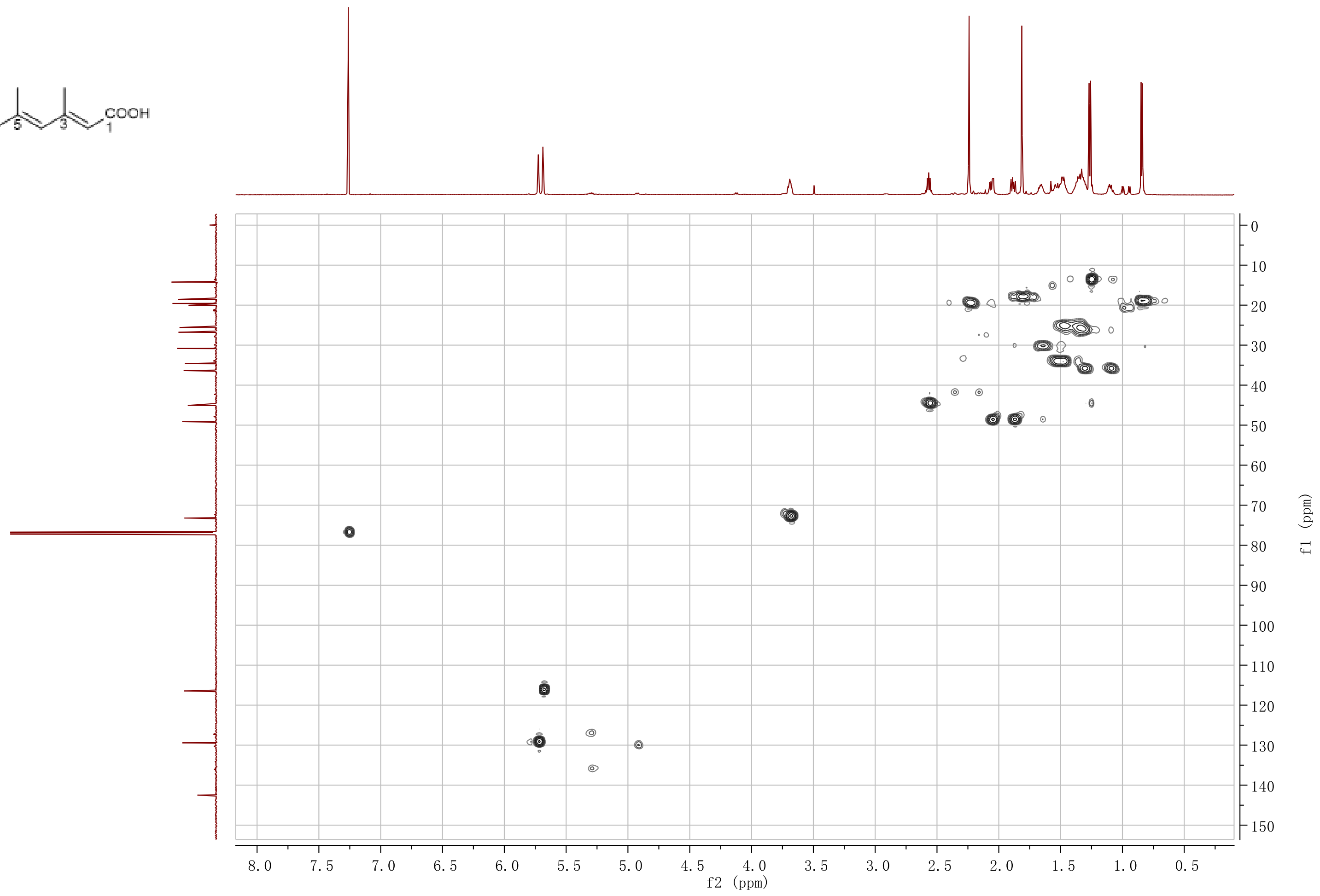

Figure S40. HSQC ($CDCl_3$, 600 MHz) of Compound **7**.

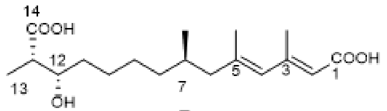

7

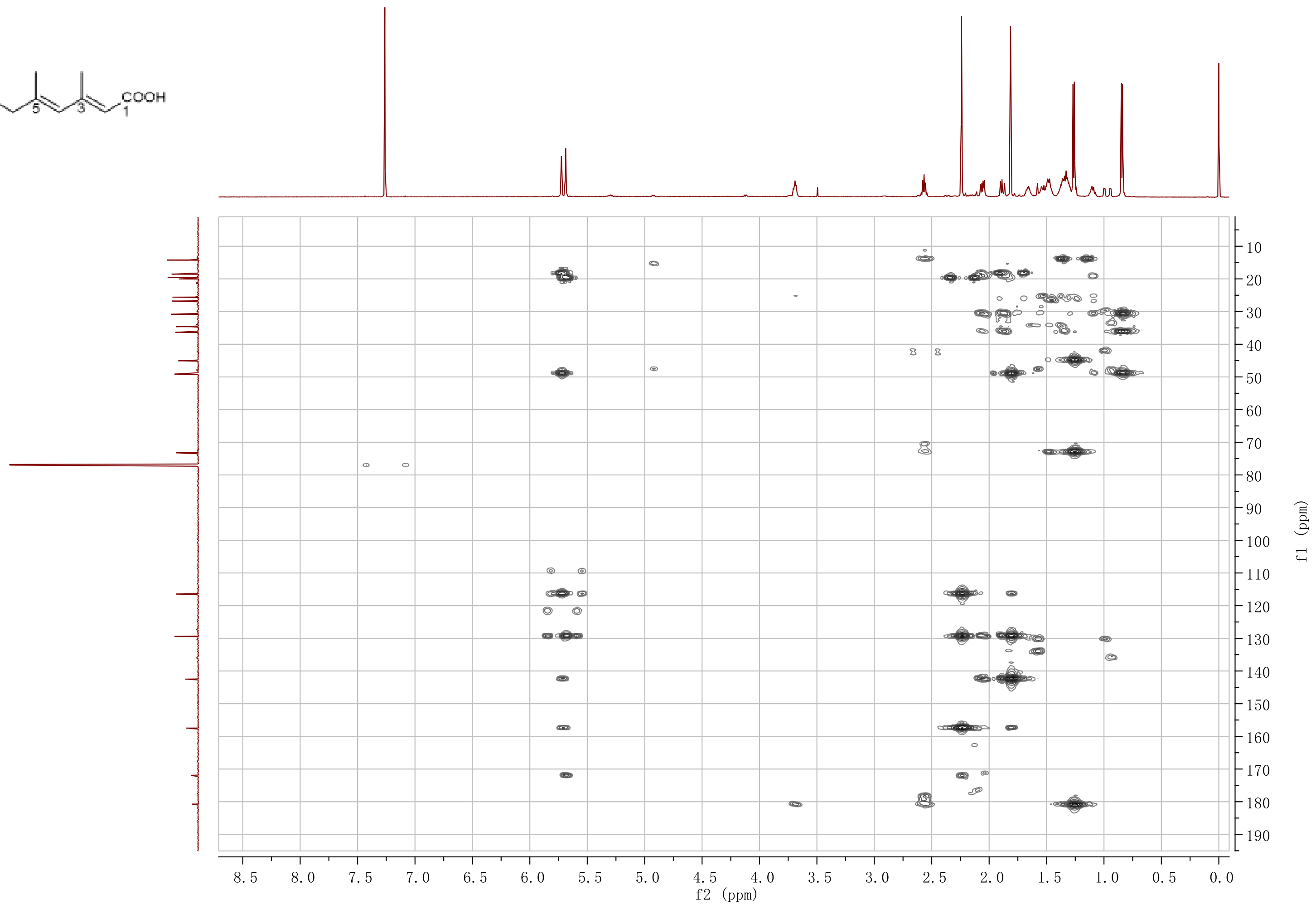

Figure S41. HMBC ($CDCl_3$, 600 MHz) of compound **7**.

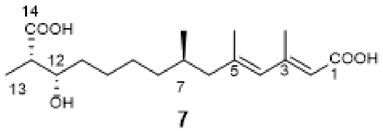

7

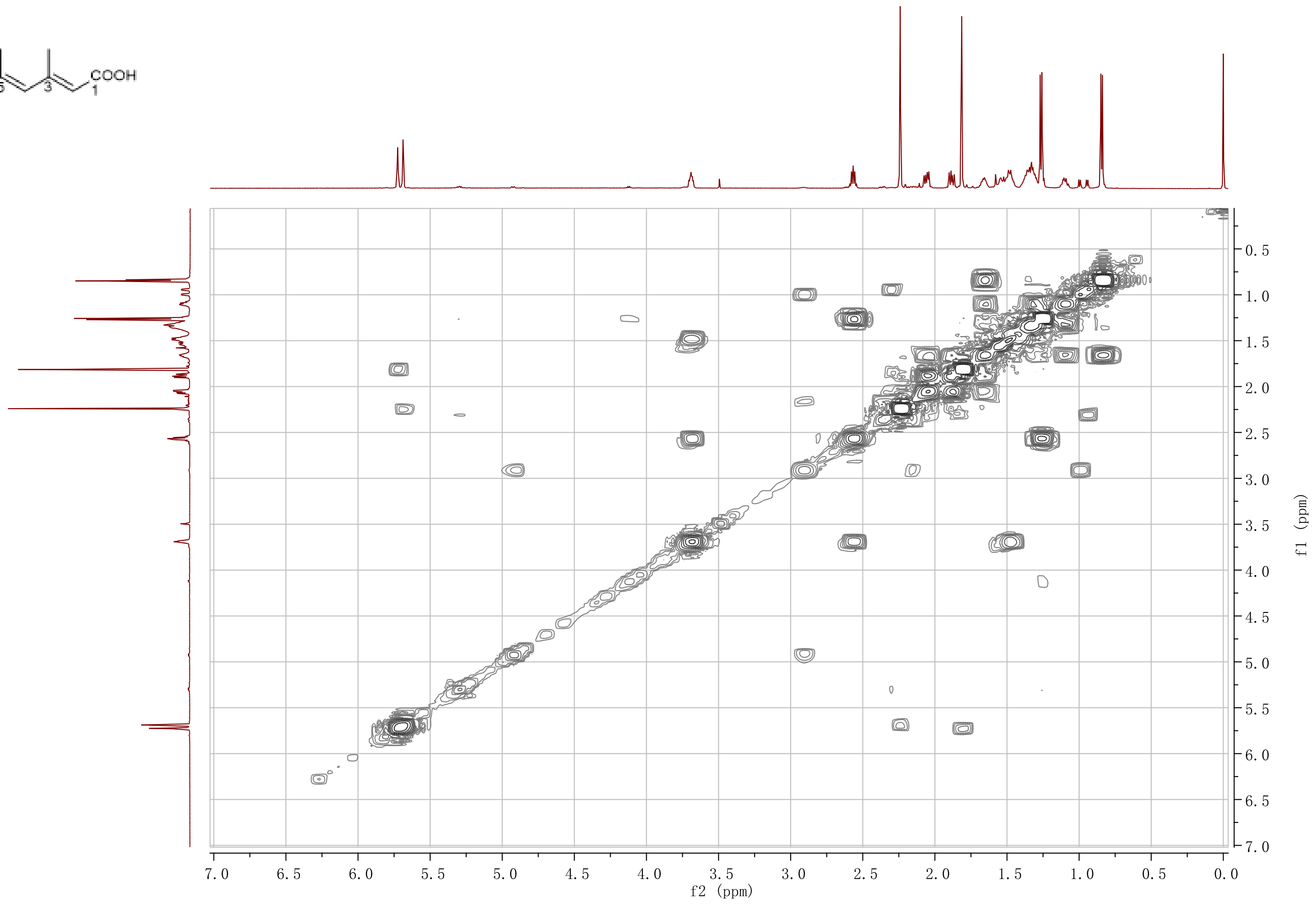

Figure S42. COSY ($CDCl_3$, 600 MHz) of compound **7**.

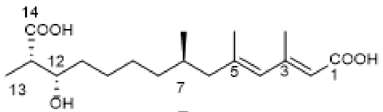

7

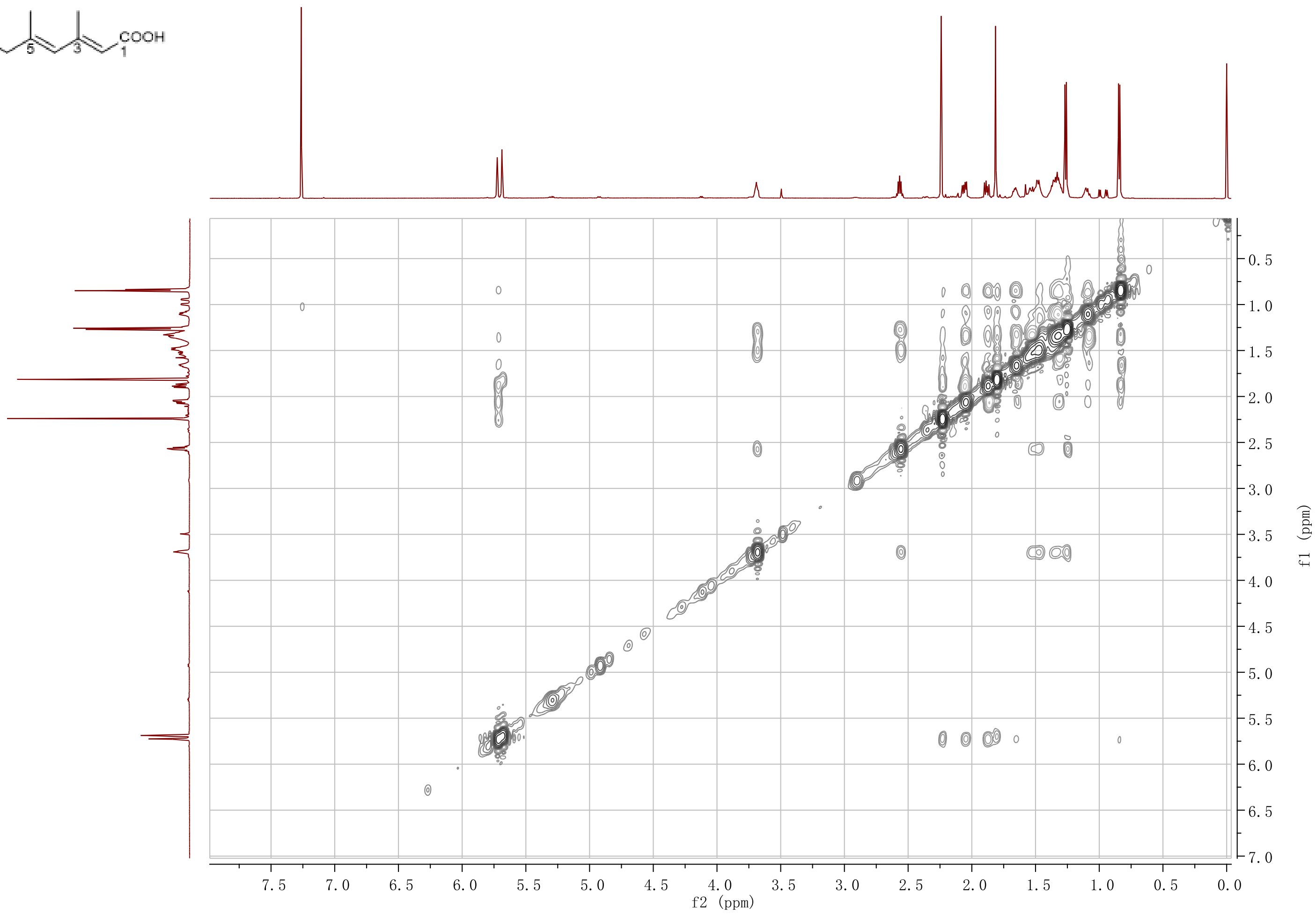

Figure S43. ROESY ($CDCl_3$, 600 MHz) of compound **7**.

Data File: E:\DATA\2020\1216\int20-21a.lcd

Elmt	Val.	Min	Max	Elmt	Val.	Min	Max	Elmt	Val.	Min	Max	Elmt	Val.	Min	Max	Use Adduct
H	1	5	100	F	1	0	0	S	2	0	5	Br	1	0	0	H
2H	1	0	0	Na	1	0	0	Cl	1	0	0	Pd	2	0	0	
C	4	5	50	Mg	2	0	0	Co	2	0	0	Ag	1	0	0	
N	3	0	10	Si	4	0	5	Cu	2	0	0	I	3	0	0	
O	2	0	30	P	3	0	0	Se	2	0	0					

Error Margin (ppm): 5
HC Ratio: unlimited
Max Isotopes: all
MSn Iso RI (%): 75.00

DBE Range: -2.0 - 100.0
Apply N Rule: yes
Isotope RI (%): 1.00
MSn Logic Mode: OR

Electron Ions: both
Use MSn Info: yes
Isotope Res: 10000
Max Results: 10

Event#: 2 MS(E-) Ret. Time : 0.347 -> 0.440 Scan# : 54 -> 68

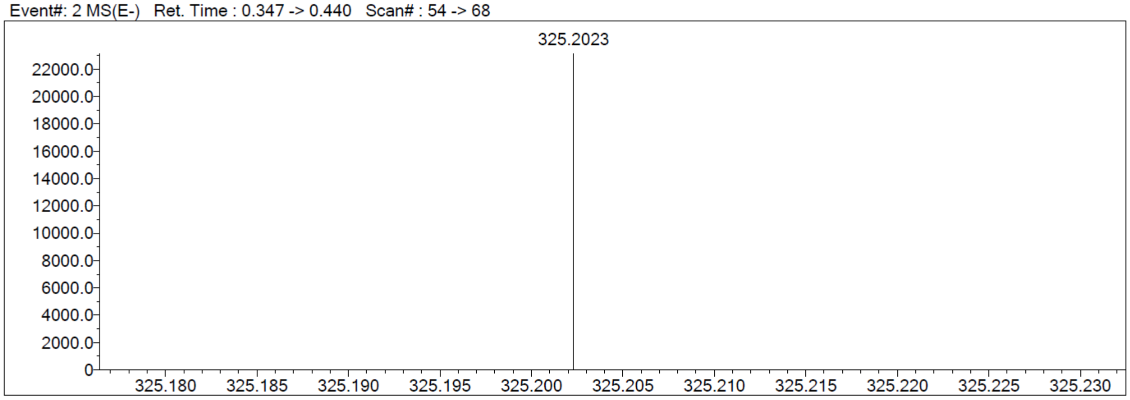

Measured region for 325.2023 m/z

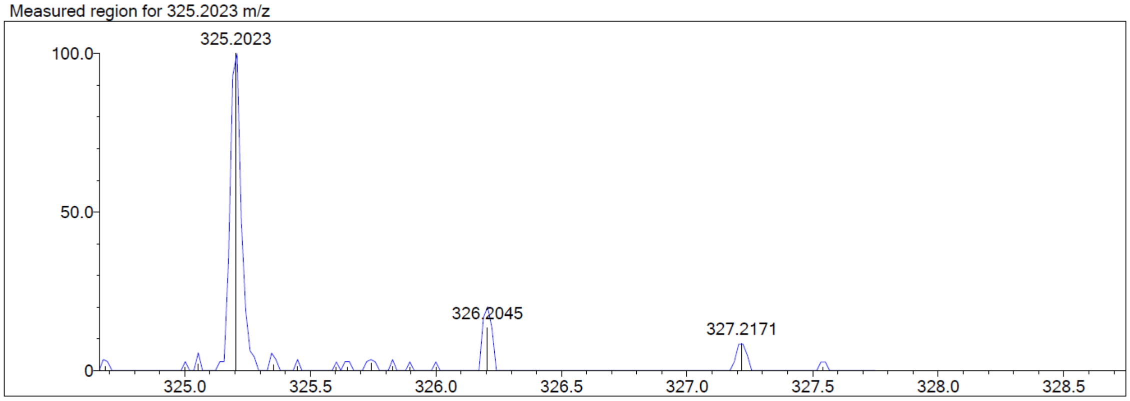

C18 H30 O5 [M-H]- : Predicted region for 325.2020 m/z

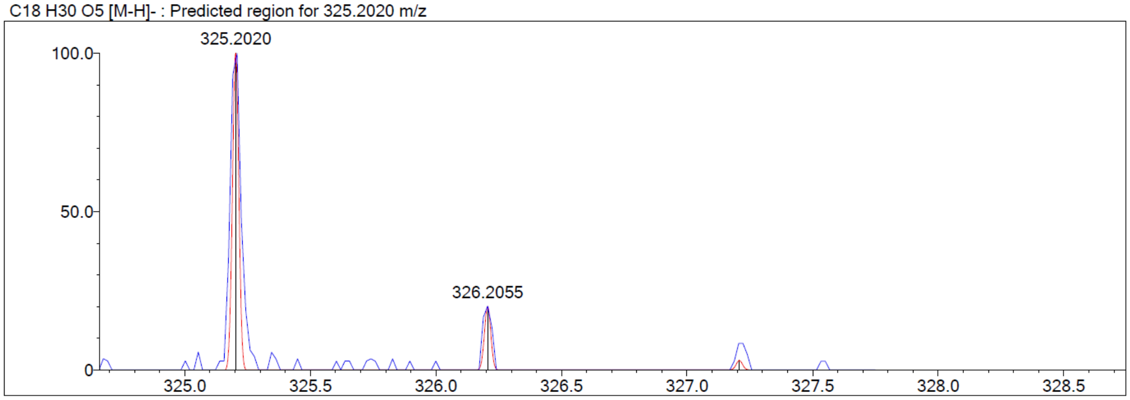

Formula (M)	Ion	Meas. m/z	Pred. m/z	Df. (mDa)	Df. (ppm)	DBE
C18 H30 O5	[M-H]-	325.2023	325.2020	0.3	0.92	4.0

Figure S44. HR-ESI-MS spectrum of **7**.

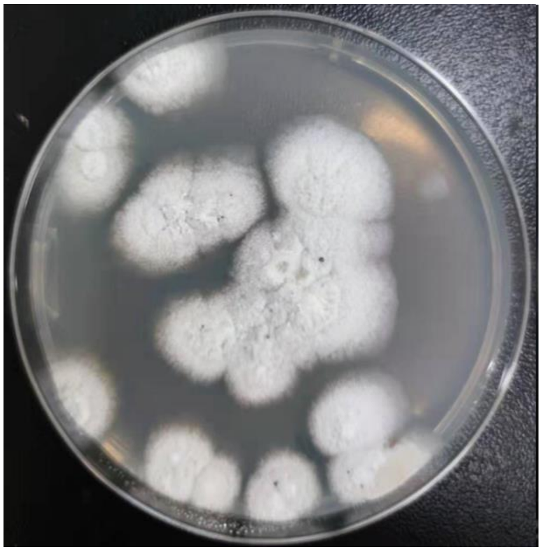

Figure S45. The morphology of *Scopulariopsis candelabrum* KIB-int20.

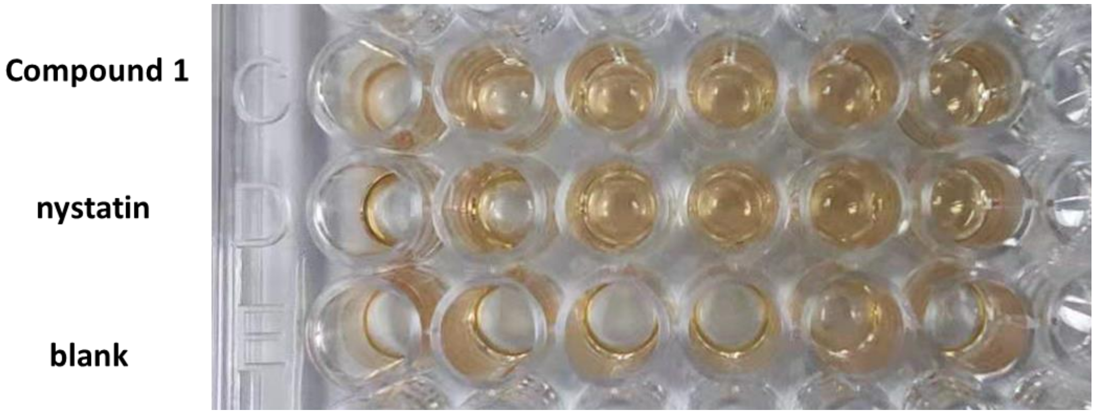

Figure S46. Broth dilution antifungal susceptibility testing of *Candida albicans*.

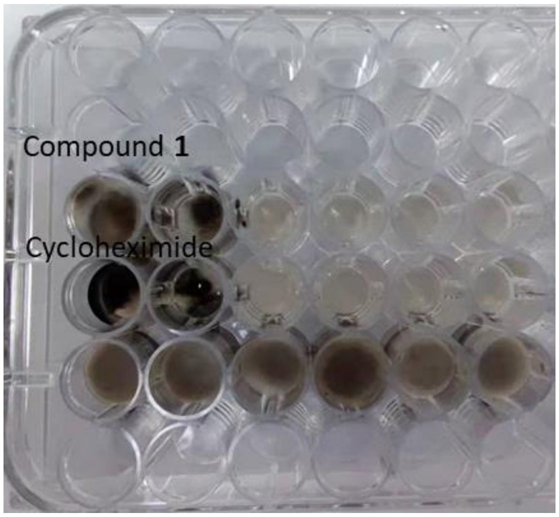

Figure S47. Inhibiting the growth of mycelial method testing of *Exserohilum turcicum*.

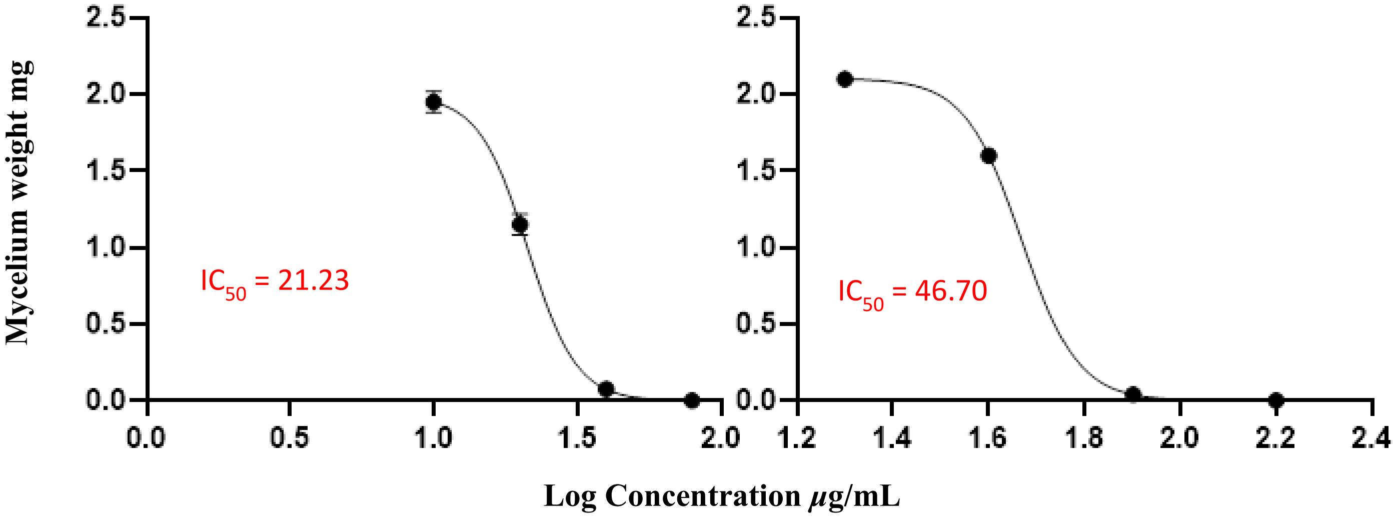

Figure S48. Compound **1** (left) and cycloheximide (right) inhibiting the growth of mycelial line.

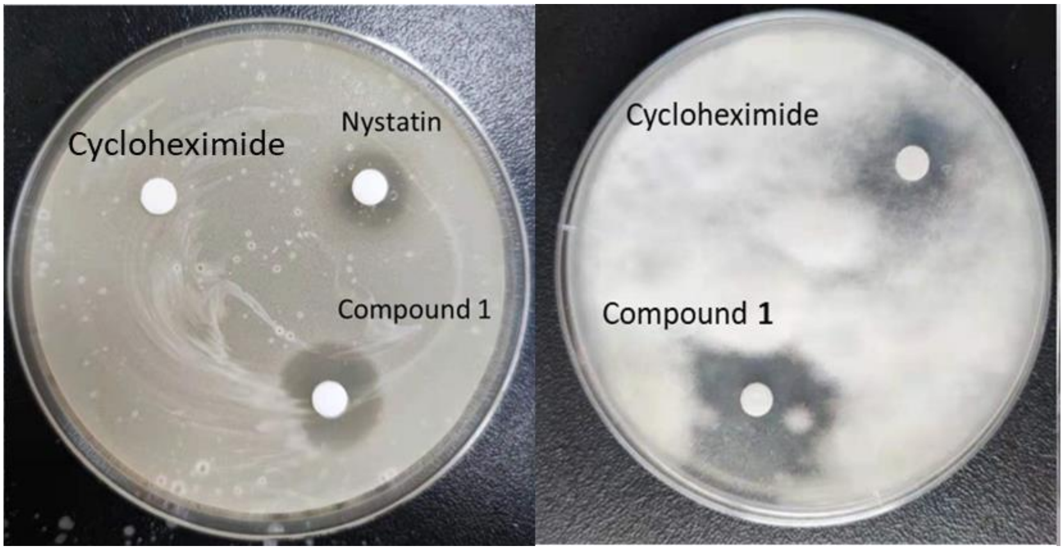

Figure S49. Filter paper agarose diffusion method testing of *Candida albicans* (left) and *Exserohilum turcicum* (right).

ITS sequence:
CGGAGCTACCTTCTACCCATTGTGAACCTTACCTCTTGCCGCGCGTTGCCTCGGCGGGGA
GGCGGGGGTGGGTCGGCGCGCCCCTCTGCGGGCCGCCGTCCCCGCCCCCGTCCCCGCCG
GCCGCGCCAAACTCTAAATTTGCAAAGCGGACTGCATGTTCTGATTTAAAACAAAAAACA
AGTAAAAACTTTTAACAACGGATCTCTTGGTTCTGGCATCGATGAAGAACGCAGCGAAAT
GCGATAAGTAATGTGAATTGCAGAATTCAGTGAATCATCGAATCTTTGAACGCACATTGCG
CCCGGCAGCAATCTGCCGGGCATGCCTGTCCGAGCGTCATTTCTCCCCTCGAGCGCGGCT
AGCCCTACGGGGCCTGCCGCCGCCCGGTGTTGGGGCTCTACGGGTGGGGCTCGTCCCCCC
CGCAGTCCCCGAAATGTAGTGGCGGTCCAGCCGCGGCGCCCCCTGCGTAGTAGATCCTAC
ATCTCGCATCGGGTCCCGGCGAAGGCCAGCCGTCGAACCTTCTTACTCATGGTTTGACCT
CGGATCAGGTAGGGTTACCCGCTGAACTTAAGCATATCAAAAGGCGGAGGAAAAAACCA
TTTTTT

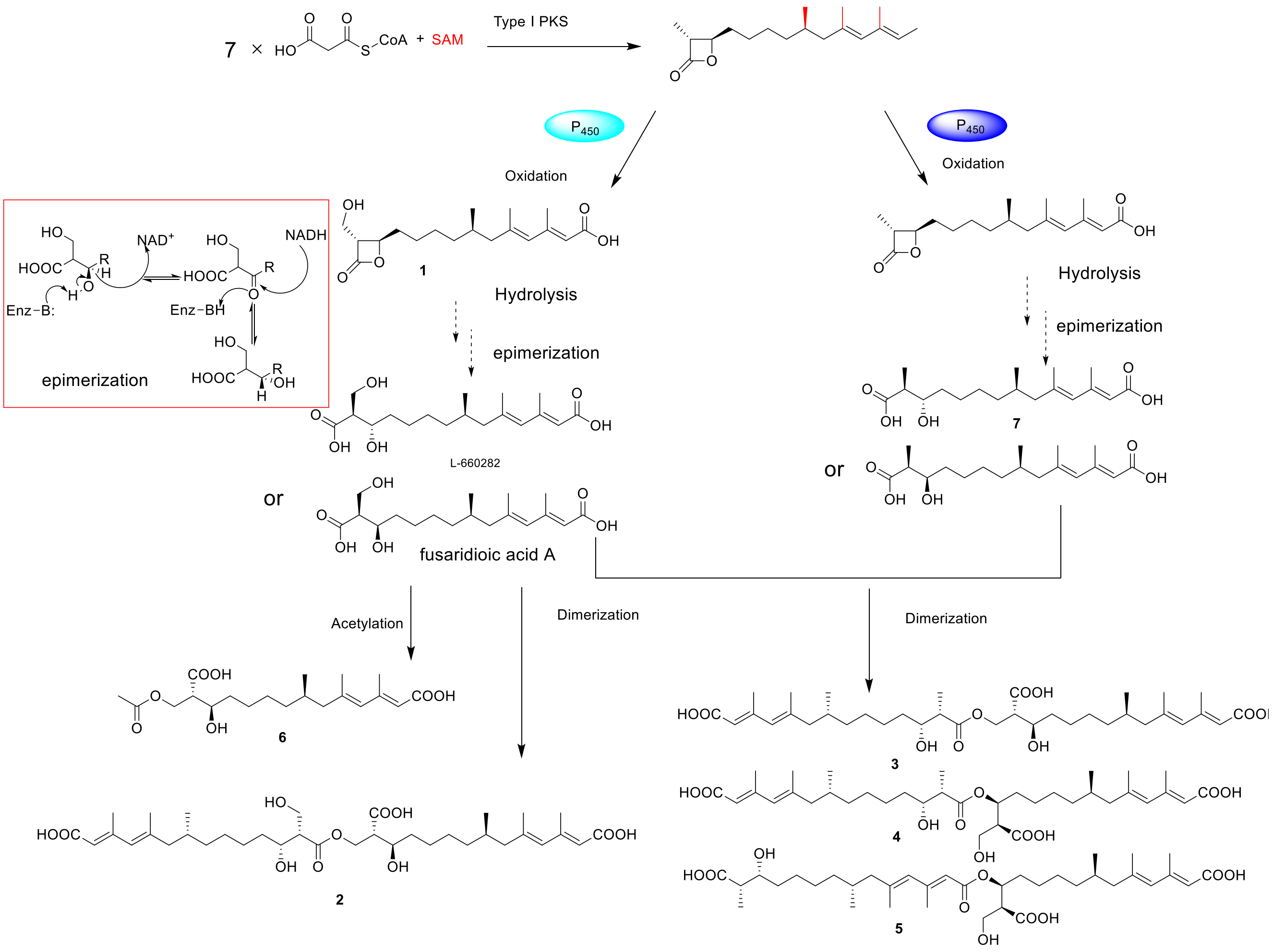

Figure S50. Proposal biosynthesis pathway of compounds **1-7** based on the references (Kumagai et al., 1992;Kato et al., 2020).

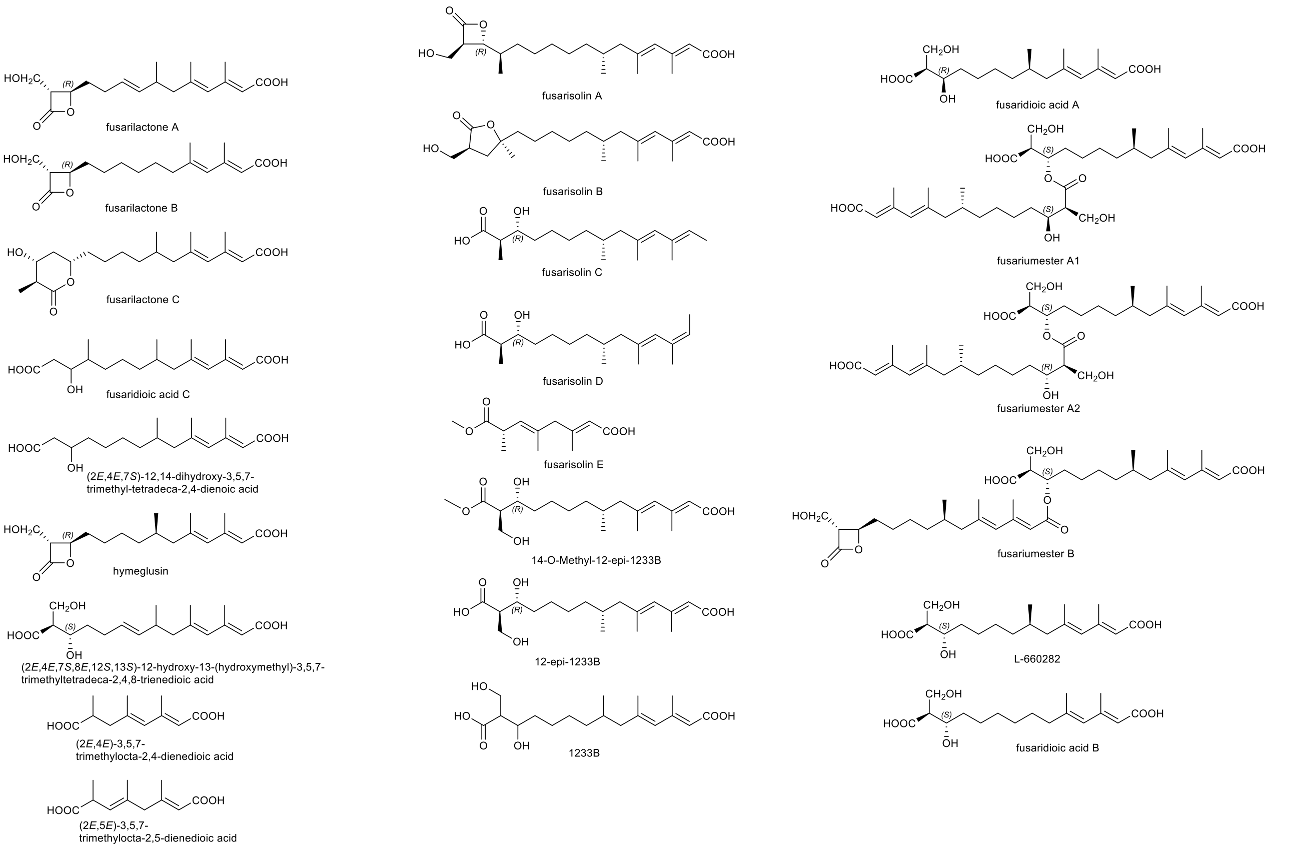

Figure S51. Alkenoic acids reported in the literatures

References:

Kato, S., Motoyama, T., Uramoto, M., Nogawa, T., Kamakura, T., and Osada, H. (2020). Induction of secondary metabolite production by hygromycin B and identification of the 1233A biosynthetic gene cluster with a self-resistance gene. *J Antibiot (Tokyo)* 73**,** 475-479.

Kumagai, H., Tomoda, H., and Omura, S. (1992). Biosynthesis of antibiotic 1233A (F-244) and preparation of [14C]1233A. *J Antibiot (Tokyo)* 45**,** 563-567.
